# Initial Examinations of the Diastereoselectivity and Chemoselectivity of Intramolecular Silyl Nitronate [3+2] Cycloadditions with Alkenyl/Alkynyl Nitroethers

**DOI:** 10.3390/molecules29245816

**Published:** 2024-12-10

**Authors:** Katelyn Stevens, Shik Ki Li, Emily Kaufman, Annika Schull, Katie Hassebroek, Joseph Stevens, Matthew Grandbois, Arlen Viste, Jetty Duffy-Matzner

**Affiliations:** Department of Chemistry and Biochemistry, Augustana University, Sioux Falls, SD 57197, USA

**Keywords:** PM6, dihydrofuro-2-isoxazolines, ISNC, alkenynyl nitroethers, 3-(2,5-dihydrofuryl)carbonyls, chemoselectivity, diastereoselectivity

## Abstract

This study examined the chemoselectivity and diastereoselectivity of silyl nitronate alkenyn-nitroethers in Intramolecular Silyl Nitronate Cycloadditions (ISNCs) to produce isoxazole derivatives with interesting medicinal properties. These reactions resulted in the formation of either dihydrofuro[3,4-c]isoxazolines/isoxazolidines and/or alkynyl moieties attached to 2,5-dihydrofuryl carbonyls. This study also discerned the diastereoselectivities of the resulting cyclic adducts and compared them to previous findings. The reactions were also investigated with Spartan molecular modeling computations to aid in the understanding of any displayed chemo- and/or stereoselectivity. These [3+2]-cycloaddition reactions demonstrated excellent to complete chemospecificity. The cycloadditions also demonstrated remarkable diastereospecificity in that each diastereomer of the nitroethers resulted in the formation of only one of four possible diastereomeric outcomes. The stereochemistry of the major diastereomers did not agree with previously published findings.

## 1. Introduction

**Figure 1:**
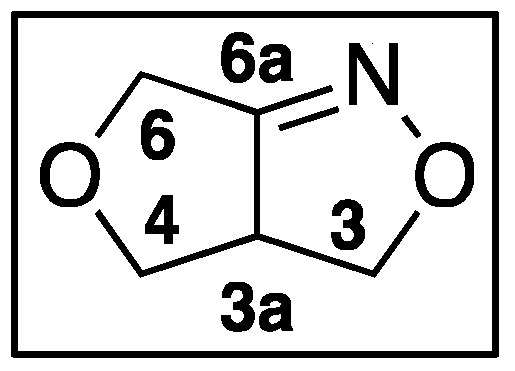
The numbers for carbons in the dihydrofuroisoxazole system following IUPAC rules are displayed. This ring can also be commonly described as a dihydrofuro-2-isoxazoline.

**Scheme 1:**
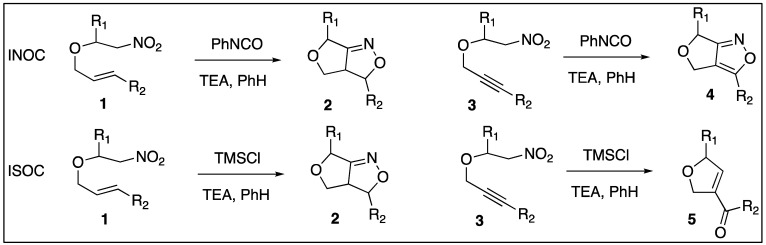
INOC reactions yield the furoisoxazoles/furoisoxazolines with alkenyl- or alkynyl-nitroethers. ISOC reactions will yield either 3-dihydrofuro-carbonyls with the alkynyl-nitroethers or isoxazolines when reacting with alkenyl-nitroethers.

This work questioned whether alkenynl-nitroethers would demonstrate selectivity for either double or triple bonds during [3+2] cycloaddition conditions to prepare isoxazole derivatives. This question has not been investigated to the best of the authors' knowledge. Nitrile Olefin Cycloadditions and Silyl Nitronate Olefin Cycloaddition reactions have been studied extensively by many groups as viable routes for the stereoselective preparation of isoxazoles, isoxazolines, and isoxazolidines [1,2,3,4,5,6,7]. Isoxazoles, isoxazolines, and isoxazolidines are well known for their medicinal value as antibacterial, anticancer, antifungal, antiviral, anti-inflammatory, ectoparasiticide, and anti-tuberculosis agents [8,9,10,11,12]. The Intramolecular Silyl Nitronate Olefin Cycloaddition (ISOC) reaction differs in the reaction of alkenyl- and alkynyl-nitroethers, unlike Intramolecular Nitrile Oxide Olefin Cycloadditions (INOCs), as shown in Scheme 1. Alkenynyl-nitroethers (**1**), when treated under INOC or ISOC conditions, yield the expected 3a,4-dihydro-3*H*,6*H*-furo[3,4-*c*]isoxazoles (**2**). Alkynyl-nitroethers (**3**), when treated under INOC conditions, produce the expected dihydrofuroisoxazoles (**4**) [8,13]. However, alkynyl-nitroethers (**3**) surprisingly yielded 3-dihydrofuranylcarbonyls (**6**) under ISOC conditions [13,14]. Thus, the label ISNC (Intramolecular Silyl Nitronate Cycloaddition) is used by the principal author to describe these reactions with either alkene or alkyne moieties. The common name for an *H*,*H*-isoxazole is isoxazoline, and isoxazolidine is commonly used for *H*,*H*,*H*,*H*-isoxazoles. The numbering for the furoisoxazoles is shown in Figure 1.

**Scheme 2:**
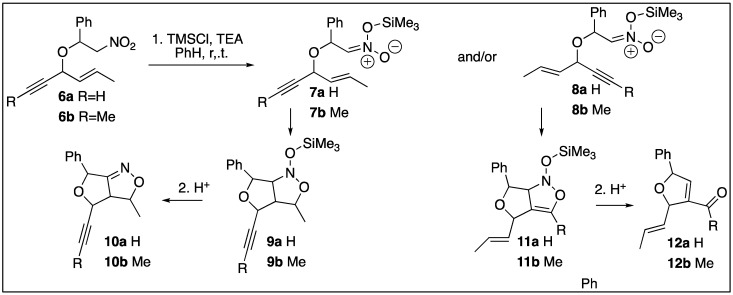
Chemoselectivity options for proposed alkenynyl-nitroethers.

This paper examined both the diastereoselectivity and chemoselectivity of nitroethers in ISNC reactions with alkenyl and alkynyl side chains. This chemoselectivity for the ISNC reactions is displayed in Scheme 2. Trimethylsiloxy-nitronates (**8a**,**b**) are in the correct conformation to undergo ring closing with the double bonds to make N-trimethylsiloxy-dihydrofuro-*3H*,*6H*-isoxazoles (**10a**,**b**) and, consequently, propenyl-furo-4H,6H-isoxazoles (**11a**,**b**) after acidic workup. Trimethylsiloxy-nitronates (**9a**,**b**) form trimethylsiloxy intermediates (**12a**,**b**) that correspond to reactions with the triple bonds. These intermediates produced dihydrofuro-carbonyl products (**13a**,**13b**) after acidic work-up.

**Scheme 3:**
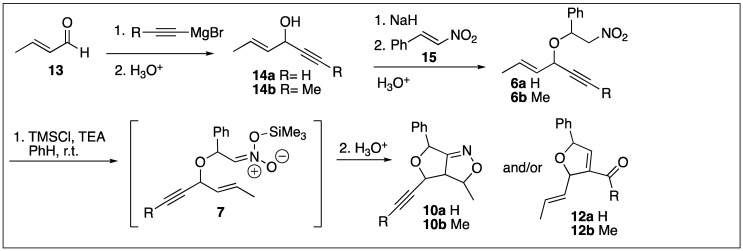
Overall synthetic scheme used to produce the nitroethers and ISNC adducts.

Scheme 3 shows the synthetic scheme used to synthesize and react the alkenynyl-nitroethers. Alkenynols (**14**) were prepared from Grignard reactions with either ethynylmagnesium or propynylmagnesium bromides and crotonaldehyde (**13**). Nitrostyrene (**15**) was prepared in a Henry reaction between nitromethane and benzaldehye [8]. Disappointingly, these allylic/propargylic systems did not react cleanly in the tandem one-pot reaction of olefinic silylnitronates reported by Hassner [15] and Cheng [16]. Complex reaction mixtures were obtained instead. The nitroethers (**5**) were instead prepared via Micheal Additions between sodium alkoxides and unsaturated nitro-compounds [17]. These compounds were then treated to ISNC reactions to yield furo-2-isoxazolines (**10**) and/or carbonyl dihydrofurans (**12**).

Part of this work examined whether alkenynl-nitroethers demonstrated selectivity for either double or triple bonds during ISNC conditions. The ratio of the isoxazole:carbonyl products provided the chemoselectivity control of the alkenynl-silylnitronates. The disasteroselectivities of the cycloadditions were also of interest. Cycloadditions used to form dihydrofuro-2-isoxazolines produce three new stereocenters in the trimethylsiloxy-furoisoxazole intermediates (**9**,**11**) and retain two of the new stereocenters in the products (**10**). The stereochemistry for each enantiomeric pair was labeled with the configurations/name of one of the enantiomers, with a (±) sign in front to indicate the racemic mixture. The identification and diastereoselectivity of the isoxazoline/isoxazolidine and/or furo-carbonyl adducts were primarily determined via NMR experiments (^1^H, ^13^C, COSY, HMQC, NOE). Computational modeling was utilized to support any displayed chemoselectivity and/or diastereoselectivity in the products. NMR information of some compounds included in this research could be found in the Supplementary Materials.

## 2. Results

The synthetic scheme was repeated over the years using several different undergraduate students. Sometimes, the ^1^H NMR analysis of the crude reaction mixture of **10a** would show very small singlet peaks between 9 and 10 ppm. This would seem to indicate that a small amount of the dihydrofuro-carbonyl adducts (3%) were formed. However, these crude NMRs did not have the expected doublet (or doublet of doublets) between 6 and 7 ppm that would have indicated the presence of the expected vinylic proton on the tetrahydrofuran ring as based on previous work by the authors [14]. The crude NMR for **10a** displayed evidence of small amounts of the nitroether, nitrostyrene, and the corresponding alcohol but not any of the expected peaks for the carbonyl compounds other than these singlets. Column chromatography was used to try to isolate compounds for the 9–10 ppm peaks. An analysis of these fractions showed a complex mixture that had many peaks that could not be seen in the beginning crude NMR. Therefore, while some evidence of small amounts of aldehydes was present, the authors are not convinced that the dihydrofuro-carbaldehydes for **10a** were formed. If the authors are incorrect, then this cycloaddition showed excellent chemoselectivity for the double bond (97%). The crude ^1^H NMR for **10b** did not display any evidence of carbonyl products, as no singlets near 2.1 ppm were evident. Based on the crude ^1^H NMR data, both products (**10a**,**b**) resulted from chemospecific reactions of the double bonds over the triple bonds. NOE experiments were used to confirm the major and minor diastereomers that were found for the cycloadditions, as shown in Table 1. These findings show that the “cis” and “trans” diastereomers of the nitroether only produce one diastereomer each in the cycloadditions. Unexpectedly, the assignments of the major and minor diastereomers were switched based on previous findings by the principal author and others.

## 3. Discussion

**Figure 2:**
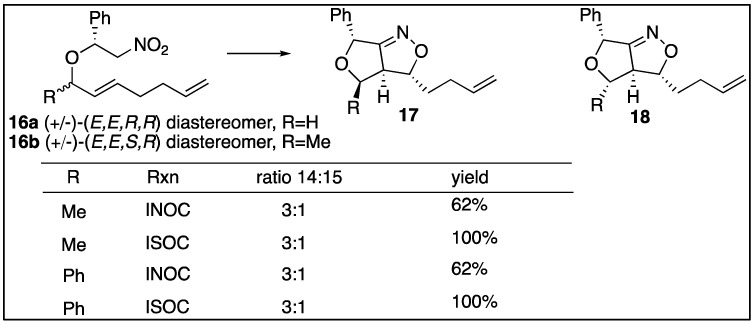
Past evidence of major/minor diastereoisomers’ assignments in ISOC/INOC reactions.

The 1,3-dipolar cycloadditions of the silyl nitronate alkenynyl-nitroethers demonstrated complete chemoselectivity for the double bond over the triple bond and showed unusual results for the stereochemistry of the major/minor diastereomers. The stereochemistry of ISOC/INOC (ISNC) cycloaddition reactions has been extensively studied. The diastereoselectivity of the resulting dihydrofuro-isoxazolidines at C3 and C3a was controlled by the formation of the ring system, and the stereochemistry of the alkene was retained [18]. The relationship of substituents on carbon 4 to the hydrogen on carbon 3a was shown to have a selectively cis relationship (trans hydrogens) if there was no substituent on C6 [8,13,17,18]. Kurth/Duffy showed that the ISOC has complete cis isomer preferences for the hydrogen on C3a and C6 substituents in systems without C3 substituents [19]. Kim’s work [20] with allylic and homoallylic nitroethers gave complete regioselectivity, as predicted by the findings by Duffy [19], but gave a mixture of stereoisomers that under ISOC conditions slightly favored the trans relationship between substituents on C4 and C6 (no substituent on C3). Duffy-Matzner’s investigation into nonterminal propargylic systems also displayed a stronger preference for trans isomers between C4 and C6 [14]. Kurth/Duffy also gave evidence that the major stereoisomer had a trans relationship between C4/C6 substituents in systems with substituents in C3, C4, and C6 positions, as shown in Scheme 2. The assignment of the major diastereomer in the fourth row in Figure 2 was also proven with crystal X-ray evidence for the diphenyl product [18].

**Figure 3:**
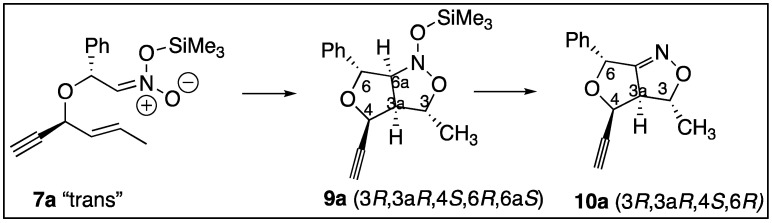
Illustration to show new stereocenters that are prepared as the cycloaddition proceeds.

Thus, it was expected that the major stereoisomer would be the trans-nitroether in this work as well. Trans refers to the stereochemical relationship of the two methine hydrogens alpha to the ether’s oxygen (C4,C6). Unexpectedly, the results of the alkenynl-nitroethers (**6a**,**6b**) showed that the cis-nitroethers were responsible for the stereochemical outcomes of the major diastereomers. As mentioned previously, cycloadditions to form the dihydrofuro-isoxazolidines produced three new stereocenters (C3, C3a, and C6a) in the silyl-nitronate intermediates (**8**) and retained two of the new stereocenters (C3 and C3a) in the products (**10**). The numbering of the ring systems is displayed in Figure 3.

Figure 3 demonstrates that the cycloaddition for the cis-nitroether (*R*,*R*) would produce two new stereocenters at C3 and C3a to give a product with four stereocenters and six in the intermediate. A total of 32 possible diastereomers could be present in the formation of the ring systems from the two nitroethers based on the four stereocenters present in each ring. Since the configurations of C4 and C6 are determined by the formation of the nitroethers, only eight diastereomers are of interest for the newly defined stereocenters. Furthermore, the two hydrogens on C3 and C3a must be trans to each other for the furo-2-isoxazoline due to the configuration of the double bond. This narrows the investigation down to four possible diastereomers for each diastereomer of the nitroether.

Computational studies were performed on the silyl-nitronates (**7a**, **7b**, **8a**, **8b**) to form the trimethylsiloxy-isoxazolidines (**9a**,**b**) and trimethylsiloxy-2-isoxazolines (**11a**,**b**) intermediates. Analyses of these compounds were examined since they have the same number of atoms, which makes it easier to compare the results. It was anticipated that the computational experiments would be challenging as constraints would need to be added to investigate the chemoselectivities of the reactions. Multiple Spartan calculations were employed, and unfortunately, the only calculations that could be utilized with the equipment at hand were semi-empirical (PM6) calculations. The transition state calculations will need further time to be explored at a higher level. The RHF/PM6 transition state optimization procedure utilized Analytical Gradient, Analytical Frequency in TSOPT, Analytical Gradient in FREQ, and Numerical Frequency parameters. Transition states were confirmed by the presence of only one imaginary frequency in the calculated IRs. Hartree Fock (HF) and Density Functional Theory (DFT) calculations will be investigated in the future. A recent review of DFT calculations of 1,3-dipolar cycloadditions demonstrated the utility of the advantages of these reactions but also demonstrated that they have not yet been employed in systems that involve different functional groups for the 1,3-dipole to react with [21]. Semi-empirical PM6 experiments used for the ISNC silyl nitronate intermediates provided the enthalpy of formation data. Some would argue that the semi-empirical quantum chemistry method proposed by James J.P. Stewart calculates the heat of formation of a wide variety of molecules with an accuracy arguably better than Hartree–Fock and better overall than Density Functional Theory [22,23]. This accuracy is accomplished by using experimental parameters along with quantum chemistry calculations. The Hartree–Fock and Density Functional methods rely solely on complex calculations derived from Born–Oppenheimer approximation. Thus, the calculated heats of formation data were trusted to provide good descriptions of the energy differences within the reactions and provide useful trends for this exploratory computational chemistry.

**Figure 4:**
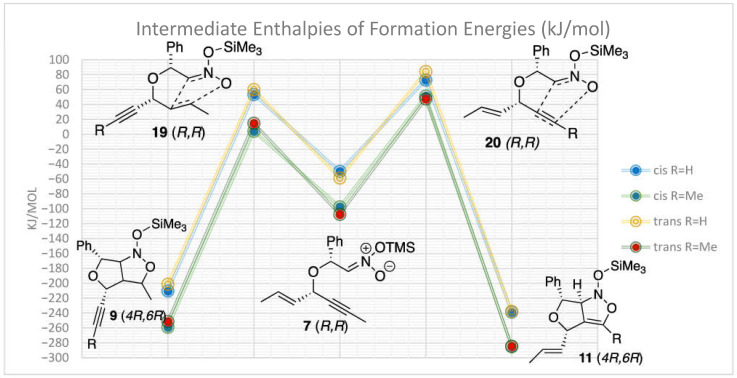
Semi-empirical enthalpies of formation calculations (PM6) used for trimethylsilyl intermediates (kJ/mol).

The computations were run on species generated from “cis” and”trans”-siloxy nitronates. The calculations originated with the lowest energy conformer of the trimethylsilyloxy-nitroether (**7a**). This was then rearranged to reflect the correct orientations for the corresponding transition states (**19a** and **20a**), which were calculated. Finally, the final intermediate trimethylsilyloxy-furoisoxazoline (**11a**) and furoisoxazolidine (**9a**) were examined. The trends can be seen in Figure 4 for the four series. The results showed that the triple bond intermediates (**20a**) had the higher energy transition state with the lowest enthalpy of formation as compared to the transition state involving the double bond (**19a**). This suggests that the formation of the isoxazoline intermediate (**11a**) is favored by thermodynamic conditions. The reactions with the double bond to form the isoxazolidine (**9a**) demonstrated that these products may be formed under kinetic conditions. These calculations suggest that the kinetic products were selectively formed under the experimental conditions for this report. These calculations also displayed that the reactions with the double bond produced a product with a lower transition state energy but a higher enthalpy of formation. This was true independent of the “cis” or “trans” orientation and the substituent placed on the triple bond. It is also interesting to note that the transition states of the terminal alkyne intermediates had higher transition state energies and that the trans-isomers were slightly higher in energy than the cis-diastereomers.

These calculations show that the reaction with the double bond is favored over the triple bond. This explains the chemoselectivity of the reactions and slightly supports the surprising stereoselectivity of the major diastereomers. This was unexpected because cycloadditions of nitroethers have been reported to prefer the (±)-(*3R*,*3aR*,*4S*,*6S*)-furoisoxazolines adducts for silyl nitronate cycloadditions instead of our results reported in Table 1 [9,13,14,15]. The investigation then examined all the possible diastereomers to try to find an explanation.

Four possible diastereomers for the trimethylsiloxy-dihydrofuroisoxazolidine (**9a**) can be expected for one cis-nitroether. These arise from the different faces that are available for the double bond to react with; since the orientation of the C=C bond (alkenyl), and the imino moiety (C=N) of the trimethysiloxy-nitronate can be up or down.

Table 2 illustrates that four possible diastereomers are generated from different orientations of the alkenyl or imino silyl nitronate groups from the cis-nitroether. **I** and **II** show the same face selectivity for all the prochiral centers; this demonstrates that these processes are suprafacial. **III** and **IV** show the prochiral centers reacting from different faces of the system; thus, these cycloadditions are antarafacial. The suprafacial processes demonstrate a less strained transition state for the formation of the new isoxazole and would be favored over the antarafacial ones. Selectivity was also seen in that the si facial attack was favored over the re for the C3a and C6a prochiral centers. The si facial strike led to a chair-like transition state, while the re led to a boat-like transition state. Thus, the major stereoisomer for the cis-nitroether cycloaddition would be the (±)-(*3S*,*3aS*,*4R*,*6R*,*6aR*) silyl intermediate and the (±)-(*3S*,*3aS*,*4R*,*6R*)-adduct. These results agree with the experimental NOE data for the major diastereomer of **10a**. This reasoning is also supported by the PM6 calculations, which clearly show that intermediate I is the lowest energy intermediate. It results from a “chair” transition state with all substituents in “equatorial” positions, as shown in Table 2.

The diastereomer of the trans-nitroether was also considered. Table 3 shows the outcomes of the analysis of its facial selectivity to yield a suprafacial, si transition state. The trans-nitroether (**7a**) would result in the (*3S*,*3aS*,*4S*,*6R*,*6aS*)-silyl intermediate (**9a**) and the (*3S*,*3aS*,*4S*,*6R*)-dihydrofuro-2-isoxazolidine (**10a**). These results agree with the experimental NMR NOE data for the minor diastereomer of **10a**. Similar results were obtained for both the major and minor diastereoisomers (**10b**) of the cycloadditions from the propenyl nitroethers (**7b**). It is unlikely that the stereochemistry at the C4 and C6 positions change during the ISNC process since anionic intermediates are unlikely to experience epimerization rearrangements. This leads the authors to conclude that the formation of the alkenynyl-nitroethers must favor the *cis* orientation for substituents on the carbons alpha to the ether oxygen. This is unlike previously published articles on alkenyl-nitroethers [13] or alkynyl-nitroethers [9,10] that favor the trans orientation.

## 4. Materials and Methods

### 4.1. Physical and Spectral Data

- Low-resolution mass spectra were examined on a Hewlett Packard G1800A GCD system (Agilent Technologies, Santa Clara, CA, USA).
- Proton and carbon NMR spectra were obtained either on a JEOL (400 MHz) spectrometer (JEOL Ltd., Peabody, MA, USA) or a Varian (300 MHz) system (Varian, Inc., Palo Alto, CA, USA). Listed proton NMR data are given in the following order: ppm (multiplicity, coupling constants, integrated number of protons, and assignment). Listed carbon NMR data are given by chemical shift and assignment. All the spectra were run in deuterated chloroform with TMS (Trimethylsilyl) unless stated otherwise.
- Infrared spectra were recorded on a Nicolet Avatar 361 FT-IR (Thermo Nicolet Corporation, Waltham, MA, USA) with Gateway 2000 data system and were either neat NaCl plates (liquids) or KBr pellets (solids).

### 4.2. Chromatography

- Flash column chromatography refers to the resolution technique of W. Clark Still [24]. A glass column is filled with a slurry of dry 40–62 μm silica gel and solvent. The same solvent is used as an eluent to push a sample through the column, with pressure from a nitrogen inlet to speed the elution to a rate of 2 in./min.
- TLC refers to thin-layer chromatography, which was performed on Sigma Chemical Co. (Livonia, MI, USA) plates made of 250 μm silica gel on polyester with a 254 nm fluorescent indicator added. Visualization was performed via iodine chambers or UV lamp.
- Capillary gas chromatography (GC) was performed on an HP G1800A GCMS (Agilent Technologies, Santa Clara, CA, USA), equipped with an HP-5 Crosslinked 5% PH ME Siloxane column (Agilent Technologies, Santa Clara, CA, USA), 30 m × 0.25 mm × 0.25 μm.

### 4.3. Solvents

Tetrahydrofuran was distilled under a nitrogen atmosphere using sodium and stored over 4 Å molecular sieves. Hexane was distilled under a nitrogen atmosphere using sodium and stored over 4 Å molecular sieves. Benzene was distilled under a nitrogen atmosphere using sodium and stored over 4 Å molecular sieves.

### 4.4. Reactions

- Concentration under reduced pressure refers to solvent removal on a Büchi RE 111 rotary evaporator (BüCHI Labortechnik AG, Flawil, Switzerland) connected to a water aspirator and an ethylene glycol cooling system.
- All reactions were run under a nitrogen atmosphere unless otherwise stated.
- Unless otherwise stated, all other solvents and reagents were reagent grade and used without further purification.

### 4.5. General Procedures

#### 4.5.1. General Procedure A: Alkenynols

The Grignard reagent (0.1 M, 1.2 mol) was placed in a round-bottomed flask with a stir bar under nitrogen. Then, dry THF was added to dilute the solution (0.01 M) while the flask was in an ice bath, and crotonaldehyde (neat, 1 mol) was added dropwise through a syringe. After the addition was complete, the solution was slowly allowed to reach room temperature. At this point, TLC showed no starting material. The reaction was quenched with HCl (3 M, 2 mol). Diethyl ether was added. The organic and aqueous layers were separated. The aqueous layer was extracted three times with diethyl ether. The combined organic layers were washed with 5% sodium bicarbonate and brine and then dried over magnesium sulfate. After gravity filtration, the product was reduced under vacuum to yield an oil.

(4*E*)-hex-4-en-1-yn-3-ol (**14a**):

General procedure A: vacuum distillation (0.1 Torr) yielded 5.465 g (54.65%) of **14a** as a clear pale-yellow liquid from 6.48 g (0.09247 mol) of crotonaldehyde (**11**). [FTIR (neat): 3300 cm^−1^ (OH *v*), 3032 (=C-H *v*), 2975, 2941 (C-H asym *v*), 2879 (C-H sym *v*), 2159 (C≡C *v*), 1447, 1378 (C-H δ), 1016 (COC *v*), 964 (oop δ trans CH=CH)]; [^1^H NMR (400 MHz, CDCl_3_): δ = [1.75 ppm (m, 3H, CH_3_), 1.96 (s, 1H, OH), 2.57 (d, J = 1.6 Hz, 1H, C≡C-H), 4.83 (m, 1H, CH-O), 5.64 (ddq, J = 15.3, 6.4, 1.6 Hz, 1H, CH=CH-CH_3_), 5.94 (dqd, J = 15.3, 6.4, 1.6 Hz, 1H, =CH-CH_3_)]. [^13^C NMR (100 MHz, CDCl_3_): δ = [17.4 ppm (CH_3_), 62.3 (CH-O), 73.8 (C≡C-H), 83.7 (C≡C-H), 128.6 (CH_3_-CH=CH), 130.1 (CH_3_-CH=CH)]; GCMS (M^+^ C_6_H_8_O 96.15 m/z).

(*E*)-5-heptyn-2-en-4-ol (**14b**):

General procedure A yielded 10.9 g (82% yield) of **14b** as dark brown oil from 8.468 g (0.1207 mol) of crotonaldehyde (**16**). [R_f_ = 0.50, (1:4 EtOAc: Pentane), [FTIR (neat): 3363 cm^−1^ (OH ν), 2919 (sp^3^ C-Hν)), 2237 (C≡Cν)), 1673 (CH=CHδ); [^1^H NMR (300 MHz, CDCl_3_): δ 1.73 ppm (m, 3H, CH_3_-CH=), 1.86 (d, J = 1.8Hz, 3H, CH_3_- C≡), 3.26 (s, 1H. OH), 4.77 (m, 1H, CH-OH), 5.60 (m, 1H, =CH-CHOH), 5.84 (dq, J = 15, 6.6Hz, 1H, CH_3_-CH=)]; [^13^C NMR (75.4 ppm MHz, CDCl_3_) δ = 3.6 (CH_3_-C≡C), 17.4 (CH_3_-C=), 62.9 (CH-OH), 79.3 (CH_3_-C≡C), 81.9 (C≡C-CHOH), 127.9 (=CH-CH_3_), 131.2 (=CH-CHOH)]; GCMS (M^+^ C_7_H_10_O 110.05 m/z).

#### 4.5.2. General Procedure B: Nitroethers (6)

Sodium hydride (0.21 mol) was washed with distilled hexanes (15 mL, 5 times) in a dry round-bottomed flask with a stir bar and dried under nitrogen. Alkenynol (0.20 mol) was added slowly at room temperature. The flask was placed in a dry ice and isopropanol bath at −30 °C bath. Nitrostyrene was prepared using the Henry reaction method published by Gairaud [24]. A solution of nitrostyrene in THF was prepared (0.1 M). Using a syringe pump set at 20 mL/hour, nitrostyrene was added to the alkoxide solution (0.10 mol). When all the nitrostyrene was added, the reaction was quenched with HCl (3 M, 0.3 mol). At this point, TLC showed no starting material. The aqueous layer was extracted three times with diethyl ether (30 mL). The organic layer was then dried over magnesium sulfate. After gravity filtration, the solution was reduced under vacuum to yield an oil. The nitroether was purified through column chromatography in a solvent of 1:9 ethyl acetate:hexanes that slowly transitioned to 1:6. Unfortunately, clean nitroethers tended to under retro-Micheal Additions and were not suitable for elemental or HRMS analysis.

(±)-(*3R*,*4E*)-3-[(*1R*)-2-nitro-1-phenyleth-1-oxy]hex-4-en-1-yne (**6a**):

General Procedure B yielded **6a** as a yellow oil 1.970 g in a crude 100% yield. After column chromatography, **6a** was isolated as a yellow oil, 1.611 g (81.8%) from 1.198 g (0.008032 mol) of nitrostyrene (**18**) as two inseparable diastereomers (1.5:1 ratio): I (±)-(*3R,4E*)-3-[(*1R*)-2-nitro-1-phenyleth-1-oxy]hex-4-en-1-yne, II. (±)-(*3S,4E*)-3-[(*1R*)-2-nitro-1-phenyleth-1-oxy]hex-4-en-1-yne. [R_f_ = 0.40, (1:6 EtOAc: hexanes)]; FTIR (KBr): [3290 cm^−1^ (≡CHν), 3050 (sp^2^ C-Hν), 2940, 2919 (sp^3^ C-Hν), 2100 (C≡Cν), 1556, 1380 (NO_2_ν), 1061 (C-O-Cν)]; ^1^H NMR (400 MHz, CDCl_3_): δ = [1.70 ppm (d, J = 6.5 Hz, 0.40H, =C-CH_3_ II), 1.75 (d, J = 6.5 Hz, 0.60H =C-CH_3_ I), 2.45 (d, J = 2.4 Hz, 0.60H, H-C≡C I), 2.59 (d, J = 2.4 Hz, 0.40H, H-C≡C II), 4.42 (m, 1.2H, O-CH-C=C I, CHNO_2_ I), 4.42 (m, 0.80H, O-CH-C=C II, CHNO_2_ II), 4.69 (m, 1H, CHNO_2_ I&II), 5.26 (dd, J = 10.4, 3.2 Hz, 0.60H, CH-Ph I), 5.48 (m, 1.40H, CH=CH-CH_3_ I&II, CH-Ph II), 5.74 (dq, J = 15.2, 6.4 Hz, 0.60H, =CH-CH_3_ I), 5.86 (dq, J = 15.2, 6.4 Hz, 0.40H, =CH-CH_3_ II), 7.39 (m, 5H, Ph-H, I&II).] [^13^C NMR (100 MHz, CDCl_3_): δ = [17.6 ppm (CH_3_ I&II), 67.5 (CH-C≡C II), 68.9 (HC-C≡C I), 74.7 (H-C≡C I)**,** 75.3 (HC-Ph I), 75.6 (HC-Ph II), 75.8 (H-C≡C II), 79.8 (H-C≡C II), 80.1 CH_2_-NO_2_ I&II), 81.1 (H-C≡C I), 125.9 (CH=CH-CH_3_ II, 129.1, 129.2, 129.3 (Ar CH), 130.6 (=CH-CH_3_ II), 132.0 (=CH-CH_3_ I), 135.7 (Ar C II), 136.2 (Ar C I)].

(±)-(*2E*,*4R*)-4-[(*1R*)-2-nitro-1-phenyleth-1-oxy]hept-2-en-4-yne (**6b**):

General Procedure B yielded a crude yellow oil with a large amount of starting alcohol present. After column chromatography, **6b** was isolated as a white powder, 2.2054 g (82%) from 1.6355 g (0.01096 mol) of nitrostyrene (**15**) as two inseparable diastereomers (1.2:1): I. (±)-(*2E,4R*)-3-[(*1R*)-2-nitro-1-phenyleth-1-oxy]hept-2-en-4-yne, II. (±)-(*2E,4S*)-3-[(*1R*)-2-nitro-1-phenyleth-1-oxy]hept-2-en-4-yne. [R_f_ = 0.23 (1:10 EtOAc:Hexane)]; FTIR (KBr) [3032 cm^−1^ (sp^2^ C-H ν), 2979, 2919, 2875 (sp^3^ C-H ν), 2251 (C≡C ν), 1557, 1381 (NO_2_ ν), 1086 (C-O-C ν)]. ^1^H NMR (500 MHz, CDCl_3_): δ= [1.69 ppm (d, 6.5 Hz, 0.34H, CH_3_-C= II), 1.74 (d, J = 6.5 Hz, 0.64H, CH_3_-C= I),1.76 (d, J = 2.0 Hz, 0.64H, CH_3_-C≡C I), 1.90 (d, J = 1.5 Hz, 0.36H, CH_3_-C≡C II), 4.40 (m, 1.64H, =C-CH-O I, CH-NO_2_ I&II), 4.68 (m, 1.64H, CH-NO_2_ I, CH-CPh I&II), 5.24 (dd, J = 9.5 Hz, 3.5 Hz, 0.64H, CH-C≡C I), 5.44 (m, 0.72H, =CH-CHO II, CH-C≡C II), 5.50 (m, 0.64H, =CH-CHO I), 5.68 (dq, J = 15.0 Hz, 6.5 Hz, 0.64H, CH_3_-CH=CH I), 5.81 (dq, J = 15.0, 6.5 Hz, 0.34H, CH_3_-CH=CH II), 7.38 (m, 5H, ArH, I&II]. [^13^C NMR (75.4 MHz, CDCl_3_) δ = [3.6 ppm (CH_3_-C≡ I), 17.4 (CH_3_-C= I), 68.1 (CH-Ph II), 69.4 (CH-Ph I), 74.9 (=C-CH-O I), 75.4 (C≡CH I), 76.7 (C≡CH II), 80.1 (CH_2_-NO_2_ I), 80.3 (CH_2_-NO_2_ II), 83.2 (C≡CH I), 84.1(C≡CH II), 126.9, 127.0 (CH Ar), 128.0 (=CH-CH-O I), 128.1 (=CH-CH-O II), 128.8, 128.9, 129.0, 129.1 (Ar CH I), 129.7.0 (=C-CH_3_ II), 131.0 (=C-CH_3_ I), 136.3 (Ar C II), 136.6 (Ar C I)].

#### 4.5.3. General Procedure C: Intramolecular Silyl Nitronate Cycloaddition (ISNC)

Nitroether (0.1 mol) was dissolved in enough dried benzene to make a 0.2 M solution under nitrogen and with a stir bar in a dry, round-bottom flask. Distilled triethylamine (0.2 mol) and then tetramethylsilylchloride (TMSCl) (0.2 mol) were added to the solution, and the nitrogen lead was removed. The solution was stirred for 2 days at room temperature, and TLC showed no evidence of the nitroether. Hydrochloric acid (1 M, 0.4 mol) was added, as well as some diethyl ether. The organic and aqueous layers were separated. The aqueous layer was extracted with diethyl ether 3 times (30mL per extraction). The combined organic layer was washed with 5% sodium bicarbonate and brine. The solution was dried with anhydrous magnesium sulfate, filtered, and concentrated first with a rotatory evaporator and then a vacuum pump.

(±)-(3,3a-dihydro-3-methyl-6-phenyl-4-[eth-1-ynyl]-*4H*,*6H*-furo[3,4-c]isoxazole (**10a**).

General procedure C gave 0.930 g of the crude **10a** (2.1:1 ratio of diastereomers via ^1^H NMR) with THF still visible. It was then treated with column chromatography to yield an inseparable mixture of the two diastereomers **8a** (I and II) (0.8147 g, 89.3%) from (0.985 g, 0.004016 mol) **6a**. Solvent washing and a large plate TLC (1:6 EtOAc:hexanes) allowed a small portion of each diastereomer to be isolated for NMR analysis.

**10a I.** (±)-(*3S*, *3aS*, *4R*, *6R*)-3,3a-dihydro-3-methyl-6-phenyl-4-[eth-1-ynyl]-*4H*,*6H*-furo [3,4-c]isoxazole.

R_f_ = 0.27 (1:6 EtOAc:hexanes); GCMS (7.51 min, ramp 30, 60–280 °C) 51, 77, 103, 130, 227 M^+^; FTIR (KBr pellet) 3236 cm^−1^ (≡C-H *v* cm^−1^), 3050 (=C-H *v*), 2983, 2972 (C-H asym *v*), 2894 cm^−1^, 2857 (C-H sym *v*), 2126 (C≡C *v*), 1457 (C=N-O *v*), 1006 (C-OC v), 968 (*oop* δ trans C=C), 757,704 (*oop* δ monosub Ph); ^1^H NMR [400 MHz, CDCl_3_]: I δ = 1.55 ppm (d, J = 6.4 Hz, 3H, CH_3_), 2.67 (d, 2.0 Hz, 1H, H-C≡C), 3.86 (m, 1H, CH-C=N), 4.64 (dd, J = 9.2, 2.0 Hz, 1H, CH- C≡C), 4.72 (dq, 11.6, 6.4 Hz, 1H, CH-CH_3_), 5.63 (apparent s, 1H, CH-Ph), 7.40 (m, 5H, Ar CH). [^13^C NMR (100 MHz, CDCl_3_) δ = 18.3 ppm (CH_3_), 65.2 (CH-C=N), 69.6 (CH-C≡C), 74.0 (CH-Ph), 75.9 (C≡C-H), 79.6 (C≡C-H), 83.0 (CH-CH_3_), 125.8, 128.6, 128.7 (Ar CH), 136.9 (Ar C), 169.4 (C=N).] HRMS C_14_H_13_NO_2_ [M^+^]. Calculated 227.09464; found 227.09464.

**10a II**. (±)-(*3S*, *3aS*, *4S*, *6R*)-3,3a-dihydro-3-methyl-6-phenyl-4-[eth-1-ynyl]-*4H*,*6H*-furo[3,4-c]isoxazole.

R_f_ = 0.26 (1:6 EtOAc:hexanes); GCMS (7.80 min, ramp 30, 60–280 °C), 51, 77, 103, 130, 227 M^+^; FTIR (KBr pellet) 3236 cm^−1^ (≡C-H *v* cm^−1^), 3066 (=C-H *v*), 2983, 2920 (C-H asym *v*), 2855 (C-H sym *v*), 2109 (C≡C *v*), 1446 (C=N-O *v*), 1008 (C-OC v), 967 (*oop* δ trans CH=CH), 758 (*oop* δ monosub Ph); ^1^H NMR [400 MHz, CDCl_3_]: δ = 1.54 ppm (d, J = 6.4 Hz, 3H, CH_3_), 2.82 (d, 1.6 Hz, 1H, H-C≡C), 3.95 (ddd, J = 10.0, 8.4, 1.2 Hz, 1H, CH-CHCH_3_), 4.98 (dq, J = 11, 6.4 Hz, 1H, CH-CH_3_), 5.07 (dd, J = 8.4,1.6 Hz, 1H, CH-C≡C), 5.68 (apparent s, 1H, CH-Ph), 7.36 (m, 5H, Ar CH). [^13^C NMR (100 MHz, CDCl_3_) δ = 18.4 ppm (CH_3_), 62.8 (CH-C=N), 67.7 (CH-C≡C), 72.6 (CH-Ph), 78.0 (C≡C-H), 79.6 (C≡C-H), 82.3 (CH-CH_3_), 125.9, 128.6, 128.8 (Ar CH), 136.4 (Ar C), 169.3 (C=N)]. Calculated 227.09464; found 227.09464.

(±)-3,3a-dihydro-3-methyl-6-phenyl-4-[prop-1-ynyl]-*4H,6H*-furo[3,4-c]isoxazole (**10b**).

General Procedure C yielded crude isoxazole (1.8:1 ratio of diastereomers via ^1^H NMR) as a yellow oil, 1.869 g. Column chromatography yielded **10b,** clean but inseparable mixture (1.86:1) of the two diastereomers as a yellow oil, 1.753 g (81% yield) from 1.8843 g (0.007266 mol) of **5b**.

**10b I**. (±)-(*3S*, *3aS*, *4R*, *6R*)-3,3a-dihydro-3-methyl-6-phenyl-4-[prop-1-ynyl]-*4H,6H*-furo[3,4-c]isoxazole. **10b II**. (±)-(*3S*, *3aS*, *4S*, *6R*)-3,3a-dihydro-3-methyl-6-phenyl-4-[prop-1-ynyl]-*4H,6H*-furo[3,4-c]isoxazole.

R_f_ = 0.628, 0.545 (1:4 EtOAc: Pentane); FTIR (NaCl) 3032 cm^−1^, 3063 (sp^2^ C-H ν), 2960, 2922, 2873 (sp^3^ C-H ν), 2254 (C≡C ν), 1602 (Ar C=C ν), 1051 (C-O-C ν); [^1^H NMR (300 MHz, CDCl_3_): δ = [1.54 (d, J = 6.4 Hz, 1.05H, CH_3_-CH II), 1.55 ppm (d, J = 6.4 Hz, 1.95H, CH_3_-CH I), 1.91 (d, J = 2.4 Hz, 1.95H, CH_3_-C≡C I), 1.97 (d, 2.8 Hz, 1.05H, CH_3_-C≡C II), 3.79 (ddd, J = 11.0, 9.3, 1.5 Hz, 0.65H, CH-C=N I), 3.90 (ddd, J =10, 7.8, 1.7 Hz, 0.35H, CH-CH-C=N II II), 4.67 (dq, J =11, 2.0 Hz, 0.65H, CH-C≡C I I), 4.69 (dq, J = 11, 6.4 Hz, 0.65H, CH-CH_3_ I), 4.95 (dq, J =10, 6.4Hz, 1H, CH-CH_3_ II), 5.06 (m, 0.35H, CH-C≡C II), 5.59 (apparent s, 0.65H, CH-Ph I), 5.68 (m, 0.35H, CH-Ph II), 7.40 (m, Ar CH I&II)]; [^13^C NMR(75 MHz, CDCl_3_) δ= [ 3.7 ppm (CH_3_-C≡C I&II), 18.3 (CH_3_-CH II), 18.4 (CH_3_-CH I), 63.2 (CH-C=N II), 65.6 (CH-C=N I), 67.7 (CH-C≡C II), 68.4 (CH-C≡C I), 70.5 (CH-Ph II), 72.4 (CH-Ph II), 73.9 (CH-Ph I), 75.1 (C≡C-CH_3_ I&II), , 82.3 (CH-CH_3_ II), 82.9 (CH-CH_3_ I), 84.5 (C≡C-CH_3_ I), 88.3 (C≡C-CH_3_ II), 126.0, 126.0, 128.5, 128.7 (Ar CH 1 & II), 136.8 (Ar C II), 137.2 (Ar C I), 137.5 (Ar C I), 170.0 (C=N II), 170.3 (C=N I)]; GCMS M^+^ 241.10; HRMS for C_15_H_15_NO_2_ [M+H]. Calculated 242.1181; found 242.1181.

## 5. Conclusions

This work examined the chemoselectivity and diastereoselectivity of alkenynyl-nitroethers in Intramolecular Silyl Nitronate Cycloadditions (ISNCs). Two different systems were tested in the alkenyl-nitroethers: terminal and non-terminal alkynes. The authors conclude that these two systems were chemospecific for the reaction of the double bonds over the triple bonds. Spartan semi-empirical (PM6) analyses indicated that the chemoselective reactions could be controlled under kinetic and/or thermodynamic conditions and that the kinetic products were selectively formed under room-temperature experimental conditions. The authors assumed that the major diastereomers for the two systems would have (±)-(*4S*,*6R*)-configurations. Instead, the results indicate that the major diastereomers for the two systems instead have (±)-(*4R*,*6R*)-configurations. Therefore, the alkenynyl-nitroethers formation reactions must choose the *cis* orientation for the methines alpha to the ether oxygen over the *trans* for the major diastereomer. This is hard to prove since the nitroethers (oils) readily undergo retro-Michael Additions, and the non-cyclic structures are not favorable for NOE analysis. However, since epimerization is extremely unlikely with the anionic intermediates, the stereochemistry of the C4 and C6 carbons of the ring systems must be set by the nitroethers. Analyses of the four possible transition states that could be formed by each nitroether diastereomer agree with the stereochemical outcomes found via NOE analysis of the cycloaddition adducts. This leads the authors to conclude that the reaction of each diastereomer was diastereospecific in that only one of the four diastereomers was formed. Thus, the ISNC reactions of these alkenynyl-nitroethers were found to be both chemospecific and diastereospecific. Since the stereochemical results were unexpected, the authors intend to further investigate the outcomes of these reactions with a variety of electron-donating and electron-withdrawing groups present at C4 and C6 for furoisoxazolines and furoisoxazolidines. Further studies will also be conducted at the DFT (B3LYP and ωB97X-D) levels to investigate the transitions states since these calculations provide much stronger calculations for the imaginary frequencies and methods to examine the HOMO/LUMO differences for these pericyclic reactions. Nitronates participating in [3+2] cycloadditions have also been investigated recently via DFT calculations. Kącka-Zych examined the reactions of amine oxides with nitroalkenes in bimolecular reactions [25]. The same authors also investigated the intramolecular reaction of N-trialkylsiloxy nitronates with alkenes [26]. They discovered that the reaction proceeded with two pseudocyclic elemental reactions: nitronate-[3+2] cycloaddition and the loss of trimethylsilanol to make a nitrile oxide, which then proceeded in a cycloaddition reaction. These two pathways will also be explored in future studies with our intramolecular alkenynyl trimethylsiloxy nitronates via synthetic and computational methods.

## Figures and Tables

**Table 1:** Stereochemical outcomes for the major/minor diastereomers of the ISNC reactions.

10a Major	10a Minor	10b Major	10b Minor
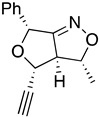	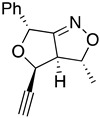	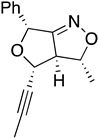	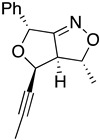
(±)-(*3R*,*3aR*,*4R*,*6R*)	(±)-(*3R*,*3aR*,*4S*,*6R*)	(±)-(*3R*,*3aR*,*4R*,*6R*)	(±)-(*3R*,*3aR*,*4S*,*6R*)

**Table 2:** Four possible intermediates for the cis-nitroether (**7a**). The third row lists enthalpies of formation for the cyclic intermediates based on PM6 calculations.

**I** *3R 3aR 4R 6R 6aS*	**II** *3S 3aS 4R 6R 6aR*	**III** 3*S 3aS 4R 6R 6aS*	**IV** *3R 3aR 4R 6R 6aR*
Intermediates			
∆H_f_˙= −210.26 kJ/mol	−189.11 kJ/mol	−132.63 kJ/mol	−117.22 kJ/mol
** 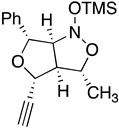 **	** 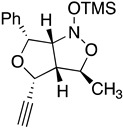 **	** 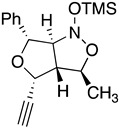 **	** 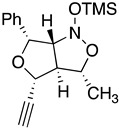 **
Transition states			
‡ alkene up/imino up	‡ alkene down/imino down	‡ alkene down/imino up	‡ alkene up/imino down
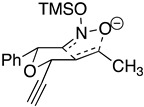	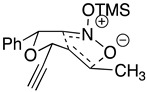	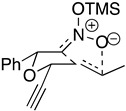	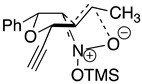

**Table 3:** Justification for the major furoisoxazoline diastereomers of the trans-nitroether. (±)-(*3S*,*4E*)-3-[(*1R*)-1-phenyl-2*-O*-trimethylsilylnitro)-2-ethoxy]hex-4-en-1-yne, **7a**.

Rxn Scheme	Transition State
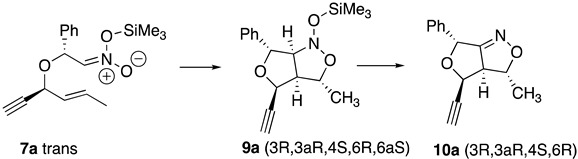	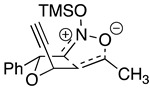

## Data Availability

All data are provided in Supplementary Materials.

## Supplementary Materials

The following supporting information can be downloaded at: https://www.mdpi.com/article/10.3390/molecules29245816/s1, ^1^H NMR, ^13^C{^1^H} NMR information is provided for compounds **6a**, **6b**, **10a**, and **10b**. **14a**, **14b** COSY, DEPT, and HMQC information is provided for compounds **6a**, **6b**, **10a**,& **10b**. NOE information is provided for **10a**,**b**. Also, a crude ^1^H NMR is provided for **10a**. PM6 computational methods and information are provided for cis/trans compounds: **6a/b**, **7a/b**, **9a/b**, **11a/b**, 1**9a/b**, &**20a/b**, as well as for cis studies of **10a**, **I**, **II**, **III**, **&IV**.
