# Supplementary material for: Initial Examinations of the Diastereoselectivity and Chemoselectivity of Intramolecular Silyl Nitronate [3+2] Cycloadditions with Alkenyl/Alkynyl Nitroethers
Source: Molecules. 2024 Dec 10;29(24):5816. doi: 10.3390/molecules29245816 (PMC11679677; doi:10.3390/molecules29245816)
Supplement: Supplementary file 1 [file molecules-29-05816-s001.zip › molecules-3298913-supplementary.pdf]

Supporting Information

Initial Examinations of the Diastereoselectivity and Chemoselectivity of the Intramolecular Silyl Nitronate Cycloadditions with Alkenyl/Alkynyl Nitroethers

Katelyn Stevens, Shik Ki Li, Emily Kaufman, Annika Schull, Katie Hassebroek, Matthew Grandbois, Joseph Stevens, Arlen Viste, and Jetty Duffy-Matzner*

Department of Chemistry and Biochemistry, Augustana University, Sioux Falls, SD 57197
*jetty.duffy@augie.edu

^{1}H NMR, ^{13}C{^{1}H} NMR information is provided for compounds **6a**, **6b**, **10a, 10b, 14a, & 14b**. COSY, DEPT and HMQC information are provides for compounds **6a**, **6b**, **10a,** & **10b**. NOE information provided for 10a & 10b. PM6 computational methods and information are provided for *cis/trans* compounds: **7a, 7b**, **9a, 9b**, **11a, 11b**, **19a, 19b**, **20a, & 20b.** It is also listed for *cis* studies of **6ab**, **10ab, 12ab**; & for **I**, **II**, **III**, & **IV.**

Table of Contents Page

^{1}H NMR for (±),-(*E*,*R*,*R*)-3-[2-nitro-1-phenyleth-1-oxy]hex-4-en-1-yne (**6a)** ………….. 3
^{13}C{^{1}H} NMR for **(6a)** …………………………………………………………. 4
HMQC for **(6a)** ……………………………………………………………….. 5
COSY for **(6a)** ………………………………………………………………..…. 6
DEPT for (**6a**) ………………………………………………………………..….. 7
^{1}H NMR for (±),(±)-(*E*,*R*,*R*)-3-[2-nitro-1-phenyleth-1-oxy]hept-2-en-4-yne (**6b)** ……. 8
^{13}C{^{1}H} NMR for **(6b)** …………………………………………………………. 9
HMQC for **(6b)** ………………………………………………………………. 10
COSY for **(6b)** ………………………………………………………………..…. 11
DEPT for (**6b**) ………………………………………………………………..…. 12
^{1}H NMR for (±)-(*3R,3aR,4R/S,6R*)-(3,3a-dihydro-3-methyl-6-phenyl-4-[eth-1-ynyl]-*4H,6H*-furo[3,4-c]isoxazole (**10a)**
major diastereomer I ………………………………………………………… 13
minor diastereomer II ……………………………………………………….. 14
^{13}C{^{1}H} NMR for (**10a)**
major diastereomer I ………………………………………………………… 15
minor diastereomer II ……………………………………………………….. 16
HMQC for **(10a)**
major diastereomer I ………………………………………………………… 17
minor diastereomer II ……………………………………………………….. 18
COSY for **(10a)**
major diastereomer I ………………………………………………………… 19
minor diastereomer II ……………………………………………………….. 20
DEPT for (**10a**) ………………………………………………………………..…. 21
NOE for **(10a)**
major diastereomer I ………………………………………………………… 22
minor diastereomer II ……………………………………………………….. 23
Crude ^{1}H NMR (**10a**) ……………………………………………………… 24
^{1}H NMR for (±)-(*3R,3aR,4R/S,6R*)-(3,3a-dihydro-3-methyl-6-phenyl-4-[prop-1-ynyl]-*4H,6H*-furo[3,4-c]isoxazole (**10b)** ……………………………………………….. 25

$^{13}C\{^{1}H\}$ NMR for (**10b)** …………………………………………………………………. 29
HMQC **(10b)** ……………………………………………………………………………. 30
COSY **(10b)** ……………………………………………………………………..…. 31
DQF COSY for (**10b**) ……………………………………………………………………… 32
NOE **(10b)** ……………………………………………………………………………. 33
^{1}H NMR for (*E*)-hex-4-en-1-yn-3-ol **(14a**) ………………………………………….…. 34
^{1}H NMR for (*E*)-5-heptyn-2-en-4-ol (**14b**) ………………………………………….…. 35
Computational Methods……………………………………………….…………. 36
PM6 Cartesian Coordinates & info for *cis*-**7a** …………………………….…………. 39
PM6 Cartesian Coordinates & info for *trans*-**7a** …………………........…………….…… 40
PM6 Cartesian Coordinates & info for *cis*-**7b** ……………………………….…………… 41
PM6 Cartesian Coordinates & info for *trans*-**7b** …………………........…………….…… 42
PM6 Cartesian Coordinates & info for cis-**9a** ………………………………......…… 44
PM6 Cartesian Coordinates & info for *trans*-**9a** …………………………….……….... 45
PM6 Cartesian Coordinates & info for *cis*-**9b** ……………………………….……….... 46
PM6 Cartesian Coordinates & info for *trans*-**9b** ……………………………………..…. 47
PM6 Cartesian Coordinates & info for *cis*-**10a** ……………………………….……….... 48
PM6 Cartesian Coordinates & info for *cis*-**10b** ………………………………………… 49
PM6 Cartesian Coordinates & info for *cis*-**11a** ………………………….…………… 50
PM6 Cartesian Coordinates & info for *trans*-**11a** …………………………………….…. 52
PM6 Cartesian Coordinates & info for cis-**11b** ……………………………….…………. 53
PM6 Cartesian Coordinates & info for *trans*-**11b** ………………………….………… 54
PM6 Cartesian Coordinates & info for cis-**19a** ……………………………….………... 55
PM6 Cartesian Coordinates & info for *trans*-**19a** ……………………….………... 56
PM6 Cartesian Coordinates & info for *cis*-**19b** ………………………………………... 57
PM6 Cartesian Coordinates & info for *trans*-**19b** ……………………………..….… 59
PM6 Cartesian Coordinates & info for *cis*-**20a** …………………………………….. 60
PM6 Cartesian Coordinates & info for *trans*-**20a** ………………………….……….. 61
PM6 Cartesian Coordinates & info for *cis*-**20b** ……….……………….……….. 63
PM6 Cartesian Coordinates & info for *trans*-**20b** ……………………….……….... 64
PM6 Cartesian Coordinates & info for **I** ……………………..................………. 64
PM6Cartesian Coordinates & info for **II** ……………………..................……….. 65
PM6 Cartesian Coordinates & info for **III** …………………..................………....... 67
PM6 Cartesian Coordinates & info for **IV** …………………….................…….. 68

^{1}H NMR for (±)-(*E*,*R*,*R*)-3-[2-nitro-1-phenyleth-1-oxy]hex-4-en-1-yne **(6a)**

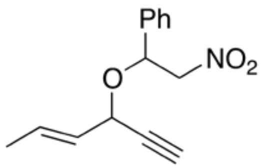

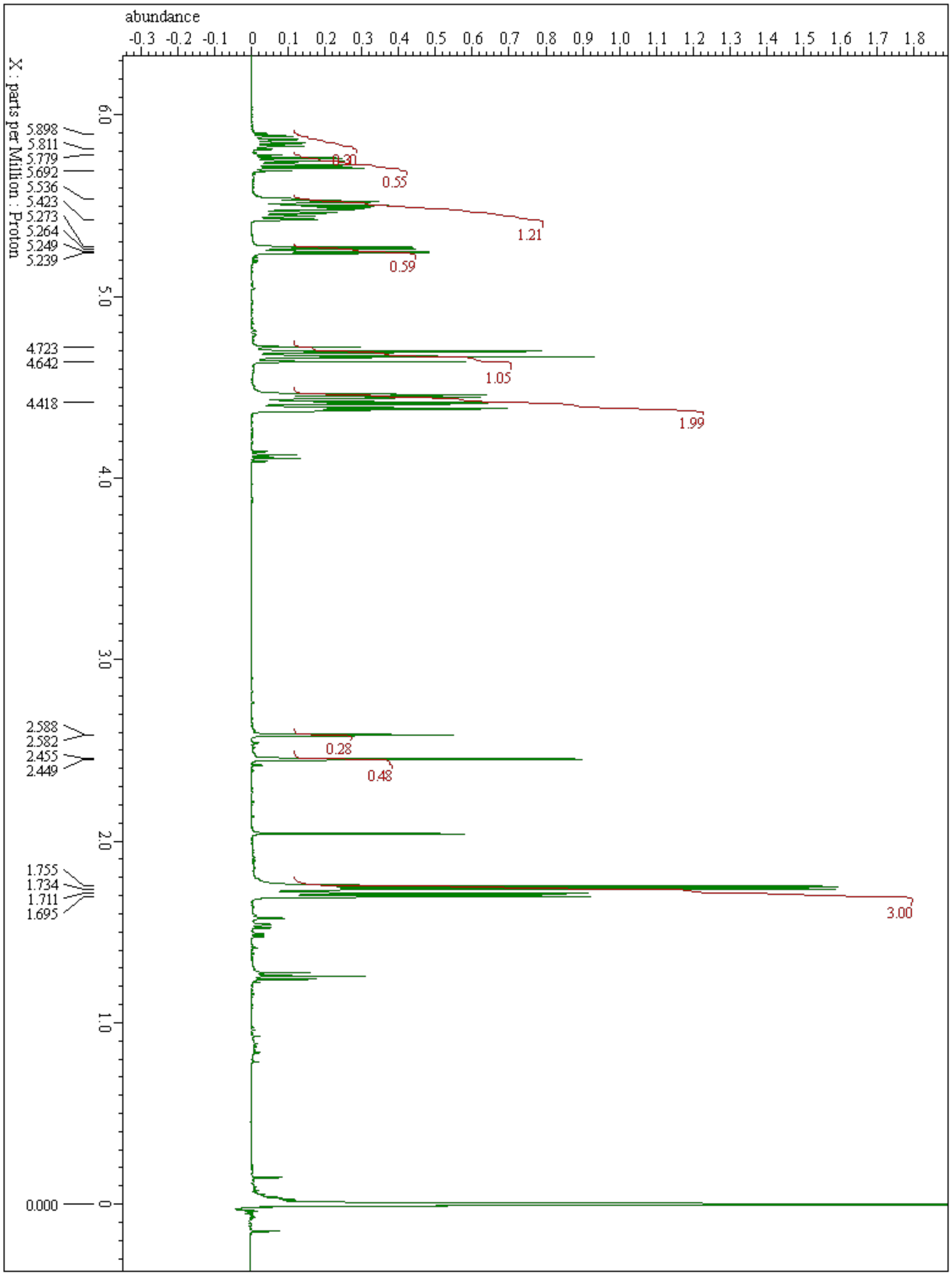

$^{13}C\{^{1}H\}$ NMR for (±)-(*E*,*R*,*R*)-3-[2-nitro-1-phenyleth-1-oxy]hex-4-en-1-yne (**6a)**

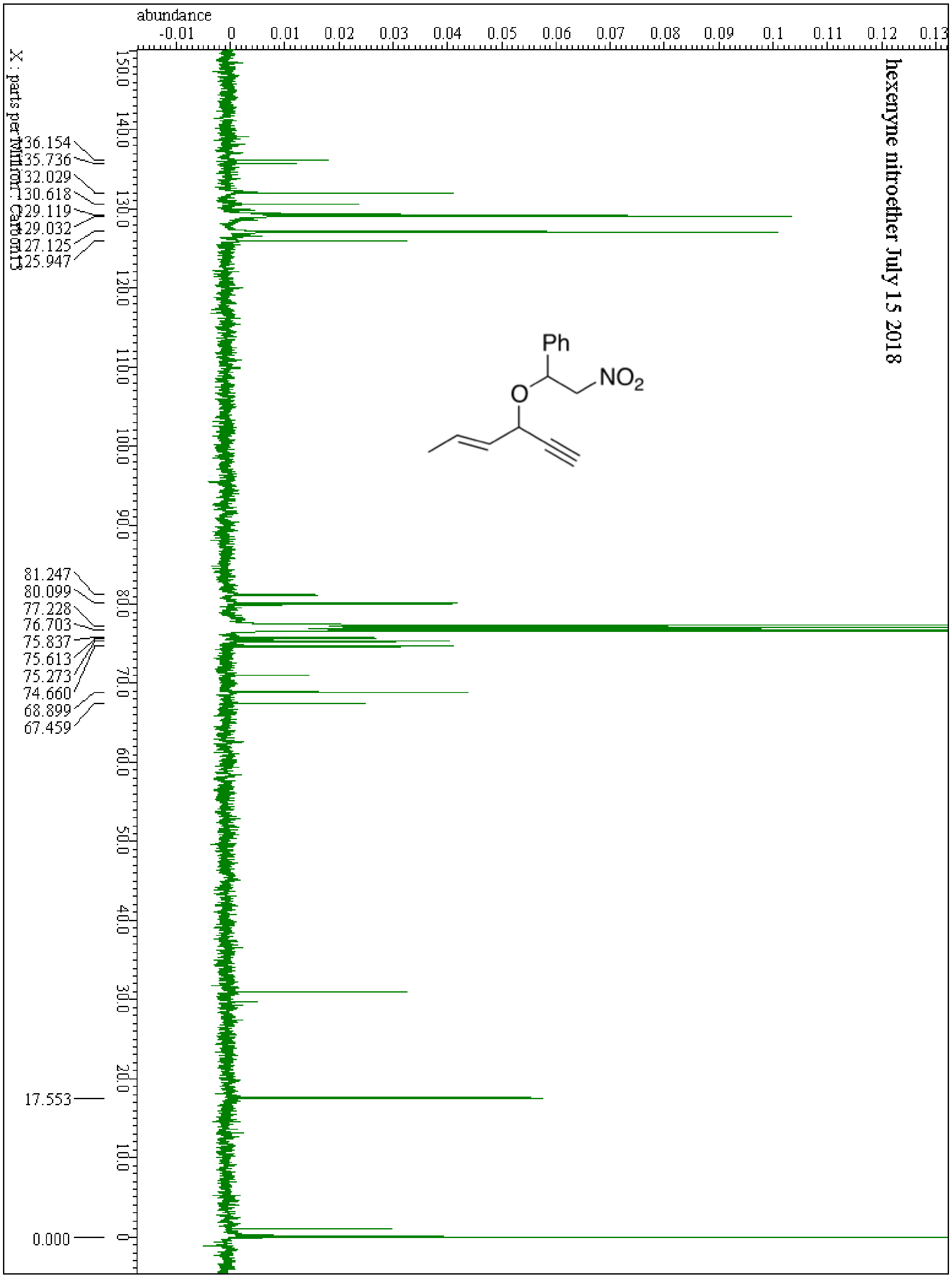

HMQC for (±)-(*E*,*R*,*R*)-3-[2-nitro-1-phenyleth-1-oxy]hex-4-en-1-yne (**6a**)

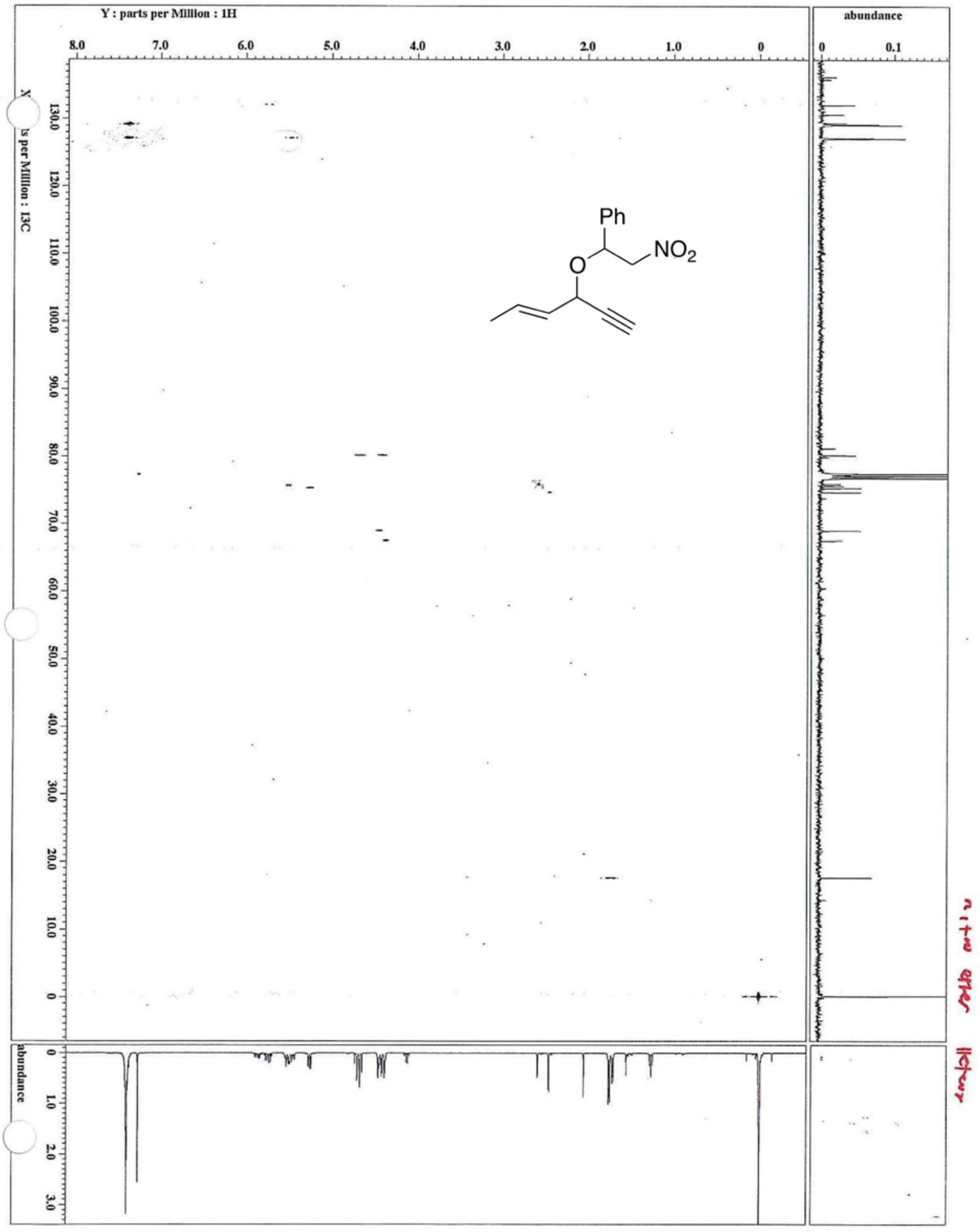

COSY for (±)-(*E*,*R*,*R*)-3-[2-nitro-1-phenyleth-1-oxy]hex-4-en-1-yne (**6a)**

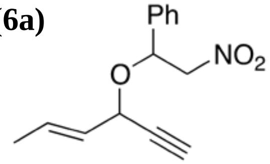

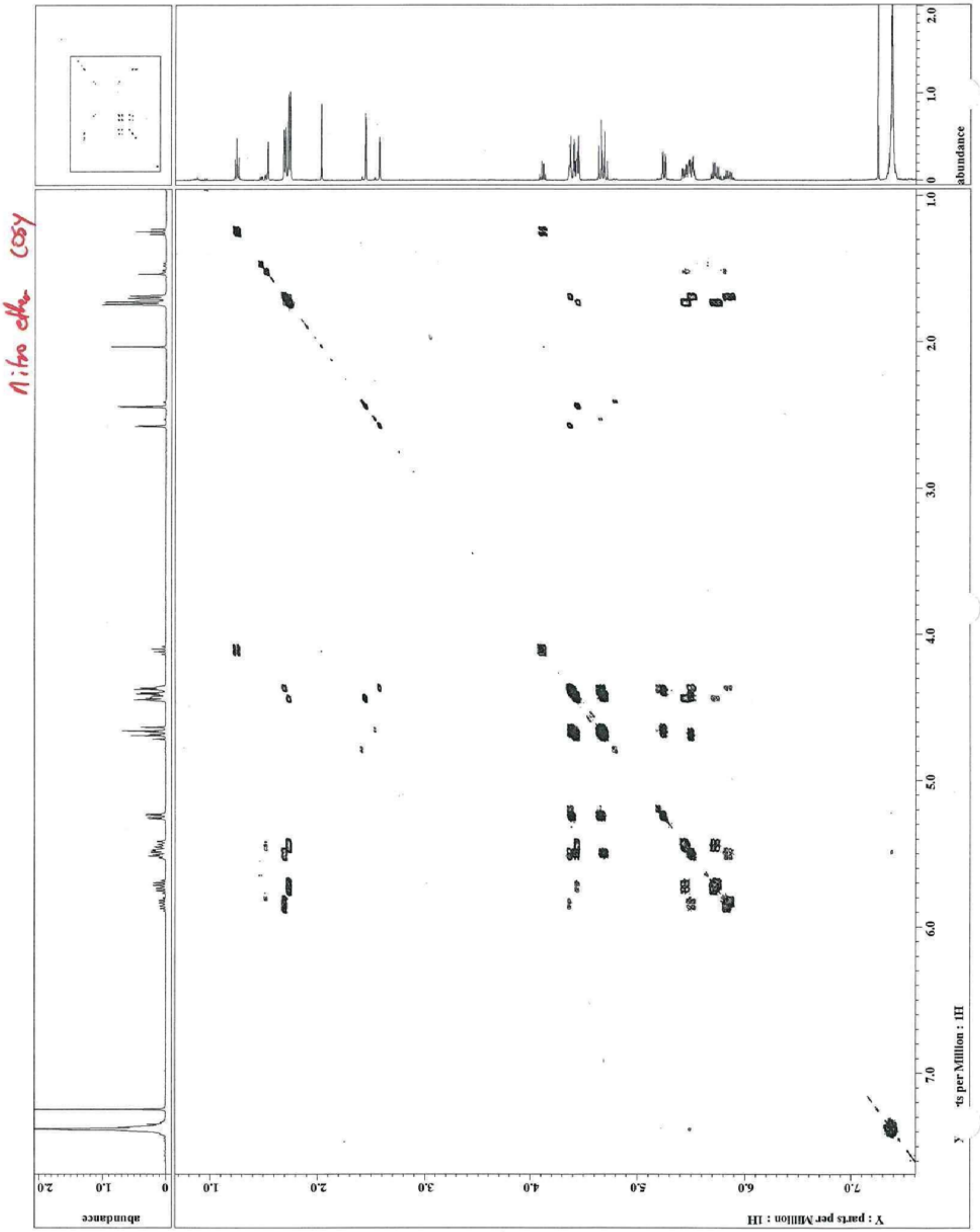

DEPT for (±)-(*E*,*R*,*R*)-3-[2-nitro-1-phenyleth-1-oxy]hex-4-en-1-yne (**6a)**

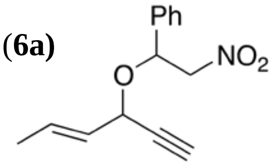

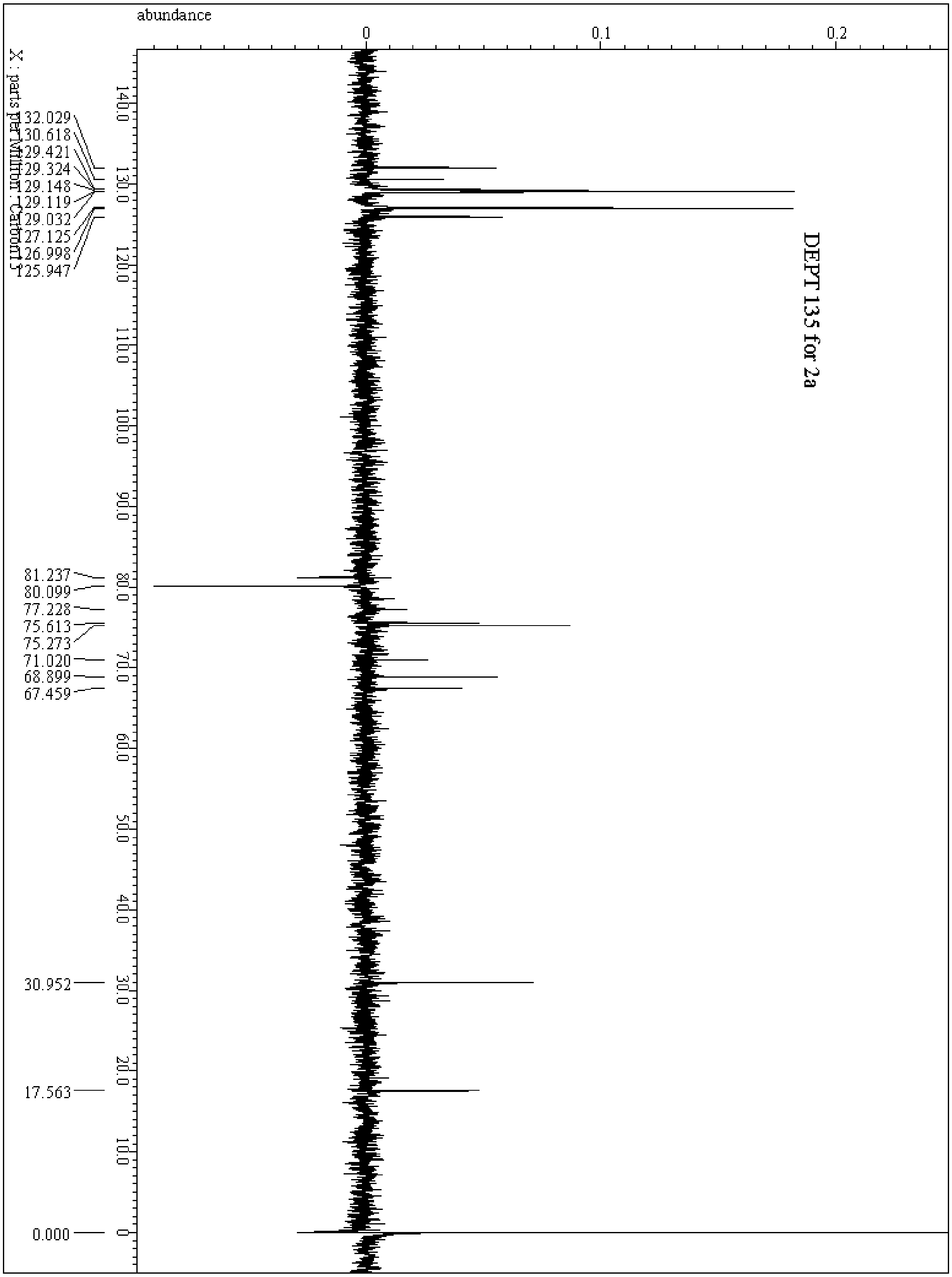

^{1}H NMR for (±)-(*E*,*R*,*R*)-3-[2-nitro-1-phenyleth-1-oxy]hept-2-en-4-yne (**6b)**

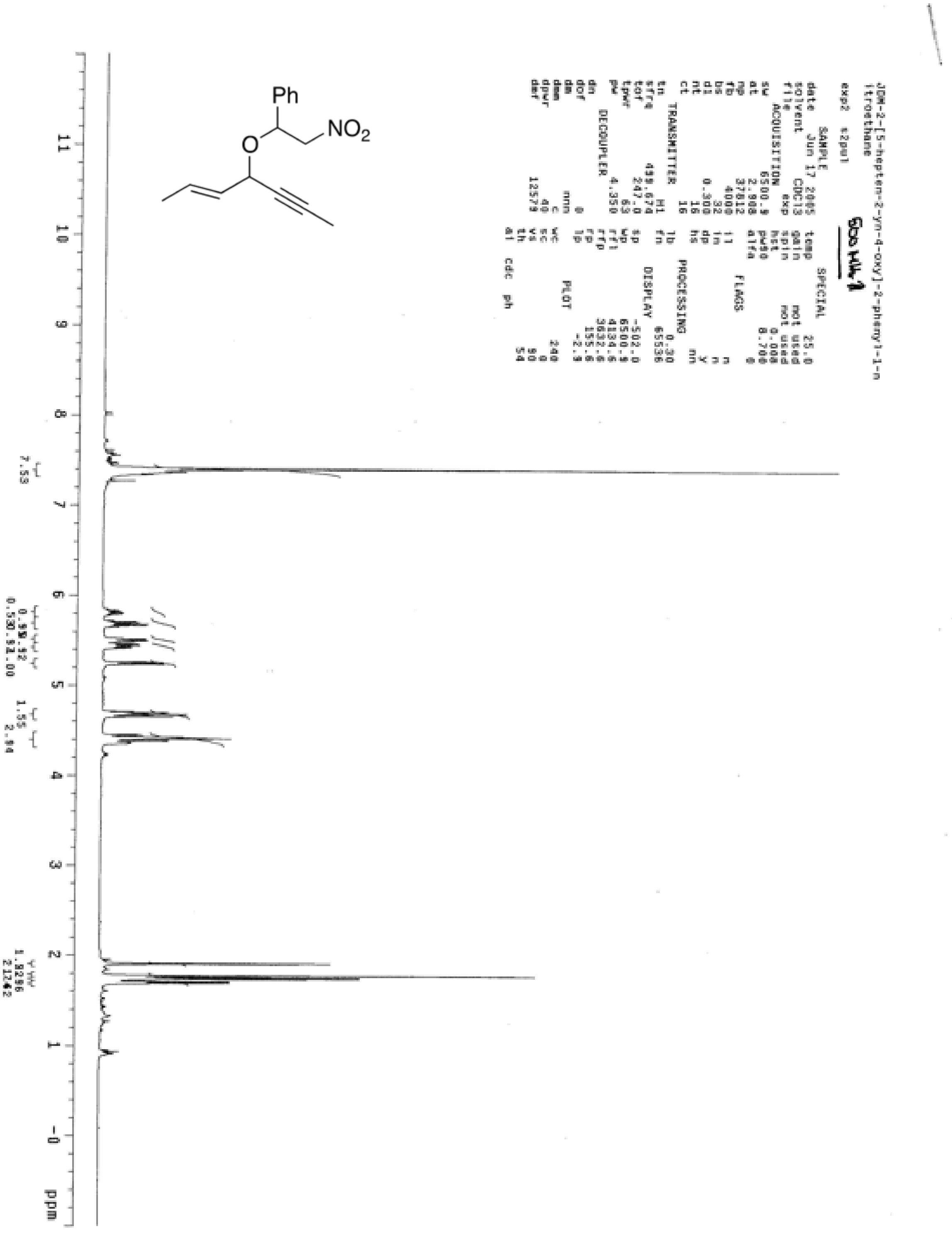

$^{13}C\{^{1}H\}$ NMR for (±)-(*E*,*R*,*R*)-3-[2-nitro-1-phenyleth-1-oxy]hept-2-en-4-yne (**6b)**

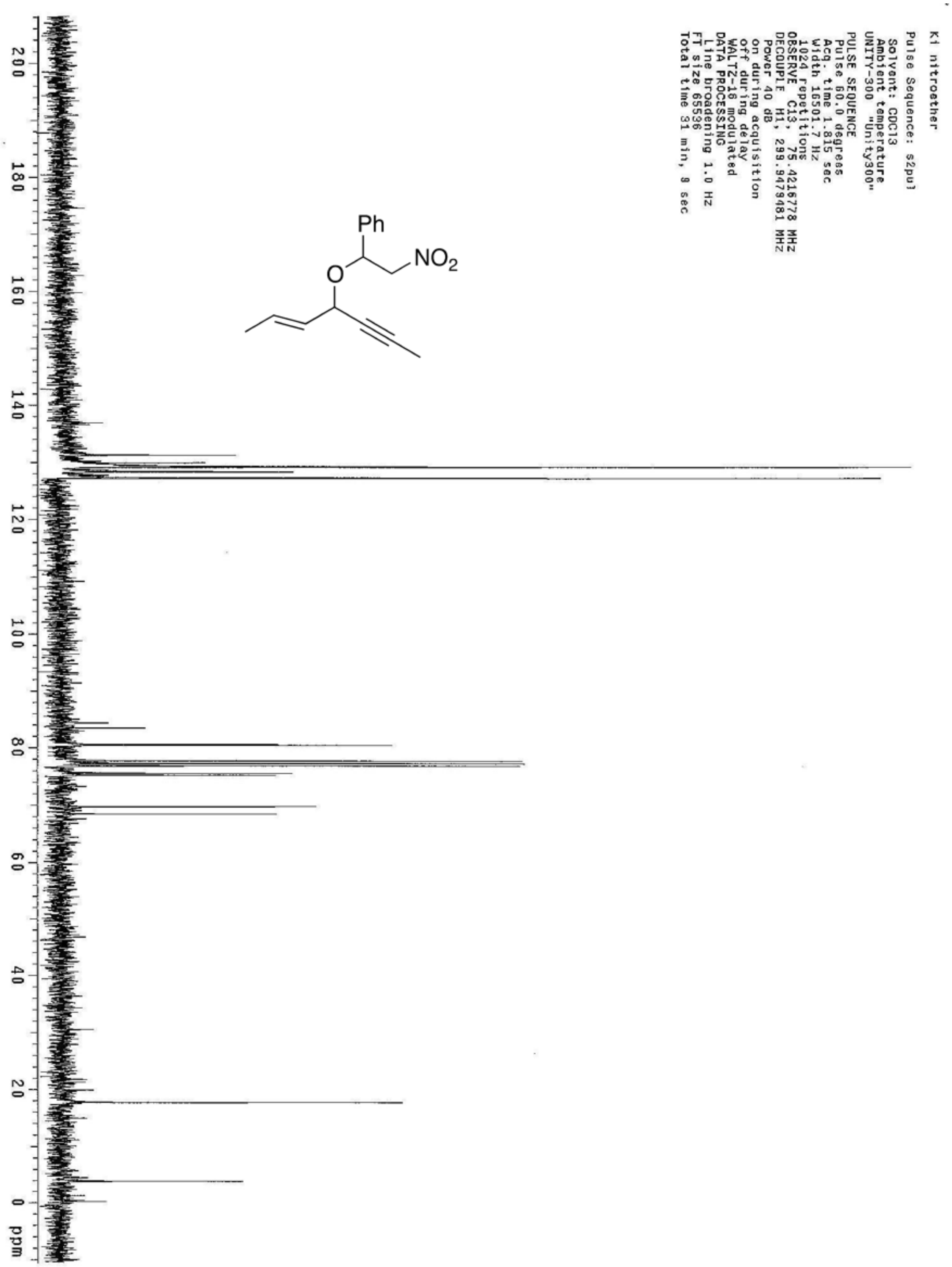

HETCOR for (±)-(E,R,R)-3-[2-nitro-1-phenyleth-1-oxy]hept-2-en-4-yne (**6b)**

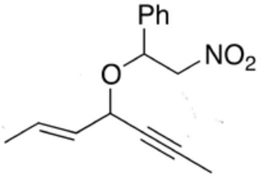

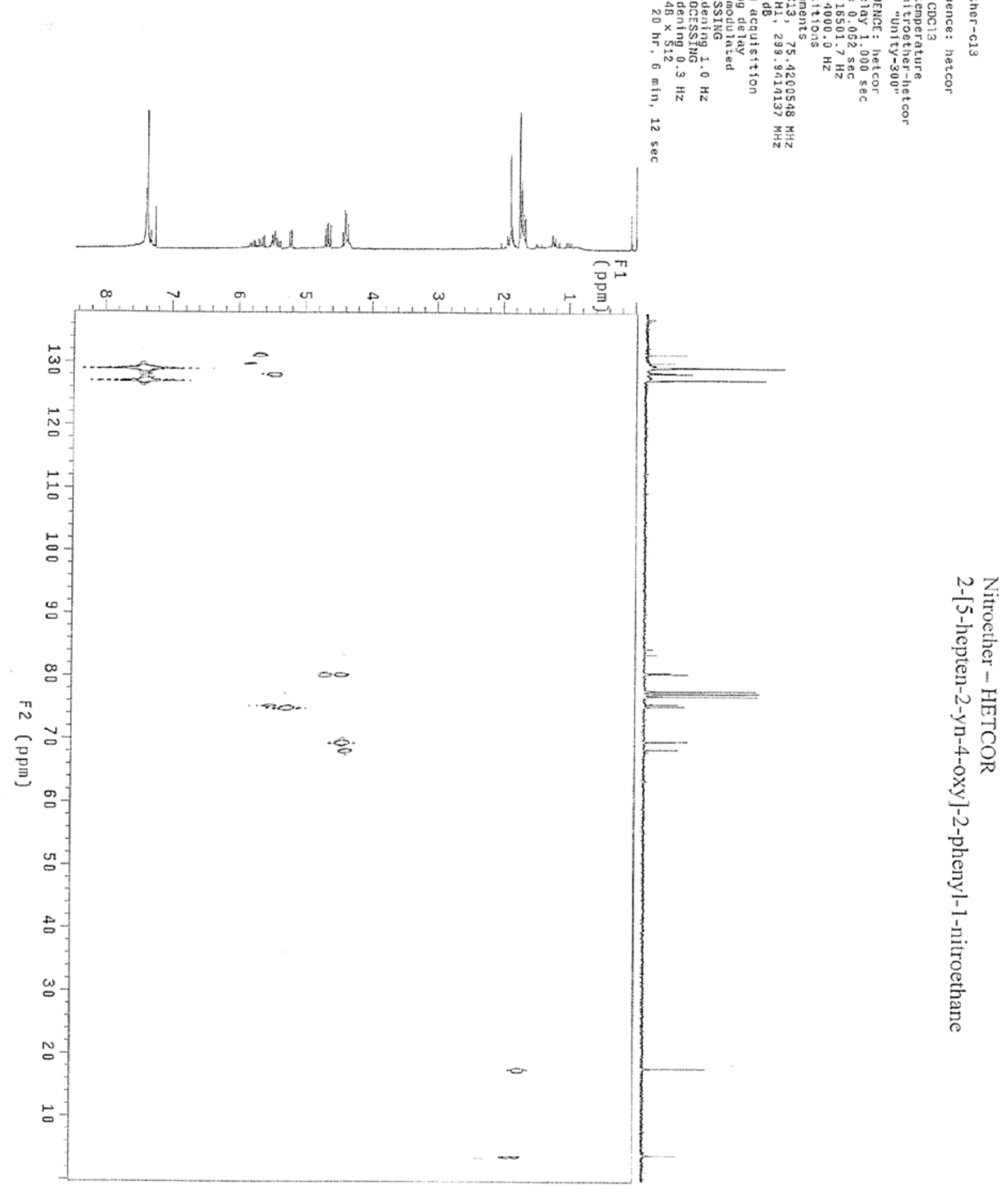

COSY for (±)-(*E*,*R*,*R*))-3-[2-nitro-1-phenyleth-1-oxy]hept-2-en-4-yne (**6b)**

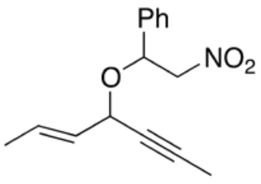

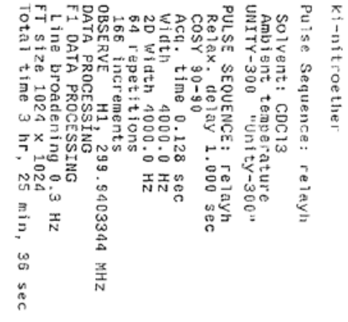

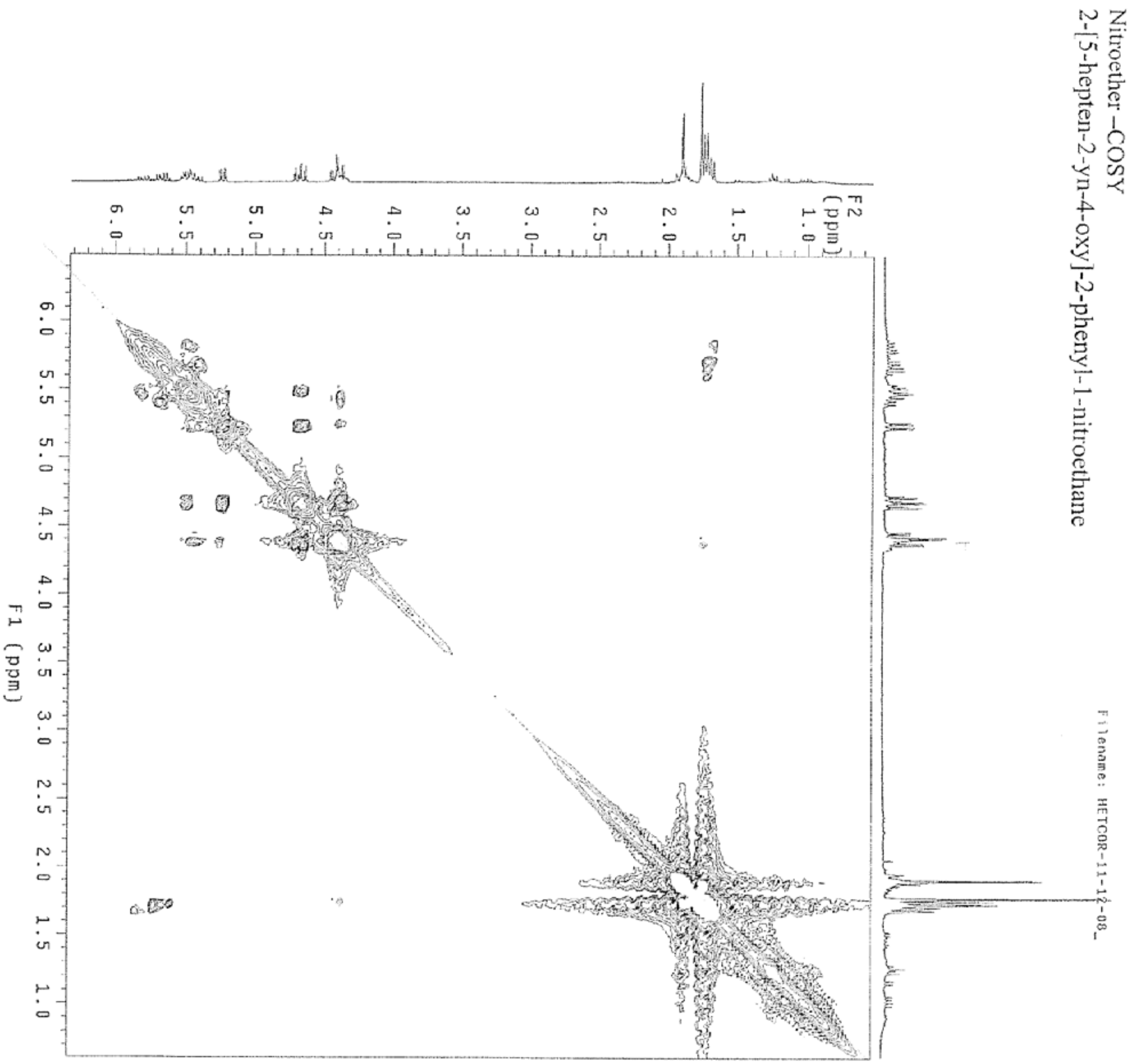

DEPT for (±)-(*E*,*R*,*R*)-3-[2-nitro-1-phenyleth-1-oxy]hept-2-en-4-yne (**6b)**

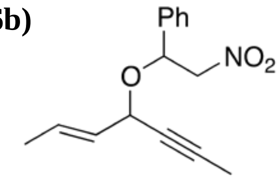

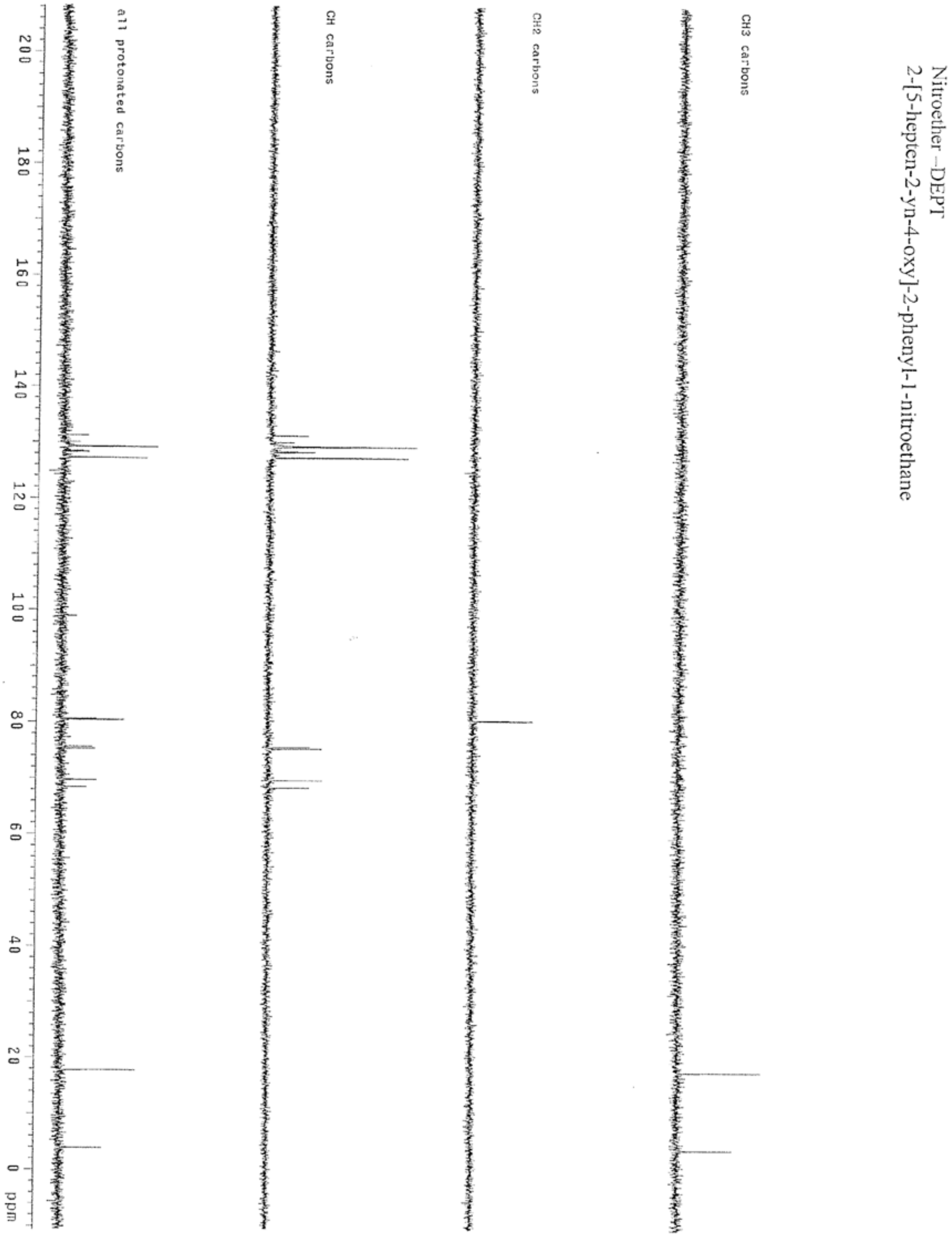

^{1}H NMR for (±)-(*3S,3aS,4R,6R*)-3,3a-dihydro-3-methyl-6-phenyl-4-[eth-1-ynyl]-*4H,6H*-furo[3,4-c]isoxazole. (**10a)** I , major diastereomer

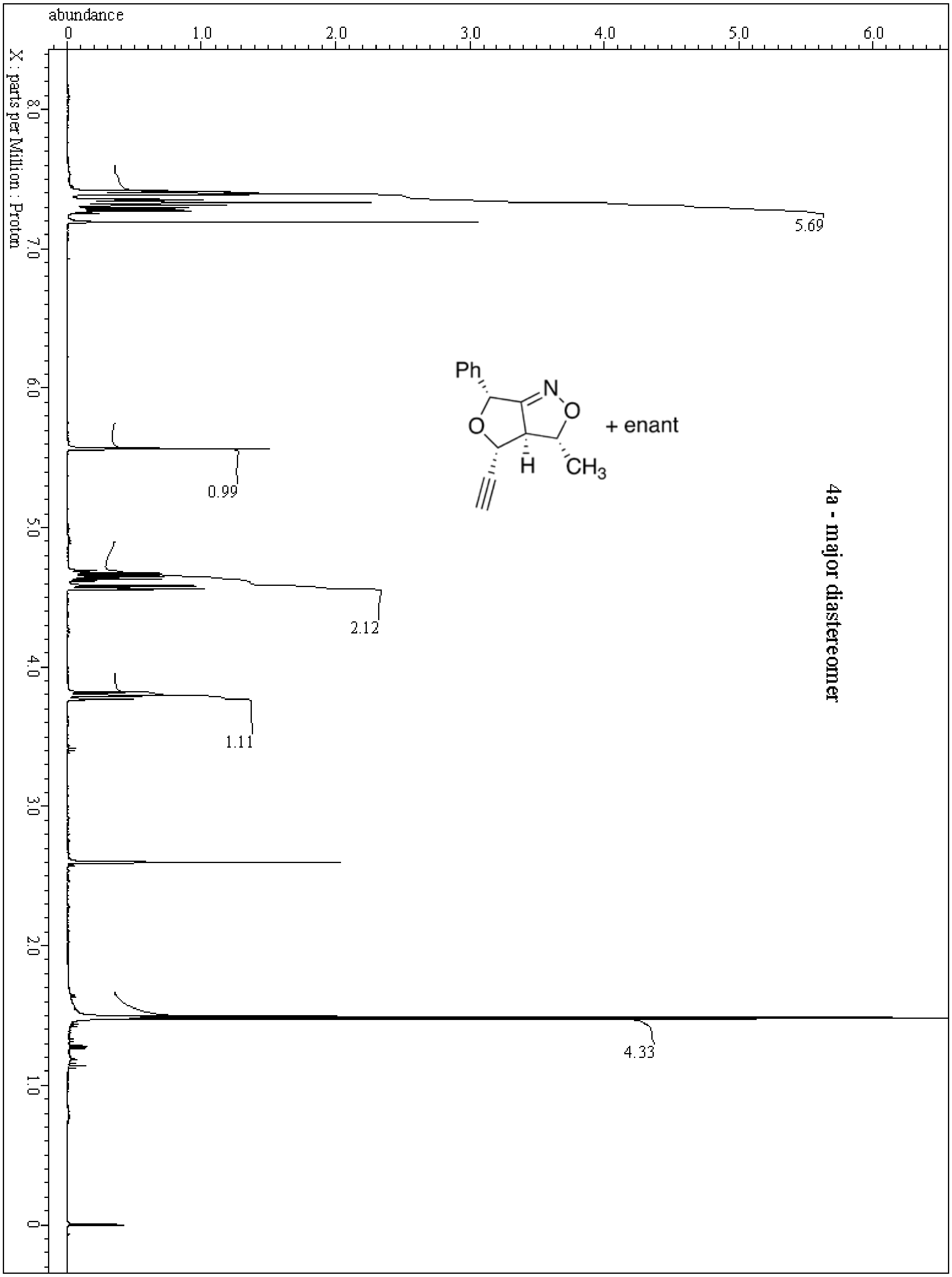

^{1}H NMR for (±)-(*3S*,*3aS*,*4S*,*6R*)-3,3a-dihydro-3-methyl-6-phenyl-4-[eth-1-ynyl]-*4H*,*6H*-furo[3,4-c]isoxazole. (**10a)** II , minor diastereomer

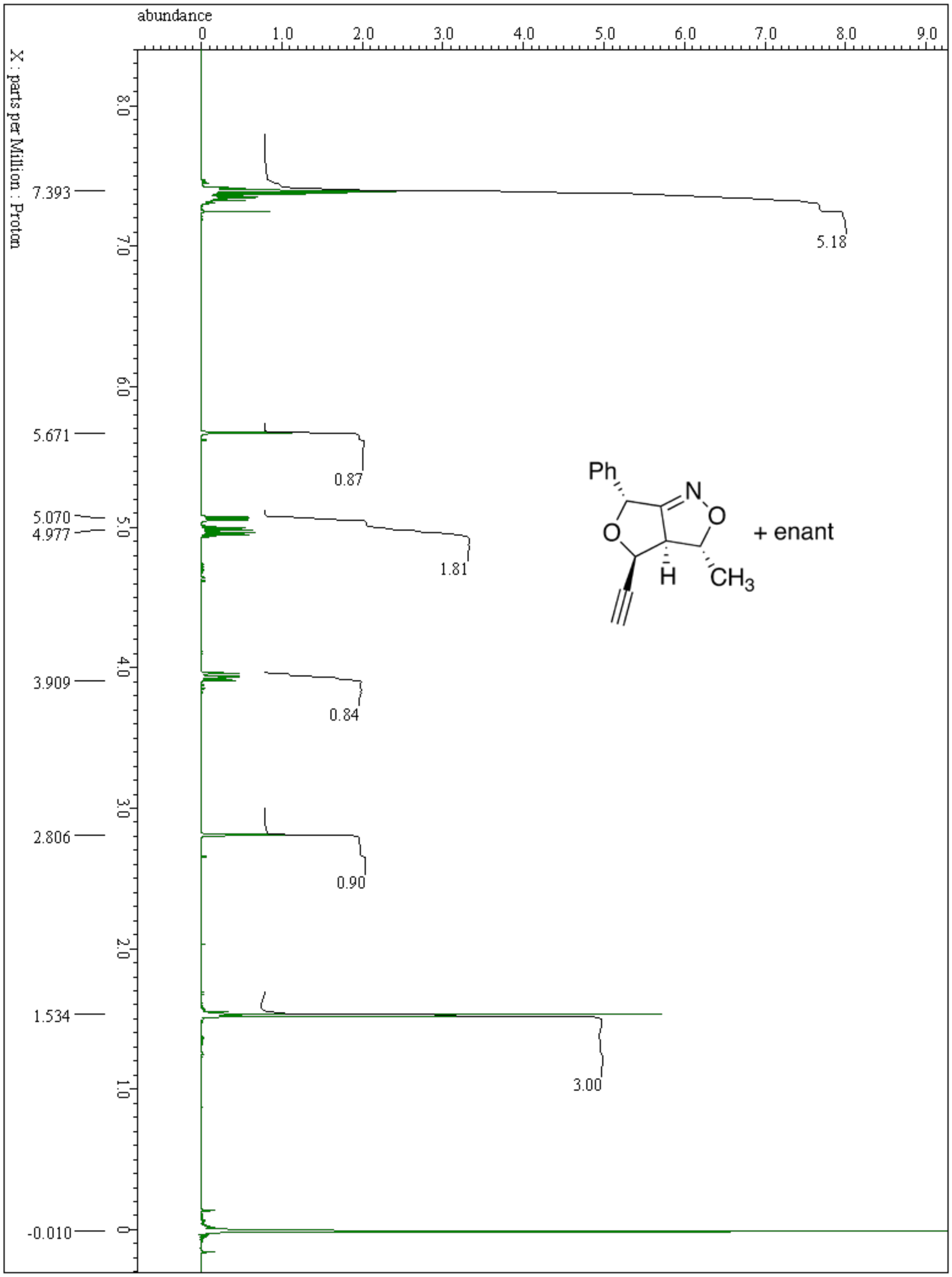

$^{13}C\{^{1}H\}$ NMR for (±)-(*3S*,*3aS*,*4R*,*6R*)-3,3a-dihydro-3-methyl-6-phenyl-4-[eth-1-ynyl]-*4H*,*6H*-furo[3,4-c]isoxazole. (**10a**) I, major diastereomer

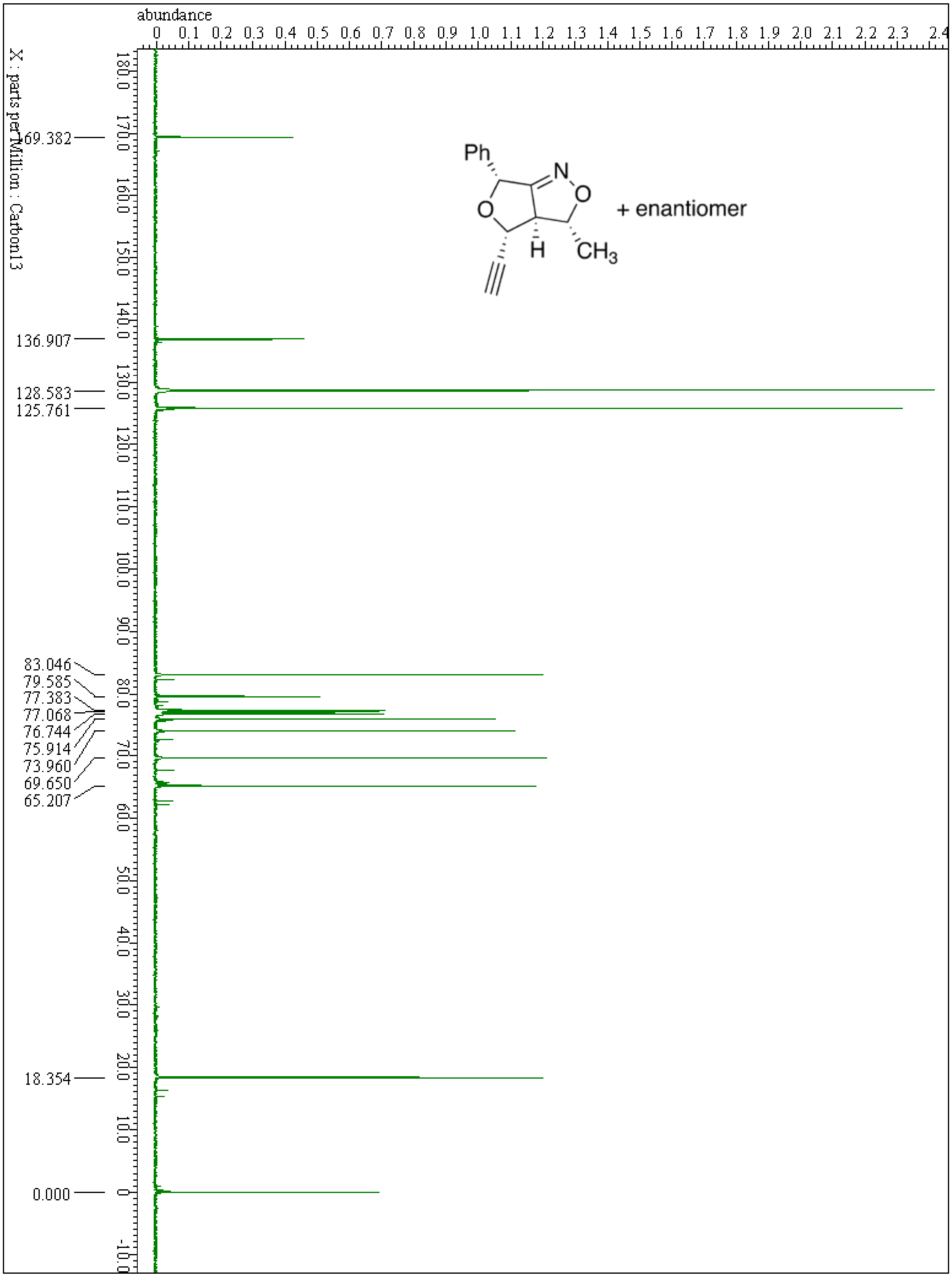

$^{13}C\{^1H\}$ NMR for (±)-(*3S,3aS,4S,6R*)-3,3a-dihydro-3-methyl-6-phenyl-4-[eth-1-ynyl]-*4H,6H*-furo[3,4-c]isoxazole. (**10a)** II , minor diastereomer

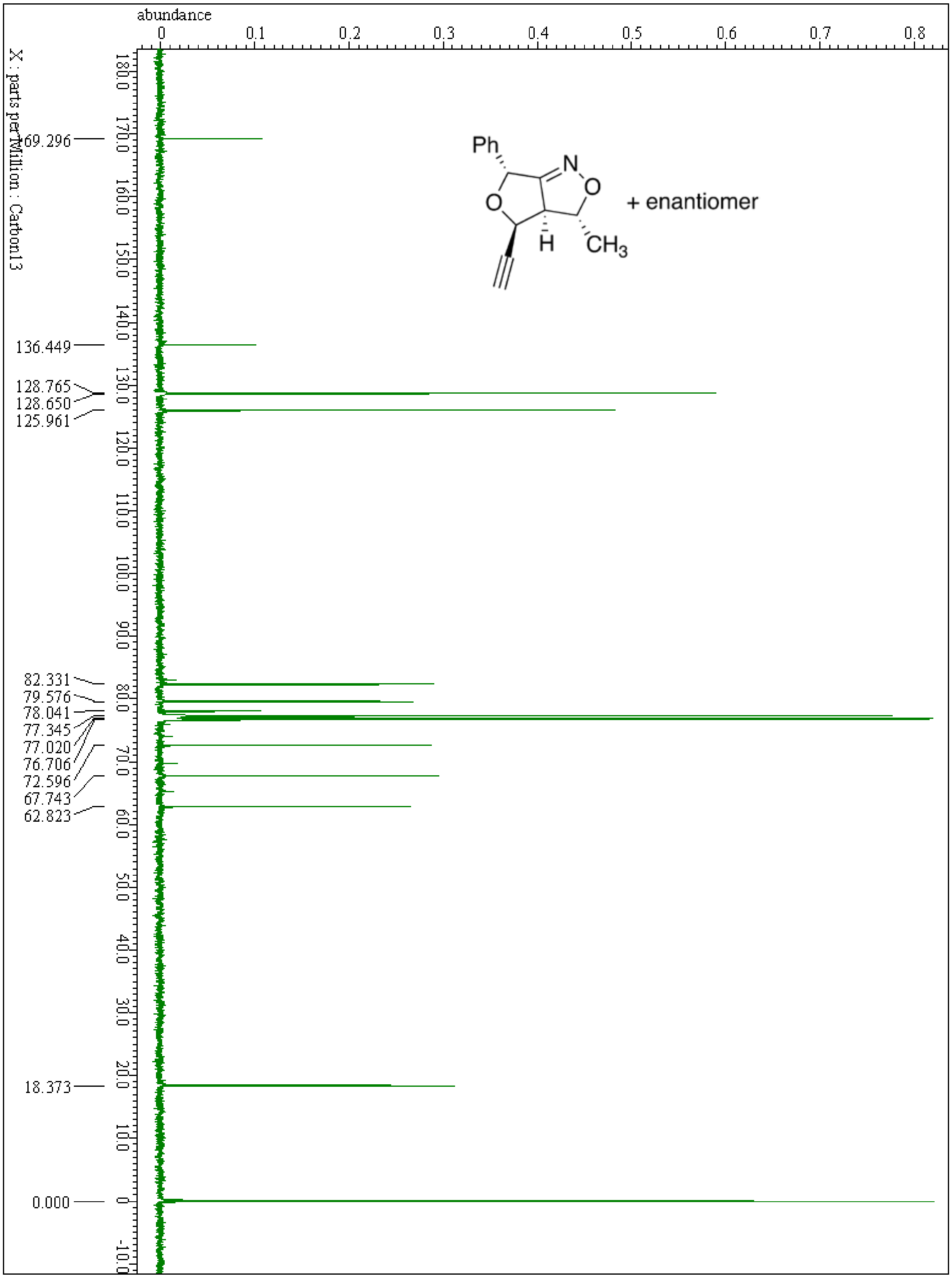

HMQC for (±)-(*3S*,*3aS*,*4R*,*6R*)-3,3a-dihydro-3-methyl-6-phenyl-4-[eth-1-ynyl]-*4H*,*6H*-furo[3,4-c]isoxazole. (**10a)** I , major diastereomer

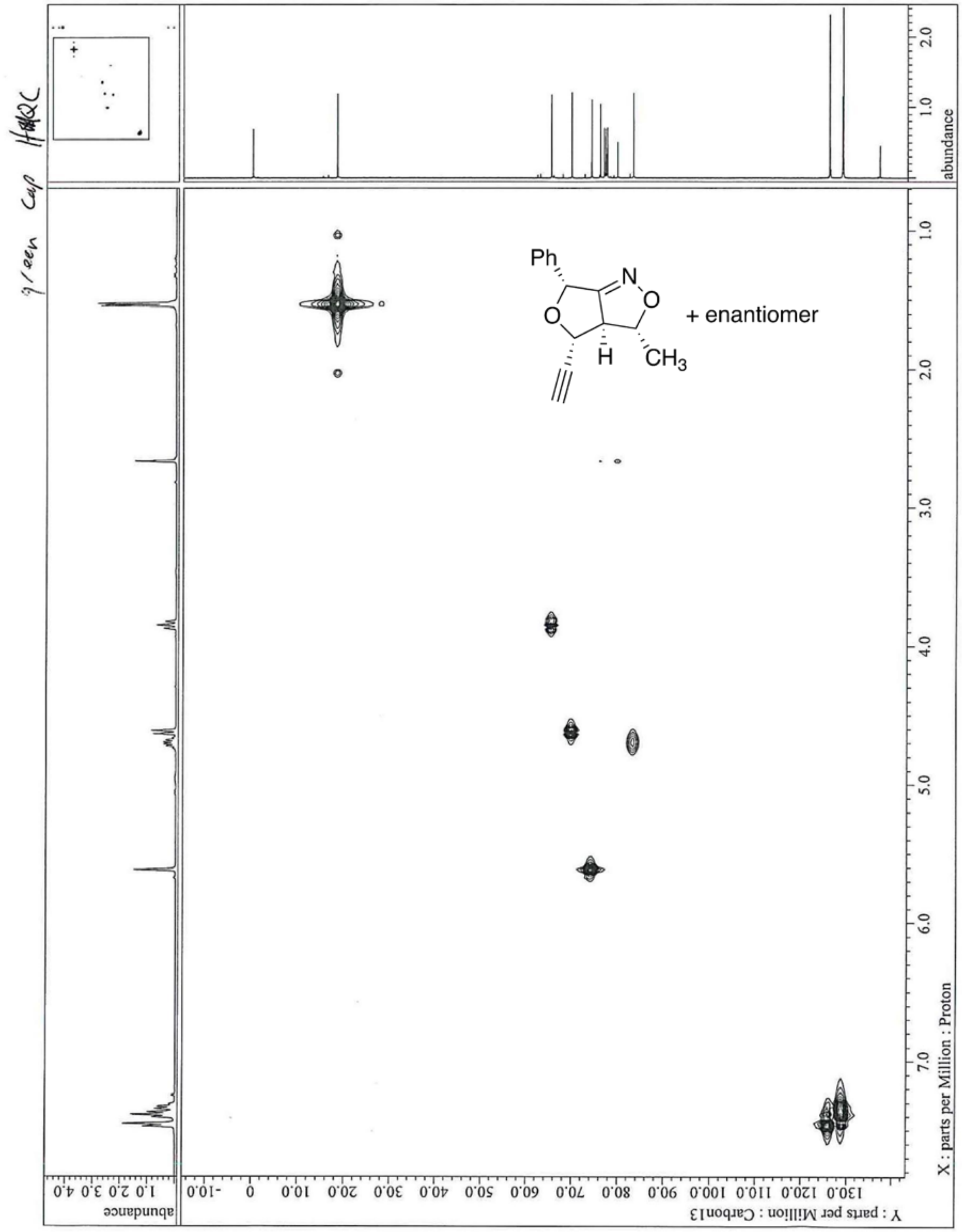

HMQC for (±)-(*3S,3aS,4S,6R*)-3,3a-dihydro-3-methyl-6-phenyl-4-[eth-1-ynyl]-*4H,6H*-furo[3,4-c]isoxazole. (**10a)** II , minor diastereomer

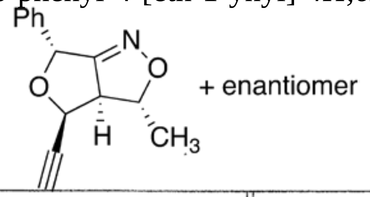

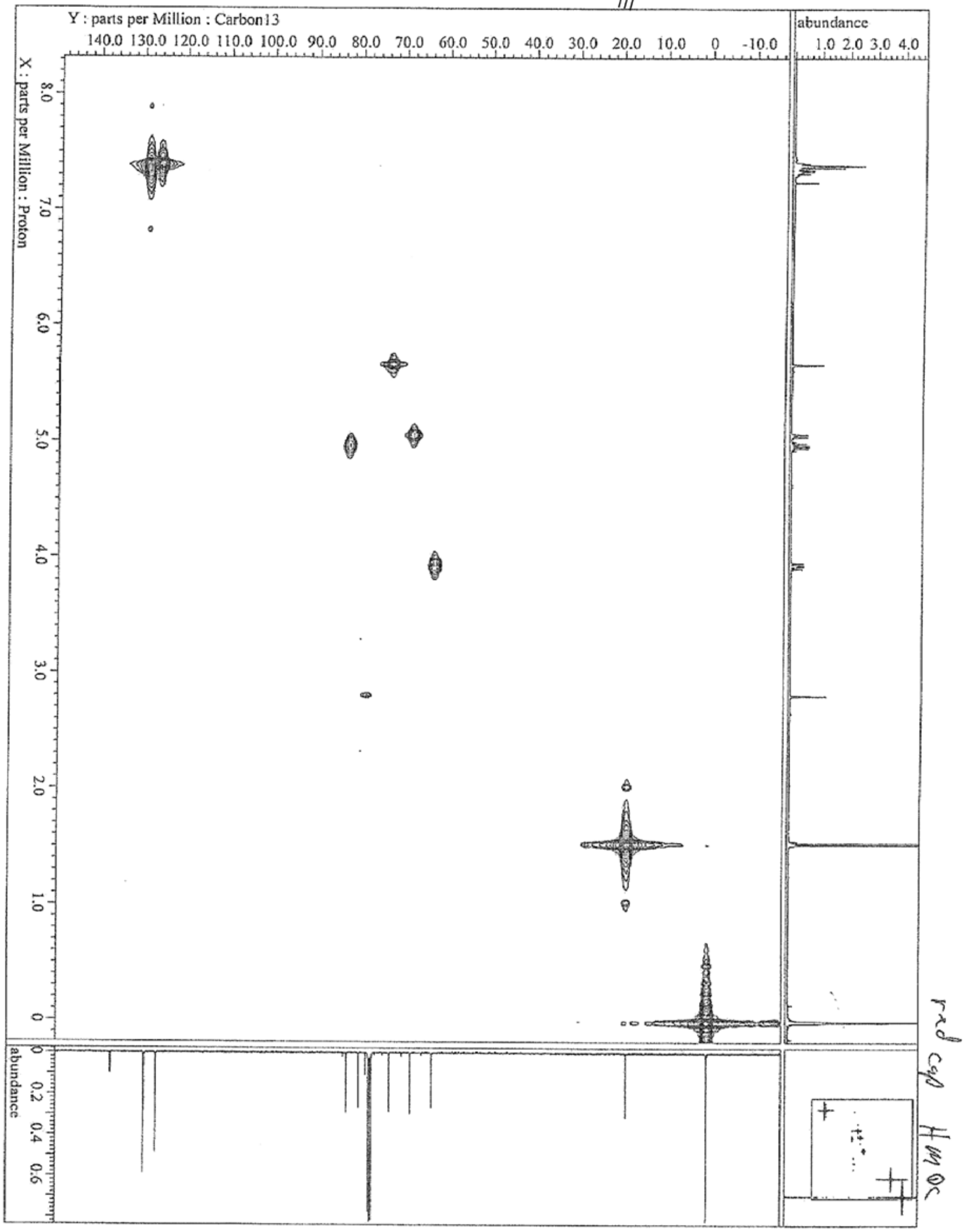

COSY for (±)-(*3S,3aS,4R,6R*)-3,3a-dihydro-3-methyl-6-phenyl-4-[eth-1-ynyl]-*4H,6H*-furo[3,4-c]isoxazole. (**10a)** I, major diastereomer

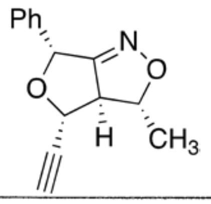

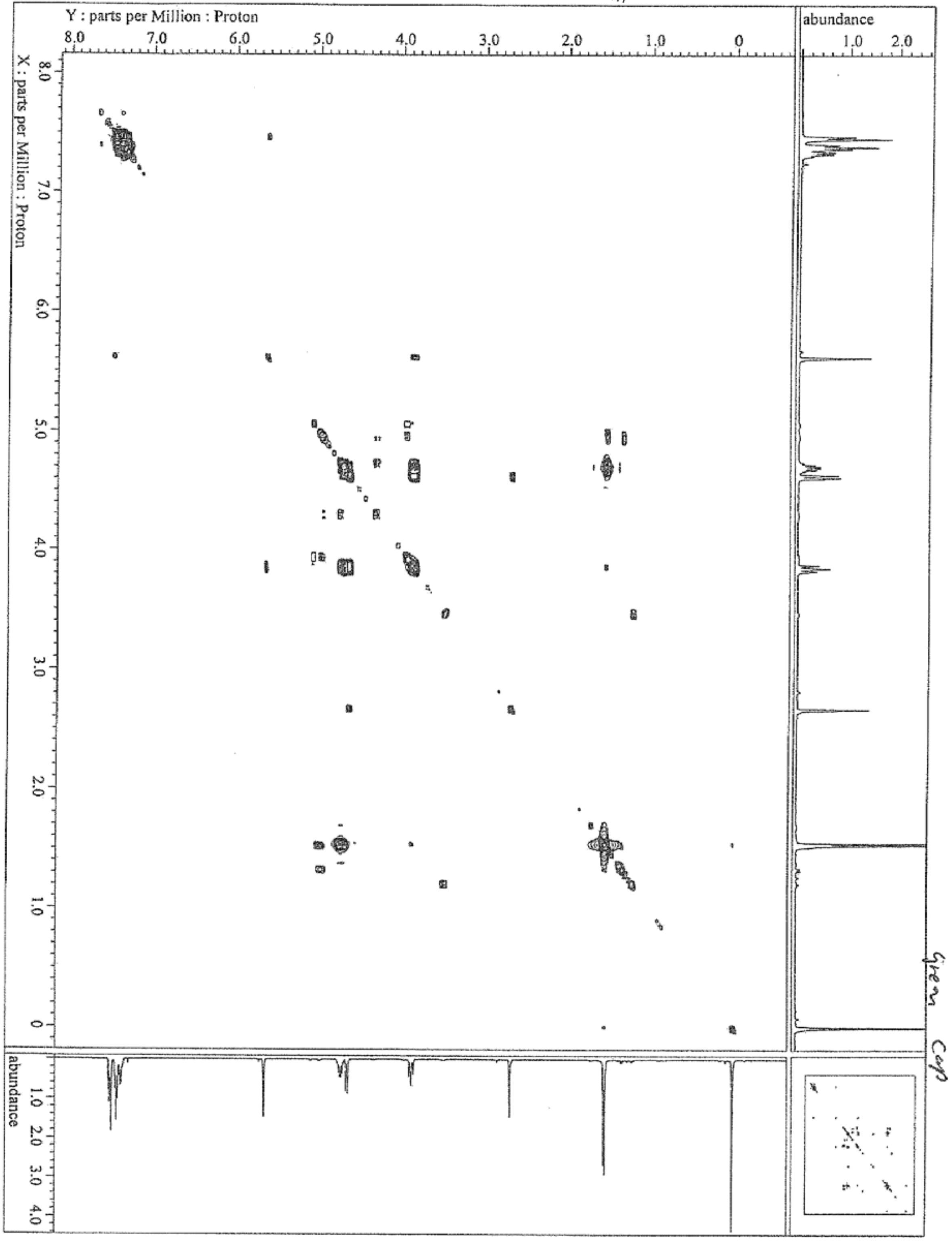

COSY for (±)-(*3S*,*3aS*,*4S*,*6R*)-3,3a-dihydro-3-methyl-6-phenyl-4-[eth-1-ynyl]-*4H*,*6H*-furo[3,4-c]isoxazole. (**10a)** II, minor diastereomer

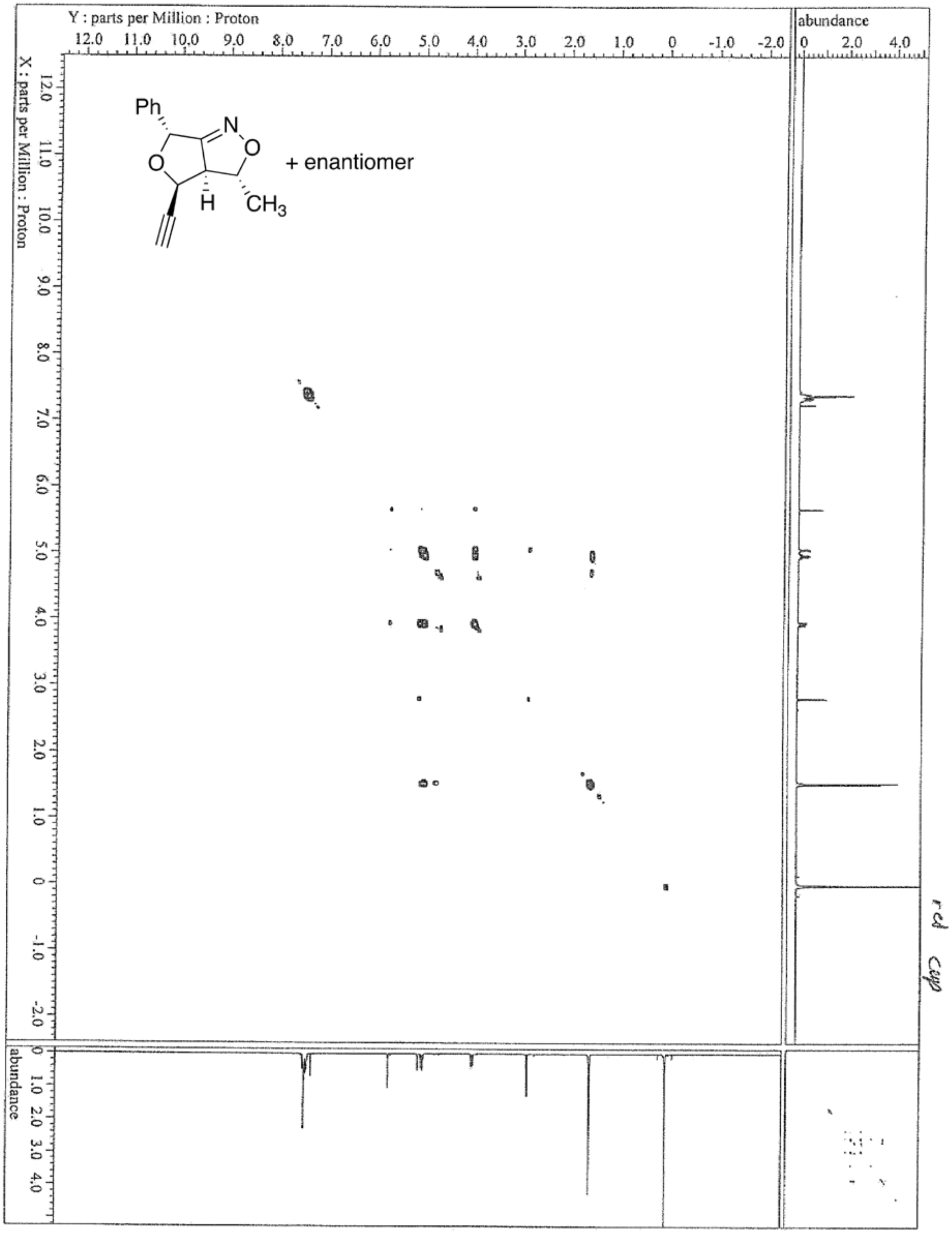

DEPT for (±)-(*3S,3aS,4R/S,6R*)-(3,3a-dihydro-3-methyl-6-phenyl-4-[eth-1-ynyl]-*4H,6H*-furo[3,4-c]isoxazole (**10a**)

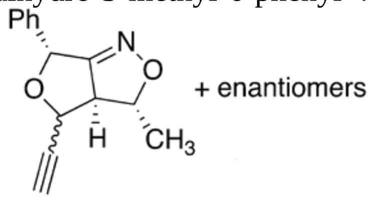

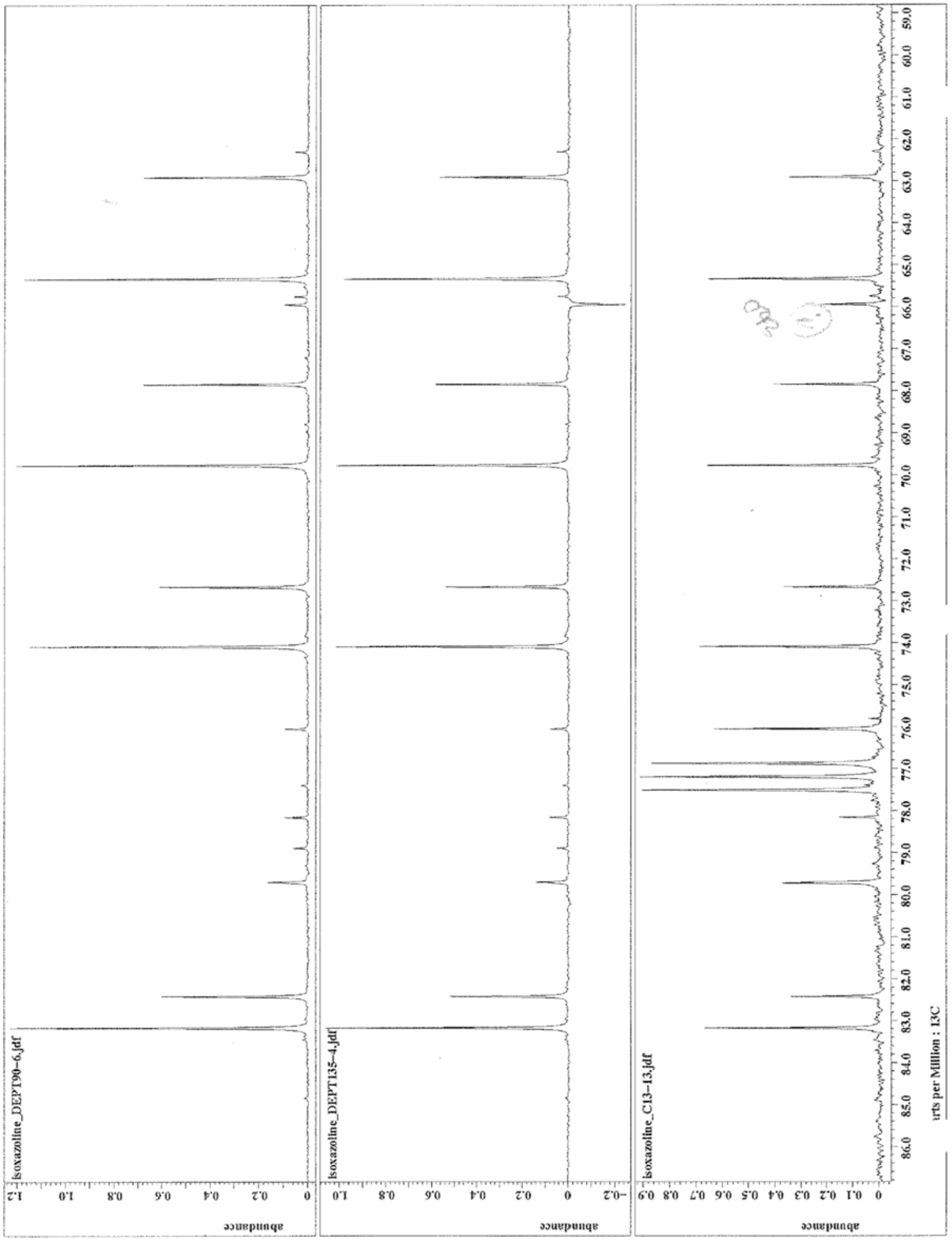

NOE for (±)-(*3S,3aS,4R,6R*)-3,3a-dihydro-3-methyl-6-phenyl-4-[eth-1-ynyl]-*4H,6H*-furo[3,4-c]isoxazole. (**10a)** I, major diastereomer

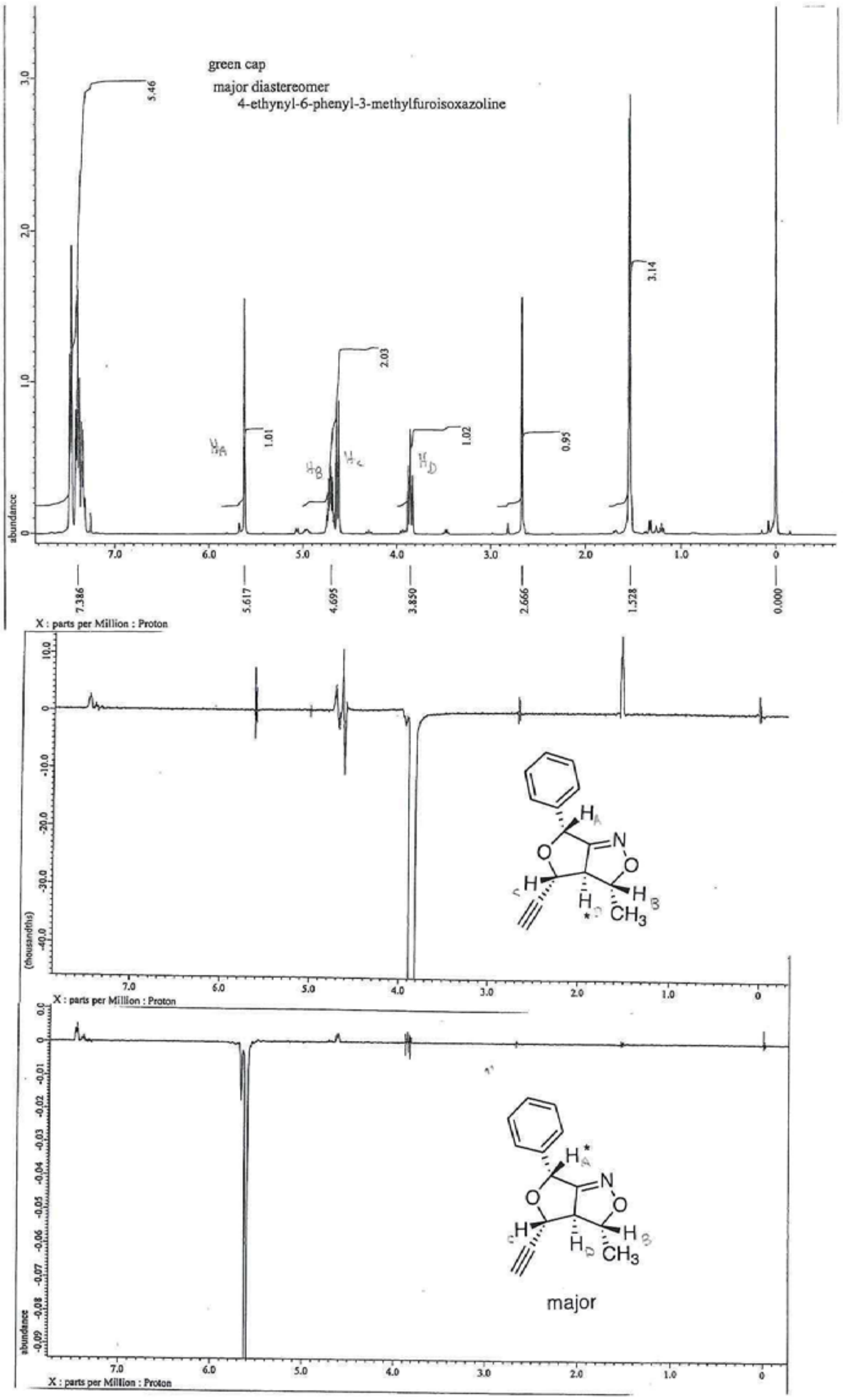

NOE for (±)-(*3R,3aR,4S,6R*)-3,3a-dihydro-3-methyl-6-phenyl-4-[eth-1-ynyl]-*4H,6H*-furo[3,4-c]isoxazole. (**10a)** II , minor diastereomer

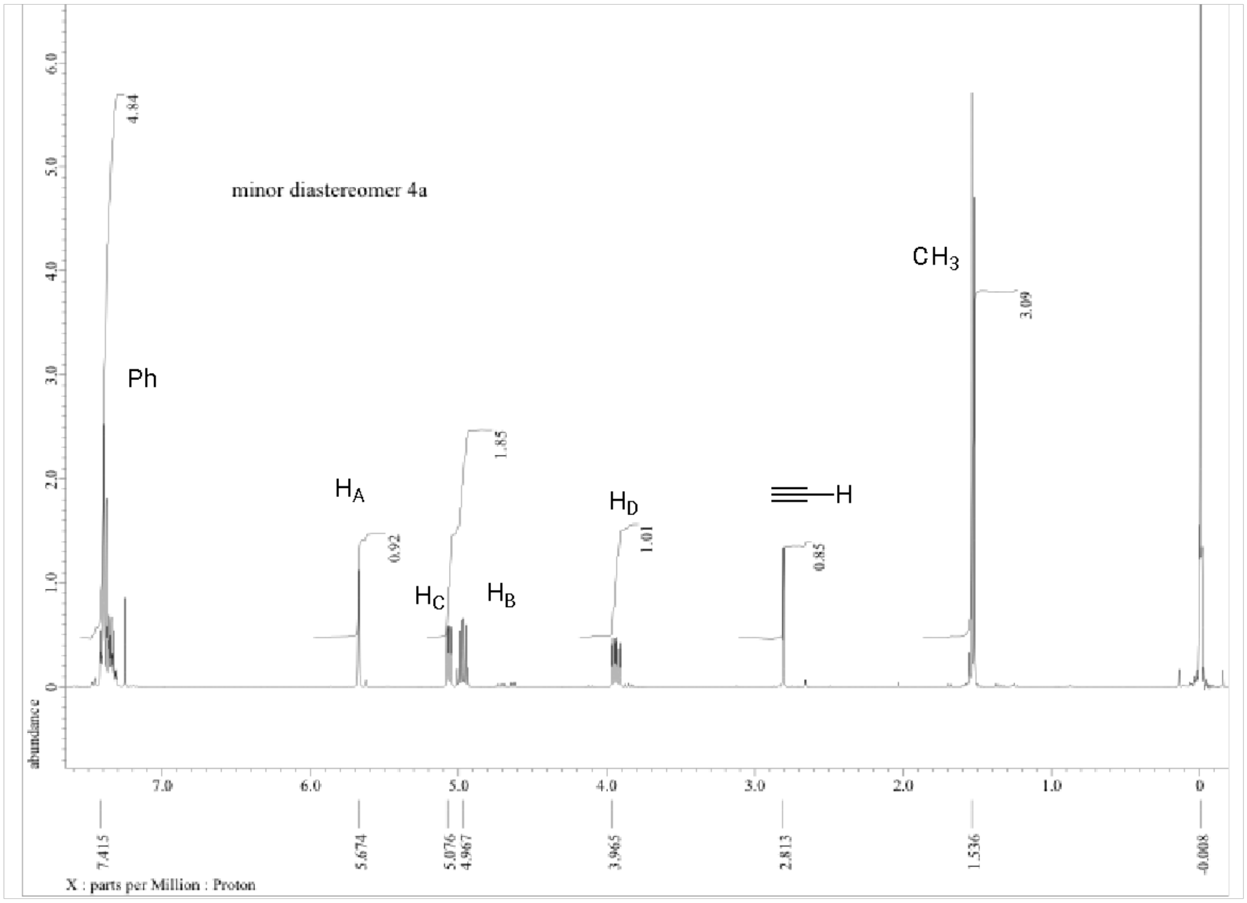

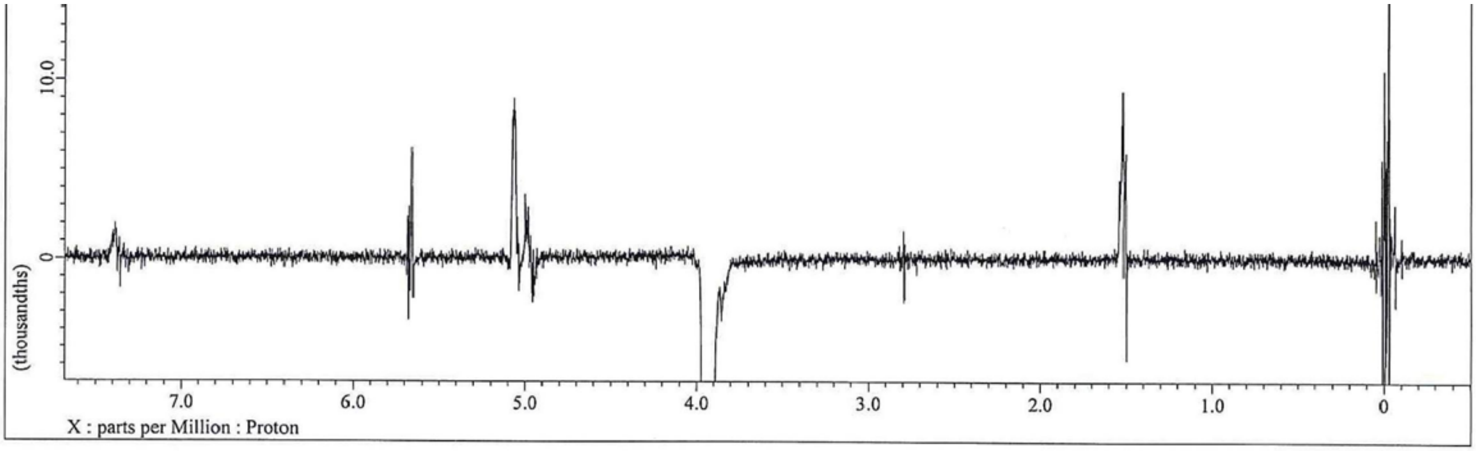

Crude ^{1}H NMR **10a**

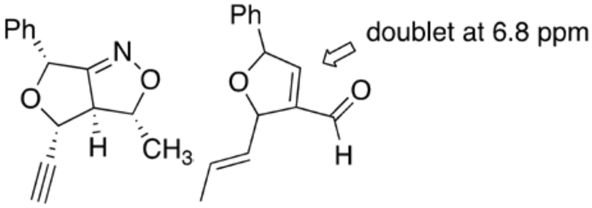

1:1.21 ratio for isoxazoline diastereomers

If 9-10 singlets are carbonyl compounds then 0.24:2.21, ratio 97% isoxazolines: 3% aldehydes
No peaks seen between 6-7 ppm.

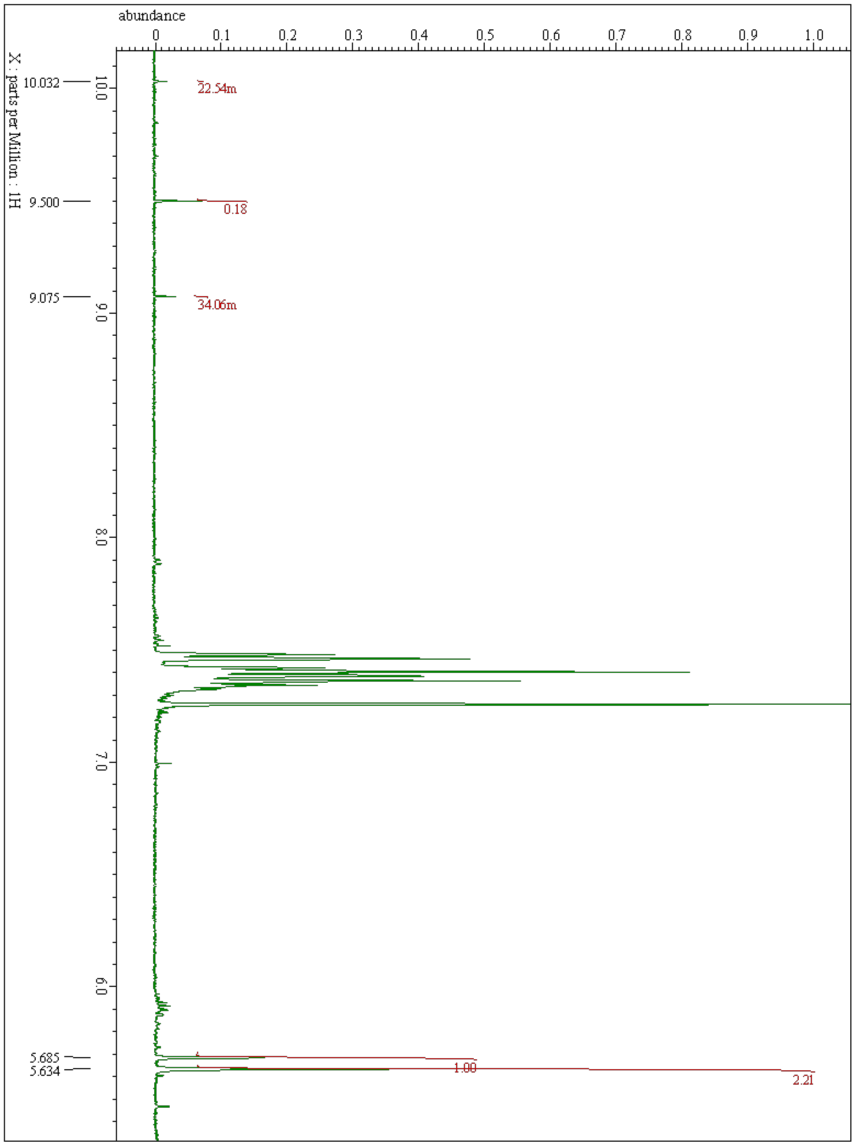

^{1}H NMR for (±)-(*3R*,*3aR*,*4S*,*6R*)-(3,3a-dihydro-3-methyl-6-phenyl-4-[prop-1-ynyl]-*4H*,*6H*-furo[3,4-c]isoxazole (**10b)**

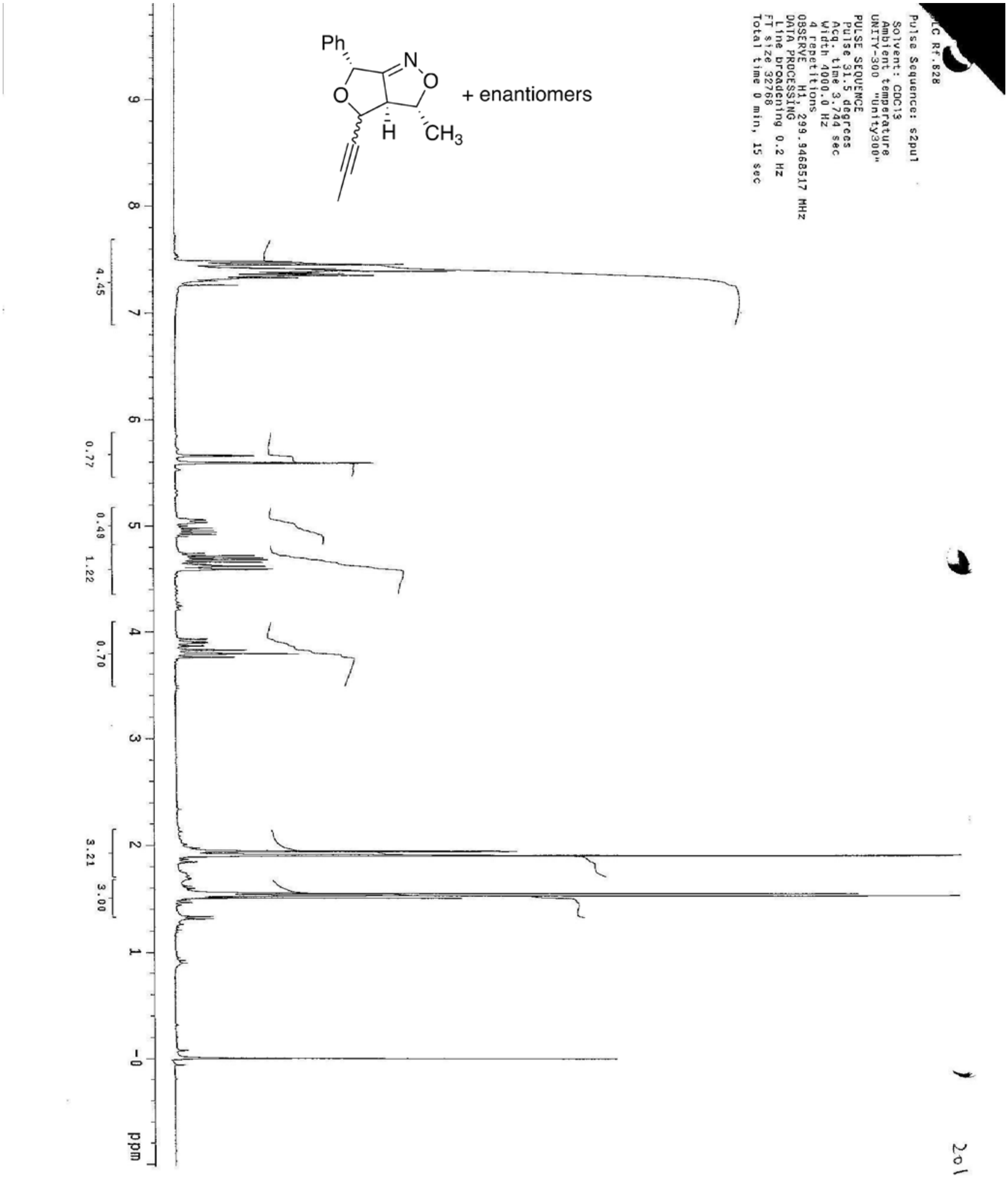

Expanded ^{1}H assignments for **10b**

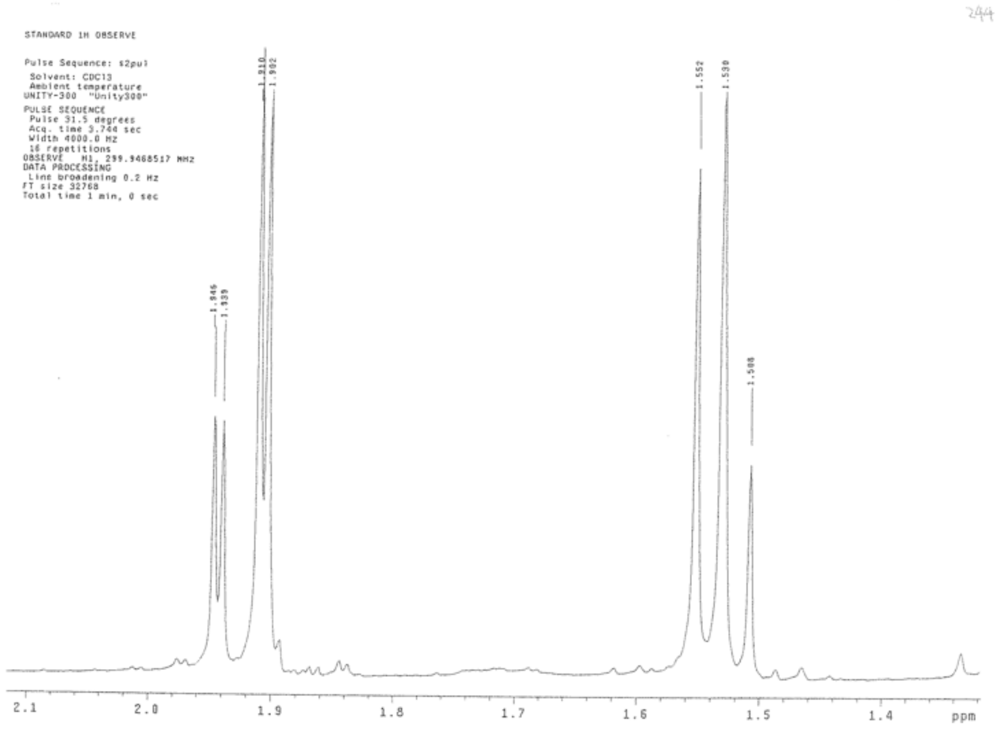

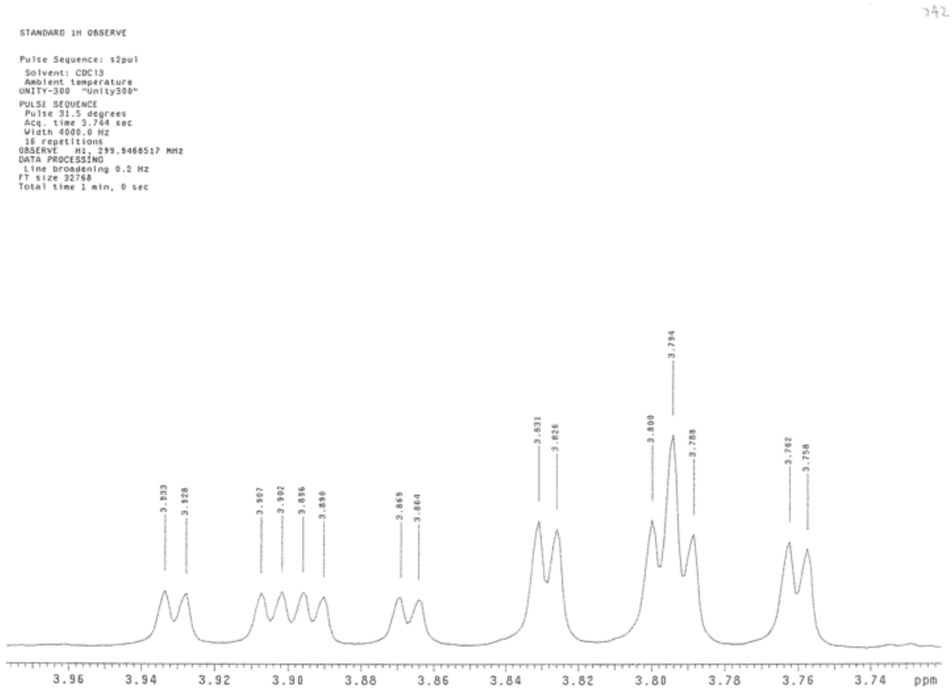

240

STANDARD 1H OBSERVE

Pulse Sequence: s2pul
Solvent: CDCl3
Ambient temperature
UNITY-300 "Unity300"
PULSE SEQUENCE
Pulse 31.5 degrees
Acq. time 3.744 sec
Width 4000.0 Hz
16 repetitions
OBSERVE H1, 299.9468517 MHz
DATA PROCESSING
Line broadening 0.2 Hz
FT size 32768
Total time 1 min, 0 sec

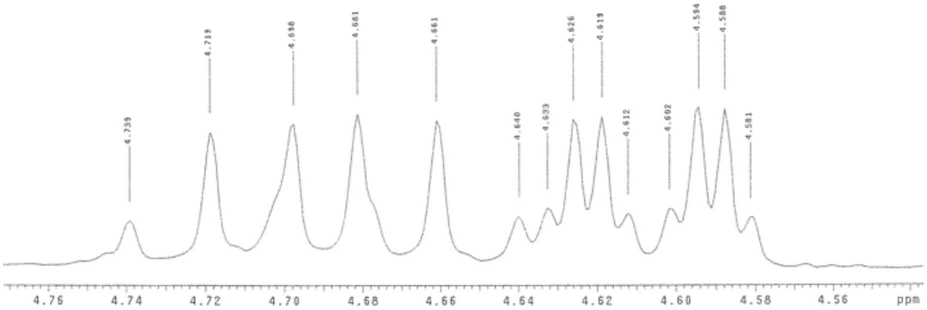

[illegible] TLC I

238

STANDARD 1H OBSERVE

Pulse Sequence: s2pul
Solvent: CDCl3
Ambient temperature
UNITY-300 "Unity300"
PULSE SEQUENCE
Pulse 31.5 degrees
Acq. time 3.744 sec
Width 4000.0 Hz
16 repetitions
OBSERVE H1, 299.9468517 MHz
DATA PROCESSING
Line broadening 0.2 Hz
FT size 32768
Total time 1 min, 0 sec

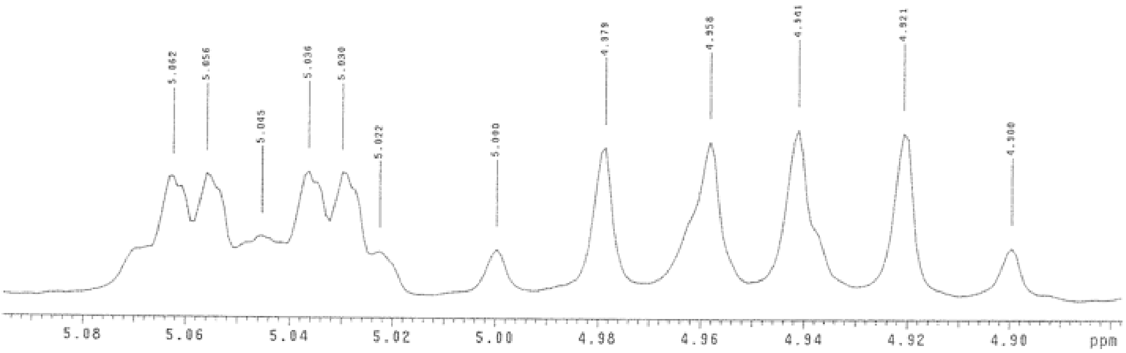

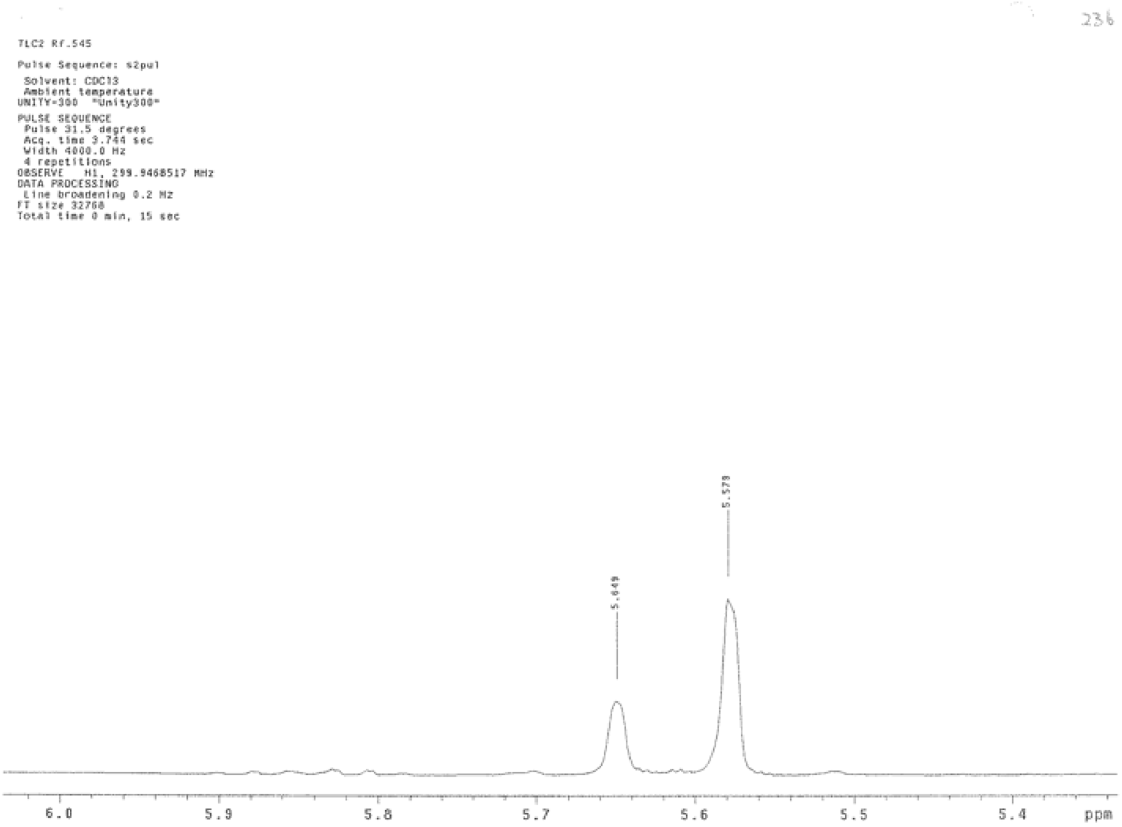
236
TLC2 Rf.545
Pulse Sequence: s2pul
Solvent: CDCl3
Ambient temperature
UNITY-300 "Unity300"
PULSE SEQUENCE
Pulse 31.5 degrees
Acq. time 3.744 sec
Width 4000.0 Hz
4 repetitions
OBSERVE H1, 299.9468517 MHz
DATA PROCESSING
Line broadening 0.2 Hz
FT size 32768
Total time 0 min, 15 sec
5.649
5.578
6.0
5.9
5.8
5.7
5.6
5.5
5.4
ppm

$^{13}C\{^{1}H\}$ NMR for (±)-(*3R,3aR,4S,6R*)--(3,3a-dihydro-3-methyl-6-phenyl-4-[prop-1-ynyl]-*4H,6H*-furo[3,4-c]isoxazole (**10b)**

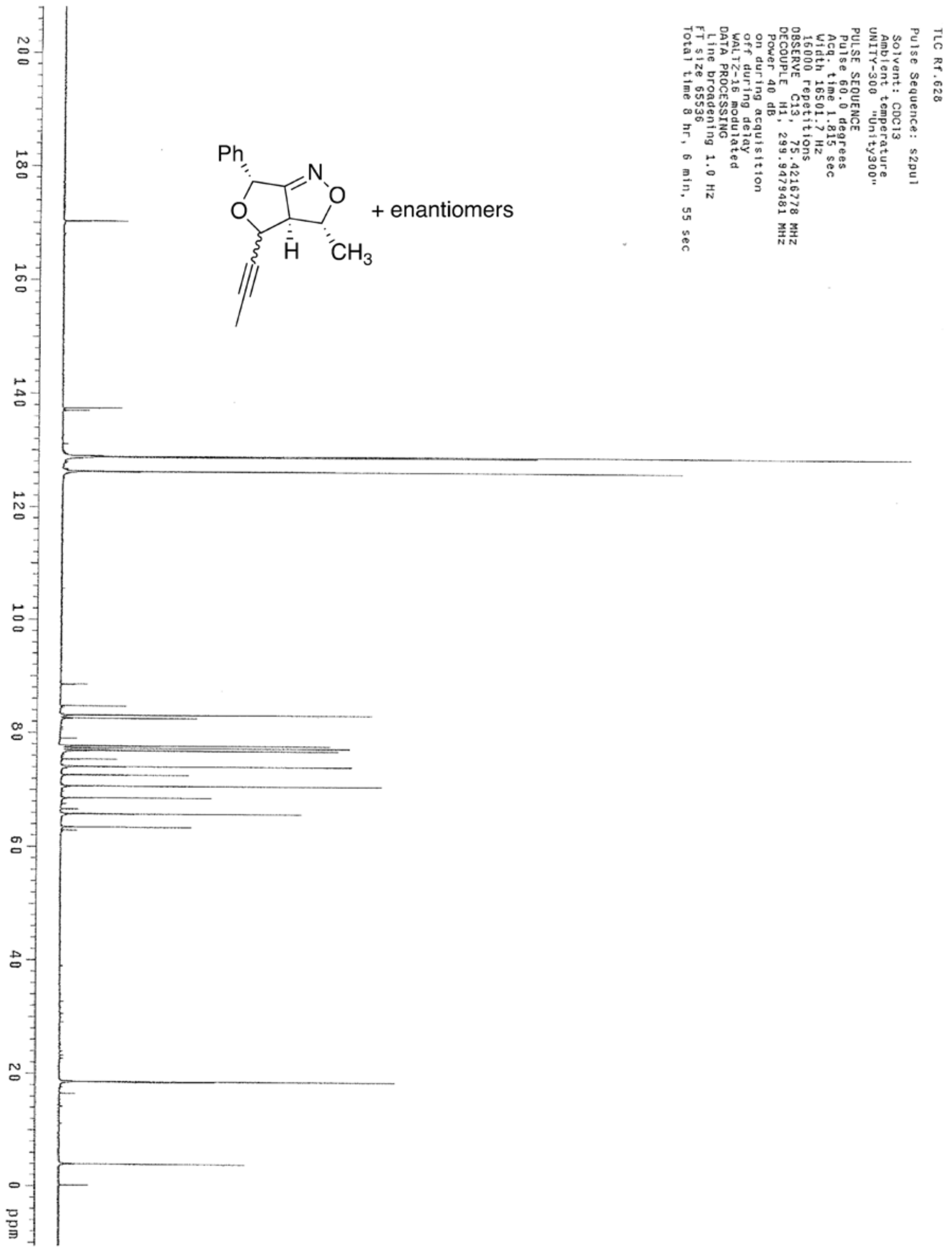

HMQC for (±)-(*3R,3aR,4S,6R*)-(3,3a)-dihydro-3-methyl-6-phenyl-4-[prop-1-ynyl]-*4H,6H*-furo[3,4-c]isoxazole (**10b)**

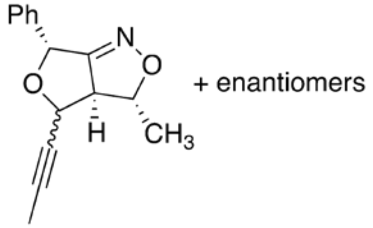

Isoxazole major & minor isomers HMQC

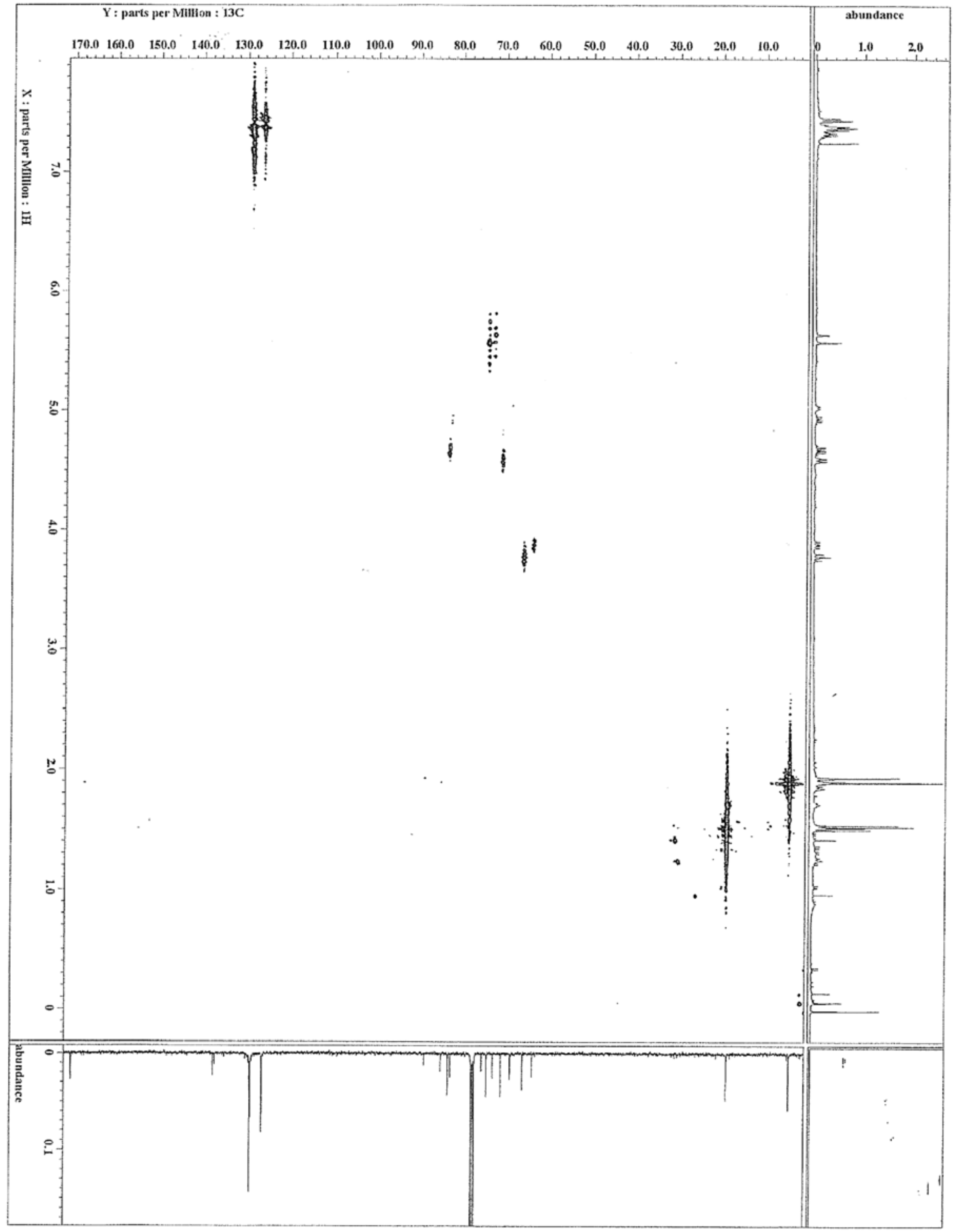

COSY for (±)-(*3R,3aR,4S,6R*)-(3,3a)-dihydro-3-methyl-6-phenyl-4-[prop-1-ynyl]-*4H,6H*-furo[3,4-c]isoxazole (**10b)**

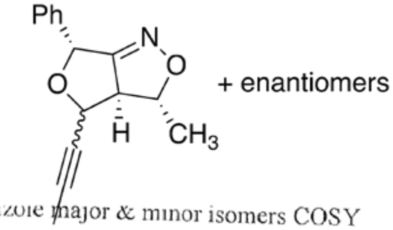

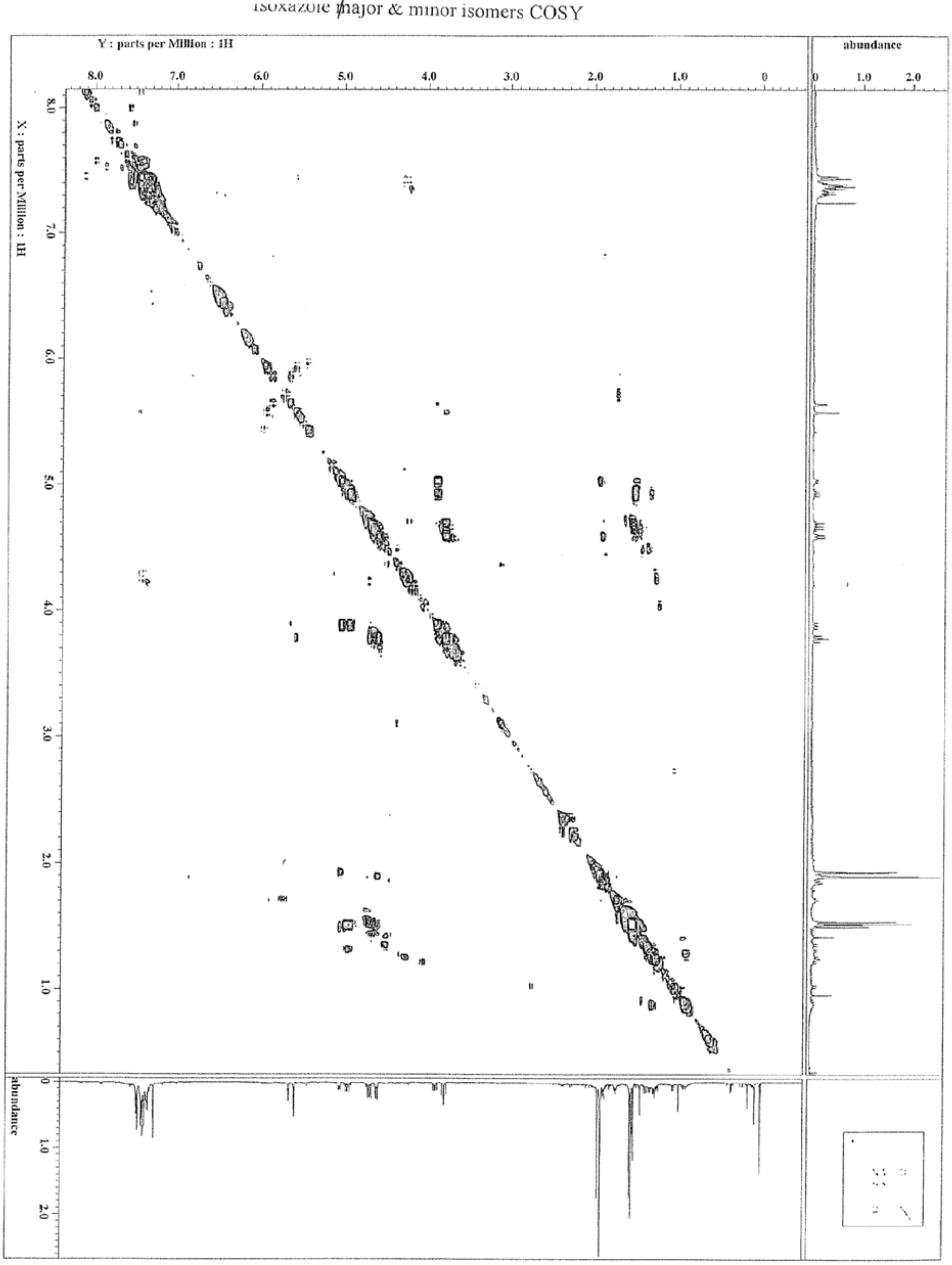

DQF COSY for (±)-(*3R,3aR,4S,6R*)--(3,3a)-dihydro-3-methyl-6-phenyl-4-[prop-1-ynyl]-*4H,6H*-
furo[3,4-c]isoxazole (**10b)**

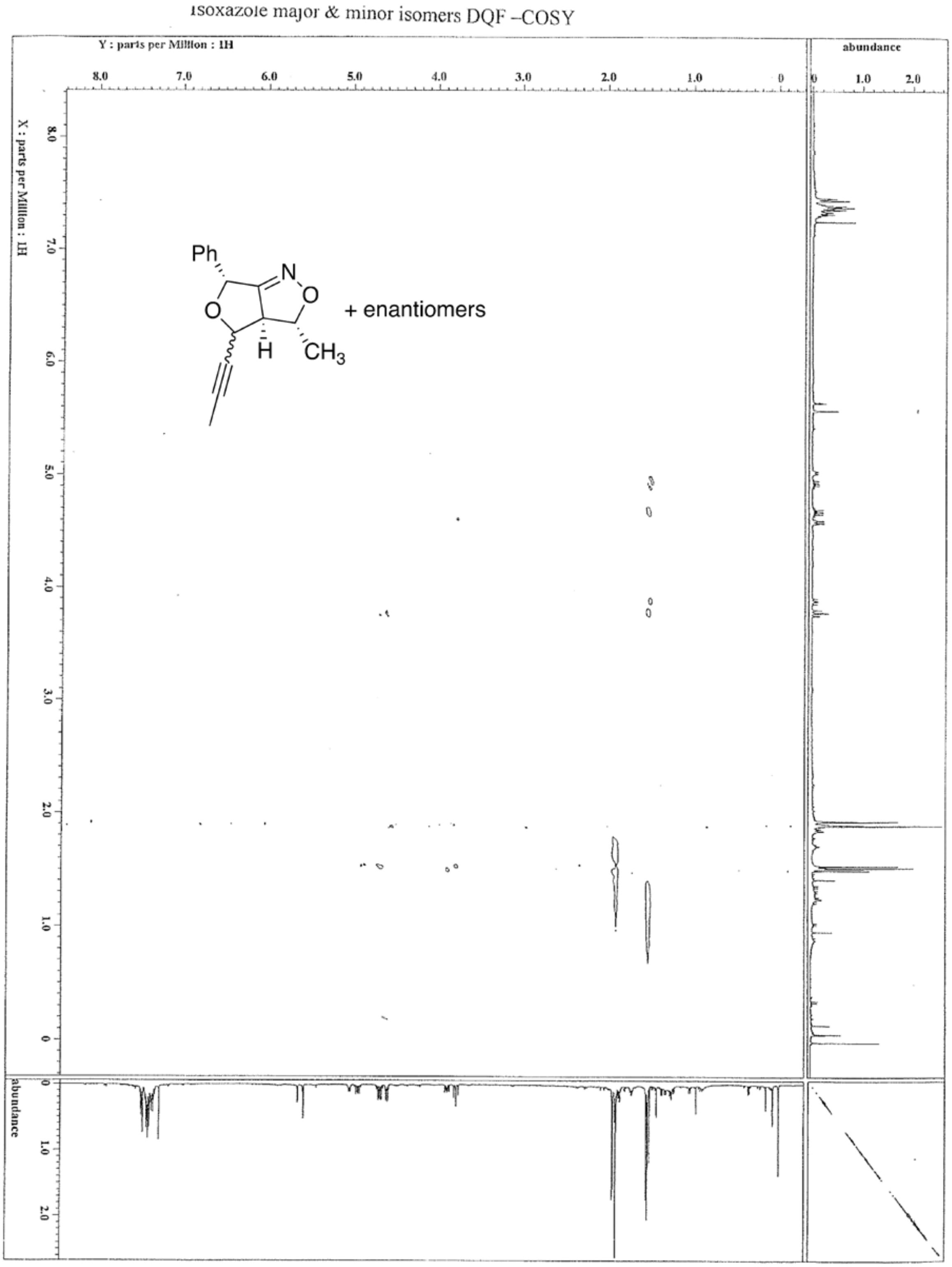

NOE for (±)-(*3R,3aR,4S,6R*)- (3,3a)-dihydro-3-methyl-6-phenyl-4-[prop-1-ynyl]-*4H,6H*-furo[3,4-c]isoxazole (**10b)**

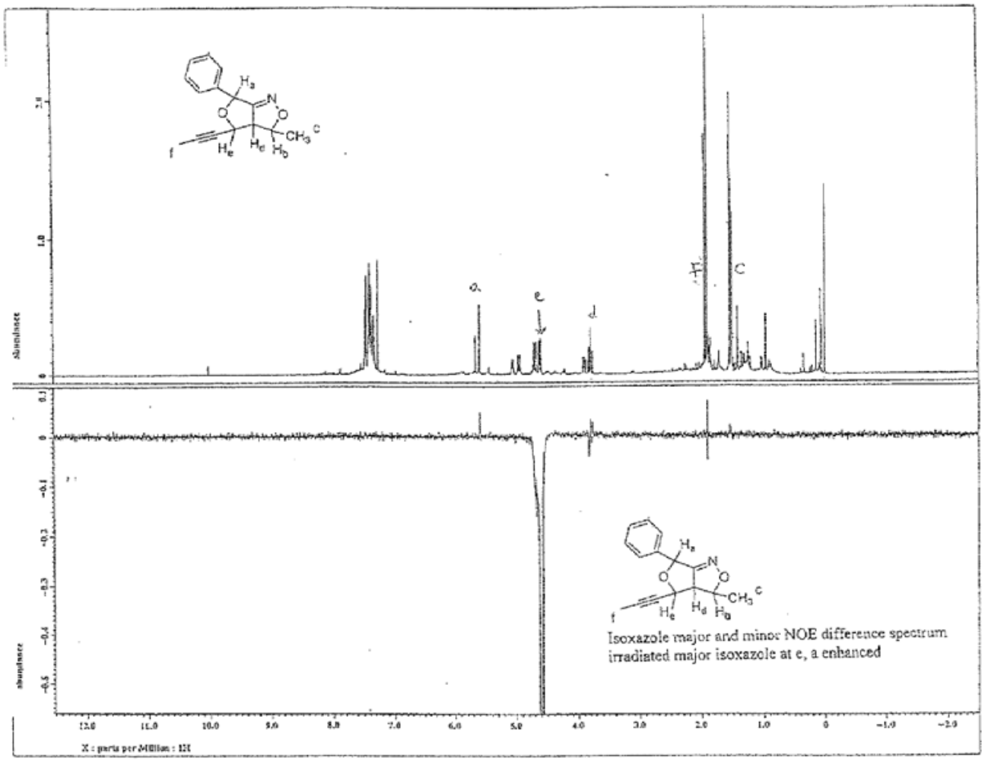

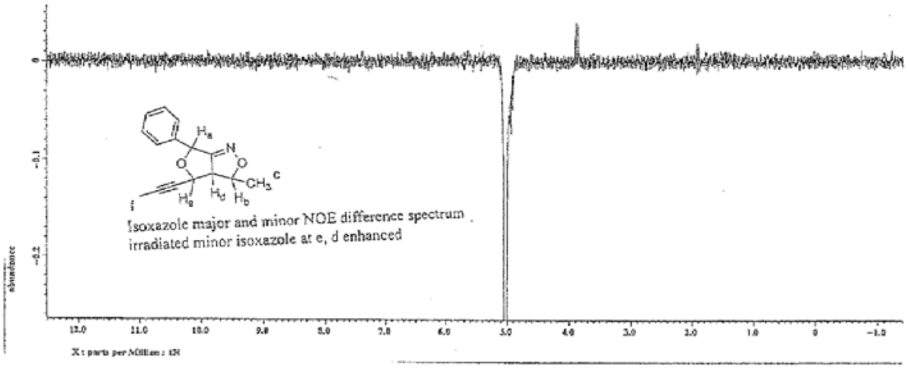

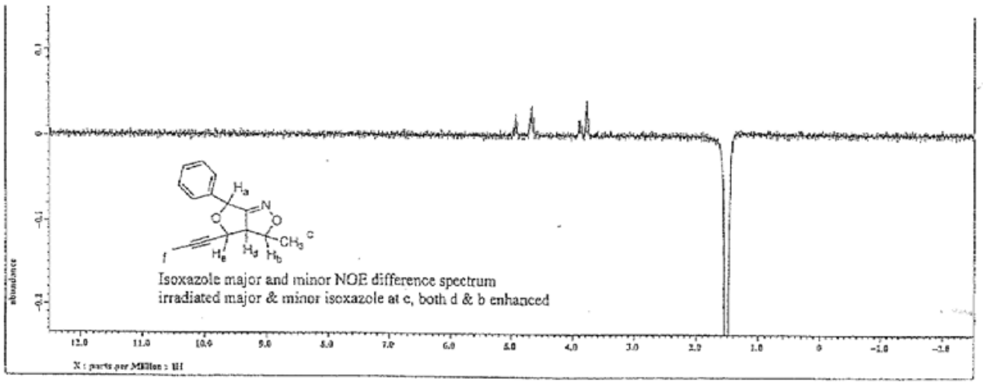

^{1}H NMR for (±)-(*E*)-hex-4-en-1-yn-3-ol **(14a)**

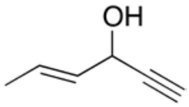

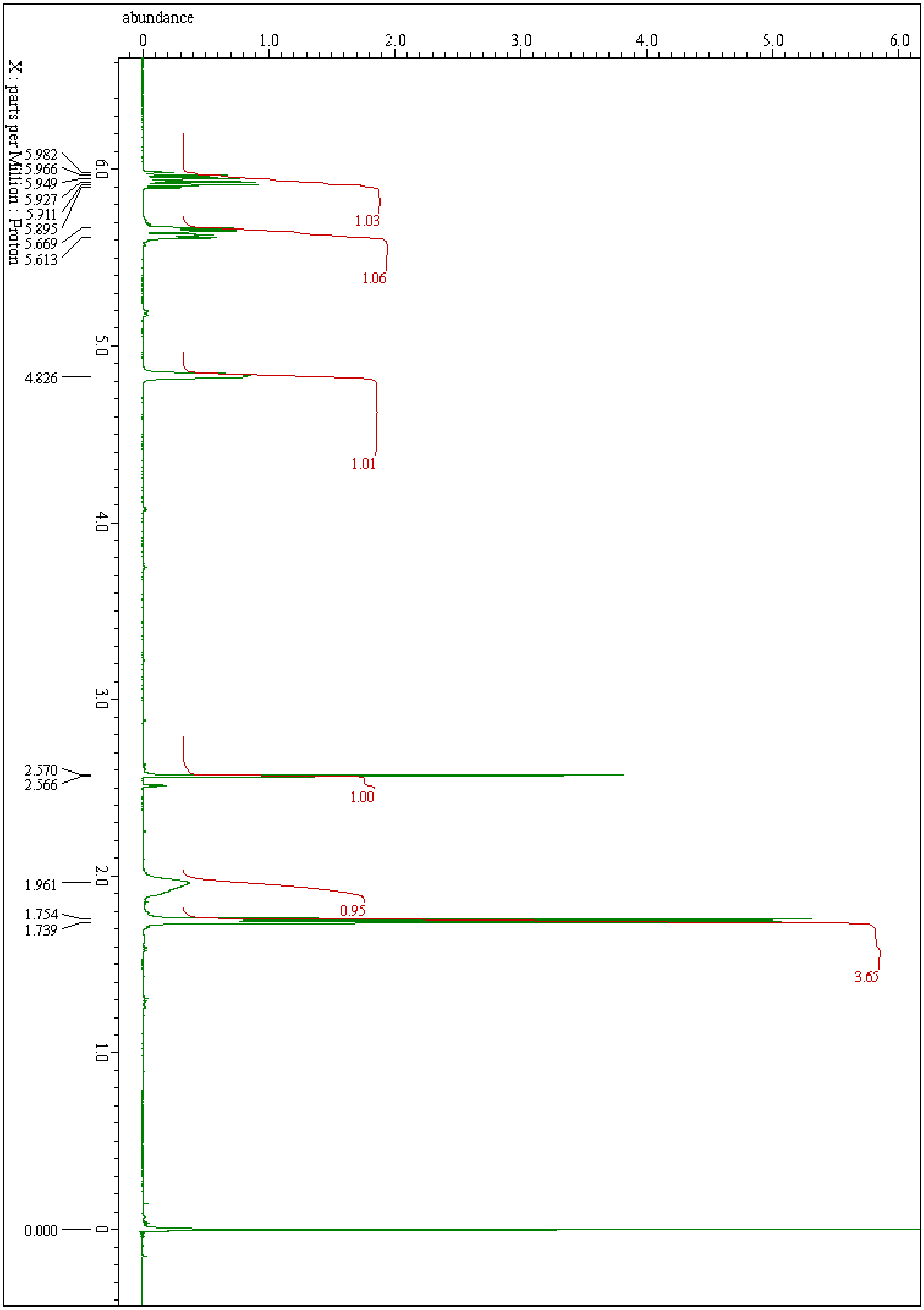

^{1}H NMR for (±)-(*E*-hept-5-en-2-yn-4-ol **(14b)**

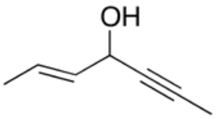

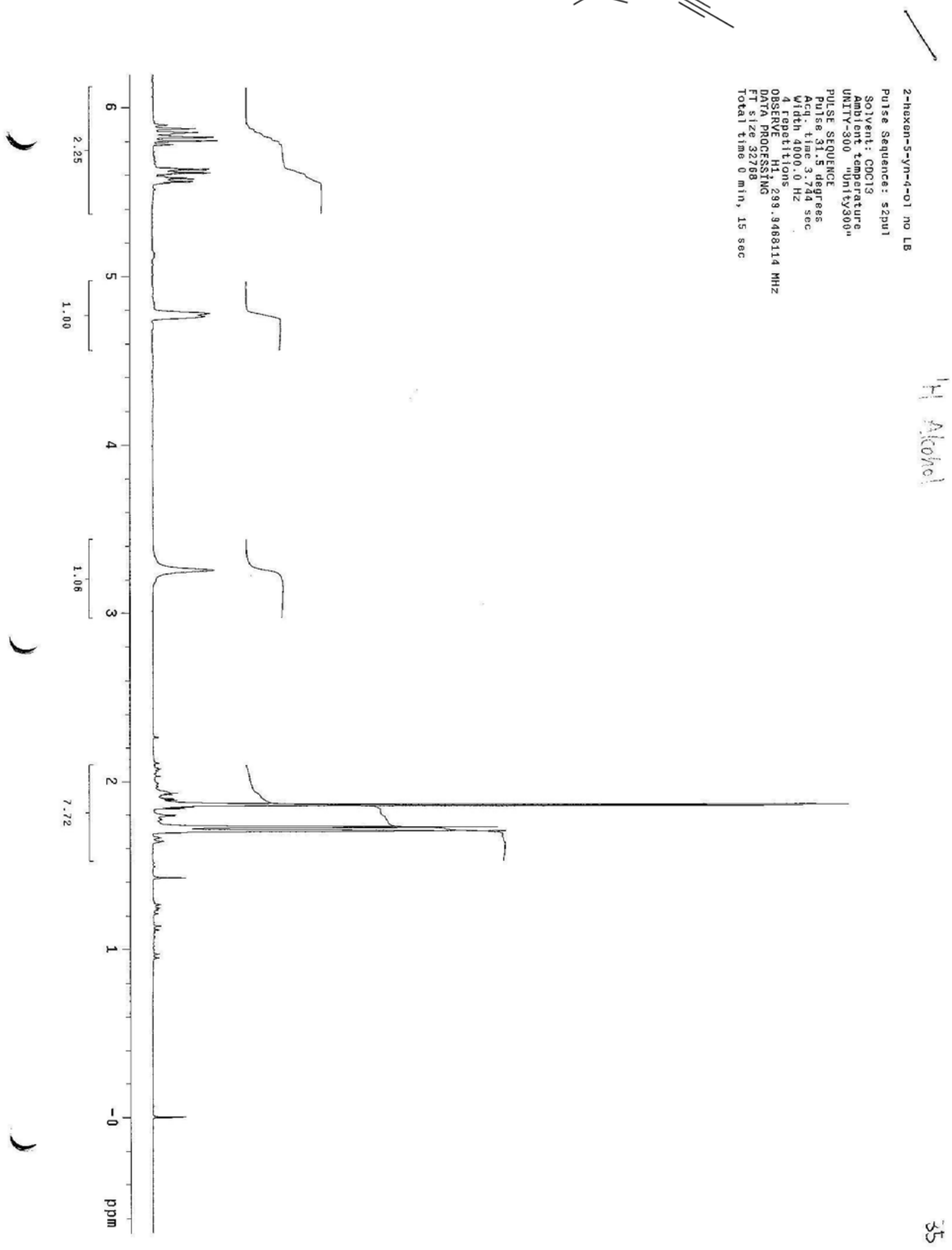

General Computational Methods

Spartan Experimental for Intermediates
1. Build molecule
    a. pay attention to geometry of stereocenters
    b. minimize energy
2. Set up calculations
    a. Click options
        1) type in PRINTCOORDS
    b. check frozen atoms if there are locked atoms present
    c. setup calculations as Equilibrium geometry with the following sequences
        Molecular Mechanics
            MMFF
        Semi-Empirical
            AM1
            PM3
            PM6
        Hartree-Fock/Density Functional (no locked atoms)
            6-31 G*/ωB97X-D

3. When beginning a new calculation, copy the most recent calculation and paste it into new file and save calculation as new file. With Hartree-Fock/DFT) started with PM6 file.
4. Results will be in output. Coordinates will be in verbose output.

Spartan Experimental for Transition States
1. Build molecule
    a. start with intermediate directly before the transition state
    b. set up mechanism using the arrow pushing icon
    c. press button in bottom right hand corner
        1) do not minimize the energy
2. If it can't calculate or the bond distances are not reasonable (less 3-2 Angstroms) then approximate better transition state
3. Set up calculations
    a. Click options
        1) type in PRINTCOORDS
    b. select IR (not for 6-31 G*/ωB97X-D)
    c. setup calculations as Transition State Geometry using same process as above except no MMFF
4. Results and coordinates in output box.
    a. List predicted IR peaks to confirm that there is only one negative Eigen value for valid transition state or check last output for energy.

PM6 Computational Methods

PM6 Geometry Optimization

Method 1.

```
SPARTAN'24 MECHANICS PROGRAM:   (Win/64b)                       Release
1.2.0
 Frequency Calculation
  Adjusted 9 (out of 144) low frequency modes
```

```
  Reason for exit: Successful completion
  Mechanics CPU Time :        0.547
  Mechanics Wall Time:       0.0870
SPARTAN'24 Semi-Empirical Program:  (Win/64b)            Release  1.2.0
  M0001
  Run type: Geometry optimization
           (Analytical Gradient)
  Model: RHF/PM6
  Number of shells:  72
    48 S shells
    23 P shells
     1 5D shells
  Number of basis functions:  122
  Number of electrons:  124
  Use of molecular symmetry disabled
  Molecular charge:      0
  Spin multiplicity:     1
  Point Group = C1  Order =  1 Nsymop =  1
  This system has  138 degrees of freedom
```

Method 2

```
SPARTAN '18 Semi-Empirical Program:  (Win/64b)           Release  1.0.0
  Run type: Geometry optimization
           (Analytical Gradient)
  Model: RHF/PM6
  Molecule (C1 ) and archive ( C1) have different symmetry
  Number of shells:  68
    45 S shells
    22 P shells
     1 5D shells
  Number of basis functions:  116
  Number of electrons:  118
  Use of molecular symmetry disabled
  Molecular charge:      0
  Spin multiplicity:     1
  Point Group = C1  Order =  1 Nsymop =  1
  This system has  129 degrees of freedom
```

Method 3

```
SPARTAN'24 Semi-Empirical Program:  (Win/64b)            Release  1.2.0
  M0001
  Run type: Geometry optimization
           (Analytical Gradient)
  Model: RHF/PM6
  Molecule (C1 ) and archive ( C1) have different symmetry
  Number of shells:  47
    30 S shells
    17 P shells
  Number of basis functions:   81
  Number of electrons:   86
  Use of molecular symmetry disabled
  Molecular charge:      0
  Spin multiplicity:     1
  Point Group = C1  Order =  1 Nsymop =  1
  This system has   84 degrees of freedom
```

<u>PM6 Transition State Optimization</u>

Method 1

```
SPARTAN '18 Semi-Empirical Program:  (Win/64b)          Release  1.4.0
  M0001
  Run type: Transition state optimization
           (Analytical Gradient)
           (Analytical Frequency in TSOPT)
           (Analytical Gradient in FREQ)
           (Numerical Frequency)
  Model: RHF/PM6
  Number of shells:  68
    45 S shells
    22 P shells
     1 5D shells
  Number of basis functions:  116
  Number of electrons:  118
  Use of molecular symmetry disabled
  Molecular charge:      0
  Spin multiplicity:     1
 Point Group = C1  Order =  1 Nsymop =  1
  This system has  129 degrees of freedom
  Initial Hessian option
  Hessian will be calculated numerically
  Estimating Force Constant matrix by central-differences
```

Method 2

```
SPARTAN '24 Semi-Empirical Program:  (Win/64b)          Release  1.0.0
  Run type: Transition state optimization
           (Analytical Gradient)
           (Analytical Frequency in TSOPT)
           (Analytical Gradient in FREQ)
           (Numerical Frequency)
  Model: RHF/PM6
  Number of shells:  72
    48 S shells
    23 P shells
     1 5D shells
  Number of basis functions:  122
  Number of electrons:  124
  Use of molecular symmetry disabled
  Molecular charge:      0
  Spin multiplicity:     1
  Point Group = C1  Order =  1 Nsymop =  1
  This system has  138 degrees of freedom
```

Cartesian Coordinates, calculational information for *cis*-**7a** (lowest energy conformer for R,R)
PM6 for Cis-7a
7a RR PM6 7a.RR.PM6 (DuffyGroup -> 2024)

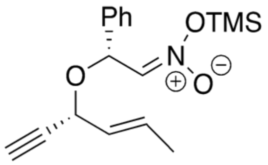

lowest conformer cis-**7a** (*R,R*)

```
Geometry Optimization Method 1
  Initial Hessian option
   Hessian from M1         calculation used.
                           Max.       Max.         Neg.
 Cycle        Energy       Grad.      Dist.        Eigen
     1        17.8097      0.09799    0.03171
   105       -49.7765      0.00021    0.00013
  Heat of Formation:         -49.777 kJ/mol
  Memory Used:             7.604 Mb
  Reason for exit: Successful completion
  Semi-Empirical Program CPU Time :          4.36
  Semi-Empirical Program Wall Time:          4.68
SPARTAN'24 Properties Program:  (Win/64b)                      Release  1.2.0
  Use of molecular symmetry disabled

                     Cartesian Coordinates (Angstroms)
       Atom            X              Y              Z
    ---------    ------------- ------------- -------------
  1 C  C8         0.4015412    -0.2456223     0.5276425
  2 N  N1         0.7973400    -1.2438720    -0.2270614
  3 O  O1         0.5437653    -1.5256910    -1.3854665
  4 O  O2         1.6550296    -2.1114142     0.4383600
  5 C  C1        -0.5128759     0.8250937    -0.0050387
  6 H  H5        -0.2605070     1.0957681    -1.0599001
  7 O  O3        -0.2149566     1.9496192     0.8614770
  8 C  C2        -0.6181654     3.2443531     0.3142141
  9 H  H4        -1.6719571     3.1566526    -0.0558617
 10 C  C3         0.3508611     3.6442753    -0.7693089
 11 C  C4        -0.0170461     4.3045062    -1.8696819
 12 C  C5        -0.5463808     4.1413736     1.4540898
 13 H  H3        -0.4436745     5.5256864     3.2090279
 14 C  C6        -0.4900282     4.8851848     2.3995287
 15 Si Si1        2.3689028    -3.6502059     0.4905077
 16 C  C7         3.5321581    -3.8506858    -0.9352409
 17 H  H12        4.3164675    -3.0784602    -0.9321307
 18 H  H14        2.9871312    -3.7696909    -1.8909195
 19 H  H15        4.0207456    -4.8371235    -0.8995430
 20 C  C9         1.0457814    -4.9453535     0.4562107
 21 H  H11        1.4955138    -5.9508348     0.4955332
 22 H  H16        0.4533158    -4.8723030    -0.4710815
 23 H  H17        0.3533561    -4.8467538     1.3059711
 24 C  C10        3.2600910    -3.5979180     2.1175928
 25 H  H13        3.9900501    -2.7742423     2.1513987
 26 H  H18        3.8010611    -4.5464586     2.2696711
 27 H  H19        2.5604123    -3.4597360     2.9561205
 28 C  C11       -1.9669728     0.4155653     0.1267244
 29 C  C12       -4.6449901    -0.3608657     0.3367691
 30 C  C13       -2.7228327     0.8047293     1.2388570
 31 C  C14       -2.5466827    -0.3747685    -0.8758352
 32 C  C15       -3.8852652    -0.7598511    -0.7684825
 33 C  C16       -4.0621676     0.4187619     1.3406525
 34 H  H2        -2.2534332     1.4070079     2.0202626
 35 H  H9        -1.9501754    -0.7001122    -1.7299295
 36 H  H10       -4.3362986    -1.3738717    -1.5459900
```

```
 37 H  H20          -4.6498222     0.7230062     2.2049333
 38 H  H21          -5.6880588    -0.6605994     0.4165055
 39 H  H1           -1.0555865     4.5937443    -2.0494672
 40 H  H8            0.7276906    -0.1330891     1.5679221
 41 H  H6            1.3814930     3.3487426    -0.5674506
 42 C  C17           0.9341581     4.7099220    -2.9422450
 43 H  H7            0.6505713     4.2698264    -3.9094717
 44 H  H22           1.9694980     4.4033656    -2.7388867
 45 H  H23           0.9409473     5.8023392    -3.0709787
```

Cartesian Coordinates, calculational information for *trans*-**7a** (lowest energy conformer for R,S)

Geometry Optimization Method 3

Cis7a PM6_Cis7a.spartan DuffyGroup - 11.2024 Good PM6 – not TS

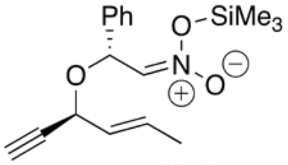

trans-**7a** (*R*,*S*)

```
SPARTAN'24 MECHANICS PROGRAM:   (Win/64b)                  Release  1.2.0
  Initial Hessian option
   Hessian from MMFF94     calculation used.
                           Max.       Max.         Neg.
 Cycle          Energy     Grad.      Dist.        Eigen
     1         26.1863    0.16887    0.06397
   100        -49.5980    0.00017    0.00028
M0001
  Heat of Formation:       -49.598 kJ/mol
  Estimating Force Constant matrix by central-differences
   Calculating Hessian
  Memory Used:           9.200 Mb
  Reason for exit: Successful completion
  Semi-Empirical Program CPU Time :        13.36
  Semi-Empirical Program Wall Time:        13.39
SPARTAN'24 Properties Program:  (Win/64b)                      Release  1.2.0
  Use of molecular symmetry disabled
                     Cartesian Coordinates (Angstroms)
       Atom              X             Y             Z
    ---------    ------------- ------------- -------------

  1 C  C8            0.5673845    -0.3563696    -0.1985861
  2 N  N1            1.1945965     0.7093176    -0.6383195
  3 O  O1            0.9283459     1.8949401    -0.5445324
  4 O  O2            2.3677416     0.4043530    -1.3204866
  5 C  C1           -0.6990938    -0.2454936     0.6079600
  6 H  H5           -0.6473140     0.5966964     1.3413461
  7 O  O3           -0.7227276    -1.5055852     1.3259982
  8 C  C2           -1.5701408    -1.4956799     2.5176531
  9 H  H4           -2.5615670    -1.0539123     2.2400662
 10 C  C3           -0.8737672    -0.7263836     3.6113021
 11 C  C4           -1.5249891     0.0182720     4.5076564
 12 C  C5           -1.7088489    -2.9010329     2.8563777
 13 C  C6           -1.8257653    -4.0666908     3.1353578
 14 Si Si1           3.3724003     0.7999915    -2.6318800
 15 C  C7            4.6493797    -0.5437640    -2.5506105
 16 H  H12           5.1717277    -0.5423243    -1.5809782
 17 H  H14           5.3967684    -0.3887035    -3.3460771
 18 H  H15           4.1996053    -1.5391263    -2.6867941
 19 C  C9            4.1058490     2.4804242    -2.3850518
 20 H  H11           4.7321251     2.7656741    -3.2447144
 21 H  H16           4.7274981     2.5201379    -1.4770201
 22 H  H17           3.3081319     3.2349635    -2.2730008
 23 C  C10           2.3802872     0.7320633    -4.1952201
 24 H  H13           1.8940162    -0.2458091    -4.3310424
 25 H  H18           3.0360211     0.9103408    -5.0634385
```

```
 26 H  H19           1.5932042     1.5031508    -4.1991046
 27 C  C11          -1.9131239    -0.1176205    -0.2916905
 28 C  C12          -4.1468361     0.1594267    -1.9508626
 29 C  C13          -2.6688911    -1.2429135    -0.6406681
 30 C  C14          -2.2657891     1.1467436    -0.7847811
 31 C  C15          -3.3829826     1.2819079    -1.6123222
 32 C  C16          -3.7873993    -1.1022154    -1.4668623
 33 H  H2           -2.3723210    -2.2244295    -0.2635008
 34 H  H9           -1.6598254     2.0198710    -0.5354158
 35 H  H10          -3.6574187     2.2629649    -1.9959945
 36 H  H20          -4.3761659    -1.9773075    -1.7356816
 37 H  H21          -5.0180426     0.2683161    -2.5938428
 38 H  H1           -2.6135412     0.1144690     4.4967062
 39 H  H8            0.9429322    -1.3709218    -0.3742599
 40 H  H6            0.2112287    -0.8407592     3.6057422
 41 C  C17          -0.8462948     0.7752892     5.5966392
 42 H  H7           -1.0695354     1.8501872     5.5300869
 43 H  H22           0.2473636     0.6710099     5.5776129
 44 H  H23          -1.1855325     0.4325051     6.5852616
 45 H  H6_1         -1.9286905    -5.0659738     3.3769727
  Point Group = C1  Order =  1 Nsymop =  1
  Temperature Corrections for   298.15 K
  Reason for exit: Successful completion
```

Cartesian Coordinates, calculational information for *cis*-**7b** (lowest energy conformer for R,R)
PM6 for Cis-7b
Geometry Optimization Method 3

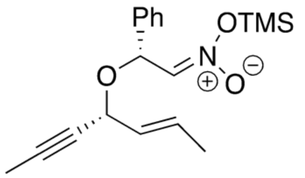

lowest conformer cis-**7b** (*R,R*)

```
Initial Hessian option
   Hessian from MMFF94     calculation used.
                        Max.      Max.        Neg.
 Cycle        Energy    Grad.     Dist.       Eigen
     1       -19.0976   0.17335   0.05882
    96       -93.3497   0.00024   0.00023
M0001
  Heat of Formation:      -93.350 kJ/mol
  Estimating Force Constant matrix by central-differences
   Calculating Hessian
  Memory Used:         10.224 Mb
  Reason for exit: Successful completion
  Semi-Empirical Program CPU Time :        15.36
  Semi-Empirical Program Wall Time:        15.37
SPARTAN'24 Properties Program:  (Win/64b)                    Release  1.2.0
  Use of molecular symmetry disabled
                     Cartesian Coordinates (Angstroms)
       Atom            X             Y             Z
    ---------    ------------- ------------- -------------

  1 C  C8           -0.1149240     0.1437435    -0.5491207
  2 N  N1            0.3368000     1.3002679    -0.9630155
  3 O  O1           -0.1887269     2.3851305    -1.1037213
  4 O  O2            1.6893116     1.2827499    -1.3122253
  5 C  C1           -1.5507325    -0.0360673    -0.1070886
  6 H  H5           -1.9713986     0.9092353     0.3132259
  7 O  O3           -1.5503736    -1.0734827     0.8938565
  8 C  C2           -1.5427724    -0.5954904     2.2804338
  9 H  H4           -2.4446112     0.0553411     2.4099998
 10 C  C3           -0.2538537     0.1297196     2.5704442
 11 C  C4           -0.1958158     1.2508981     3.2928330
 12 C  C5           -1.6632168    -1.8095719     3.0599672
```

```
 13 C  C6          -1.7636738    -2.8280228     3.6994883
 14 Si Si1          3.2394168     1.3944996    -1.7859989
 15 C  C7           3.5402346     0.0670556    -3.0487932
 16 H  H12          3.3438356    -0.9366170    -2.6453176
 17 H  H14          4.5938470     0.1101841    -3.3711785
 18 H  H15          2.9032042     0.2055686    -3.9355637
 19 C  C9           4.3003044     1.1227624    -0.2847269
 20 H  H11          5.3602687     1.1700584    -0.5852439
 21 H  H16          4.1164342     0.1413021     0.1747473
 22 H  H17          4.1234517     1.8957462     0.4770087
 23 C  C10          3.5499523     3.0713085    -2.5118128
 24 H  H13          2.9238518     3.2426445    -3.4012918
 25 H  H18          4.6070918     3.1622880    -2.8079878
 26 H  H19          3.3215359     3.8680853    -1.7870242
 27 C  C11         -2.3992694    -0.5434985    -1.2565095
 28 C  C12         -3.9716607    -1.4278806    -3.3919910
 29 C  C13         -2.8040364    -1.8817902    -1.3180473
 30 C  C14         -2.7777314     0.3551083    -2.2653191
 31 C  C15         -3.5630483    -0.0907149    -3.3305556
 32 C  C16         -3.5920169    -2.3212121    -2.3860679
 33 H  H2          -2.5043083    -2.5702574    -0.5237316
 34 H  H9          -2.4560341     1.3962933    -2.2226131
 35 H  H10         -3.8570364     0.6045673    -4.1148545
 36 H  H20         -3.9087758    -3.3614297    -2.4325181
 37 H  H21         -4.5839447    -1.7712956    -4.2231295
 38 H  H1          -1.0904048     1.7093960     3.7208421
 39 H  H8           0.5143901    -0.7502336    -0.4952386
 40 H  H6           0.6279685    -0.3457398     2.1403477
 41 C  C17          1.0729685     1.9749143     3.5862464
 42 H  H7           1.0096634     3.0274574     3.2734699
 43 H  H22          1.9438332     1.5407435     3.0759246
 44 H  H23          1.2911895     1.9663636     4.6638066
 45 C  C18         -1.8890200    -4.0285789     4.4699481
 46 H  H18c        -2.8108200    -4.0296267     5.0779784
 47 H  H18b        -1.0370660    -4.1602905     5.1603590
 48 H  H18a        -1.9242804    -4.9216324     3.8197594
  Point Group = C1  Order =  1 Nsymop =  1
  Temperature Corrections for   298.15 K
  Reason for exit: Successful completion
```

Cartesian Coordinates, calculational information for *trans*-**7b** (lowest energy conformer for R,S)
7b RS PM6   PM^.PC_7bRS.spartan (DuffyGroup_>2024)

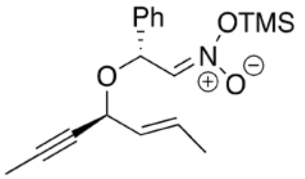

lowest conformer trans-**7b** (*R*,*S*)

```
Geometry Optimization Method 3
Initial Hessian option
   Hessian from MMFF94     calculation used.
                        Max.      Max.        Neg.
 Cycle        Energy    Grad.     Dist.       Eigen
     1       -21.2287   0.16662   0.06158
    64       -98.1624   0.00029   0.00013
M0001
  Heat of Formation:      -98.162 kJ/mol
  Estimating Force Constant matrix by central-differences
   Calculating Hessian
  Memory Used:         10.224 Mb
  Reason for exit: Successful completion
  Semi-Empirical Program CPU Time :        13.66
  Semi-Empirical Program Wall Time:        13.58
SPARTAN'24 Properties Program:  (Win/64b)                     Release  1.2.0
  Use of molecular symmetry disabled
```

Cartesian Coordinates (Angstroms)

	Atom		X	Y	Z
1	C	C8	0.5459072	0.1548147	-0.2222003
2	N	N1	1.2281718	1.2113233	-0.5950297
3	O	O1	1.0200288	2.4021859	-0.4449146
4	O	O2	2.4001897	0.8909576	-1.2771742
5	C	C1	-0.7250701	0.2808608	0.5774444
6	H	H5	-0.6398876	1.0761839	1.3588330
7	O	O3	-0.8254233	-1.0141530	1.2169694
8	C	C2	-1.6616868	-1.0409922	2.4157969
9	H	H2_1	-2.6549481	-0.5928321	2.1476052
10	Si	Si1	3.3904142	1.1977637	-2.6128524
11	C	C7	4.7050192	-0.0996398	-2.4321394
12	H	H12	5.2153763	-0.0174358	-1.4596648
13	H	H14	5.4564587	0.0258213	-3.2288286
14	H	H15	4.2868578	-1.1149085	-2.5057450
15	C	C9	4.0828537	2.9107081	-2.5136646
16	H	H11	4.6833466	3.1438112	-3.4066203
17	H	H16	4.7214903	3.0347569	-1.6249424
18	H	H17	3.2689252	3.6521416	-2.4403881
19	C	C10	2.3980750	0.9751849	-4.1623689
20	H	H13	1.9298652	-0.0194163	-4.2110414
21	H	H18	3.0526251	1.0881192	-5.0423132
22	H	H19	1.5976698	1.7283463	-4.2345224
23	C	C11	-1.9180458	0.5266992	-0.3268990
24	C	C12	-4.1119277	1.0242674	-1.9889211
25	C	C13	-2.7506436	-0.5270621	-0.7207713
26	C	C14	-2.1752916	1.8302712	-0.7754641
27	C	C15	-3.2723869	2.0756057	-1.6044073
28	C	C16	-3.8486678	-0.2763911	-1.5485768
29	H	H2	-2.5313030	-1.5397509	-0.3759512
30	H	H9	-1.5114060	2.6484056	-0.4885531
31	H	H10	-3.4726894	3.0873617	-1.9526645
32	H	H20	-4.4971128	-1.0961907	-1.8518888
33	H	H21	-4.9673871	1.2190572	-2.6324932
34	H	H8	0.8793716	-0.8648034	-0.4432107
35	C	C5	-1.8188818	-2.4987956	2.7662035
36	H	H5_1	-2.2902184	-2.6651302	3.7343451
37	C	C6	-1.4205400	-3.4968113	1.9732741
38	H	H6_1	-0.9313946	-3.2971540	1.0137129
39	C	C18	-1.5889069	-4.9379835	2.3126228
40	H	H18c	-0.6106445	-5.4344356	2.3967845
41	H	H18b	-2.1546975	-5.4645070	1.5310399
42	H	H18a	-2.1156241	-5.1005939	3.2620140
43	C	C3	-1.0294784	-0.2918559	3.4855696
44	C	C4	-0.4864342	0.3240825	4.3697833
45	C	C17	0.1495905	1.0634340	5.4183786
46	H	H17c	0.9976298	1.6594559	5.0346775
47	H	H17b	0.5498301	0.3949997	6.2013264
48	H	H17a	-0.5490024	1.7642232	5.9078305

```
Point Group = C1  Order =  1 Nsymop =  1
Temperature Corrections for   298.15 K
Reason for exit: Successful completion
```

Cartesian Coordinates, calculational information for *cis*-**9a** PM6

PM6 cis Ha (PM6_cisHa.spartan)

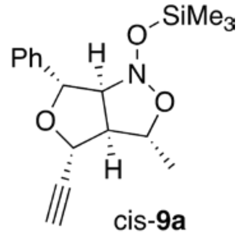

cis-**9a**

```
Geometry Optimization Method 2

   Hessian from M3         calculation used.
                                Max.      Max.        Neg.
 Cycle        Energy          Grad.     Dist.       Eigen
     1      -168.7895       0.10159   0.02710
    99      -210.2551       0.00045   0.00010
Heat of Formation:       -210.255 kJ/mol
  Memory Used:            7.604 Mb
  Reason for exit: Successful completion
  Semi-Empirical Program CPU Time :        10.33
  Semi-Empirical Program Wall Time:        10.45
SPARTAN '18 Properties Program:  (Win/64b)                        Release  1.0.0
  Use of molecular symmetry disabled
```

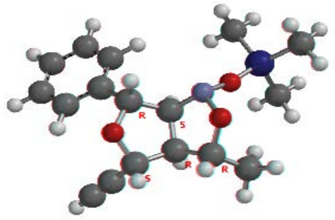

Cartesian Coordinates (Angstroms)

	Atom		X	Y	Z
1	C	C8	-0.6932589	0.0460221	0.2278309
2	N	N1	-1.0273912	0.1091121	-1.2518325
3	O	O1	-1.9031960	1.1537863	-1.4297256
4	O	O2	0.1558098	0.4428579	-1.9014927
5	C	C1	-1.4918907	-1.1188464	0.8676830
6	H	H5	-2.0908805	-1.7006104	0.1224453
7	O	O3	-2.5210189	-0.5127384	1.6903680
8	C	C2	-2.2309311	0.8827289	1.9576287
9	H	H4	-3.2399543	1.3601772	1.8825799
10	C	C3	-1.2360952	1.3520298	0.8699697
11	C	C4	-1.8961304	2.1119277	-0.2958536
12	C	C5	-1.6895719	1.0293648	3.2965341
13	H	H3	-0.8888320	1.2444331	5.3756918
14	C	C6	-1.2589469	1.1447615	4.4151406
15	Si	Si1	1.4356158	0.5453154	-2.8481149
16	C	C7	2.4730381	-0.9814108	-2.6203431
17	H	H12	1.9101146	-1.8818359	-2.9083889
18	H	H14	2.8117988	-1.1059055	-1.5837339
19	H	H15	3.3611010	-0.9057671	-3.2687536
20	C	C9	2.4025963	2.0498797	-2.3251633
21	H	H11	3.2565094	2.1645302	-3.0133357
22	H	H16	2.7938130	1.9516724	-1.3044372
23	H	H17	1.7951701	2.9626460	-2.3780734
24	C	C10	0.9149215	0.7047989	-4.6211227
25	H	H13	0.2624494	-0.1333610	-4.9119590
26	H	H18	1.7997558	0.7034002	-5.2759934
27	H	H19	0.3553700	1.6367982	-4.7906689
28	C	C11	-0.6185176	-2.0431677	1.6818430
29	C	C12	0.9840745	-3.8131717	3.1417800
30	C	C13	-0.8539937	-2.2378960	3.0474789
31	C	C14	0.4163535	-2.7432533	1.0426173
32	C	C15	1.2151063	-3.6244228	1.7743842
33	C	C16	-0.0508819	-3.1203748	3.7759570
34	H	H2	-1.6710690	-1.6977032	3.5323965
35	H	H9	0.5967903	-2.6040558	-0.0235768
36	H	H10	2.0178963	-4.1674389	1.2785080
37	H	H20	-0.2355977	-3.2688180	4.8385066
38	H	H21	1.6080021	-4.5011037	3.7090183
39	H	H1	-2.9806948	2.3020857	-0.1528044

			X	Y	Z
40	H	H8	0.4076379	-0.0725824	0.3743013
41	H	H6	-0.4132956	1.9499515	1.3196459
42	C	C17	-1.1450072	3.3530770	-0.7298750
43	H	H7	-0.0926983	3.1286778	-0.9647584
44	H	H22	-1.1673366	4.1393735	0.0315617
45	H	H23	-1.5767381	3.7650560	-1.6538638

Cartesian Coordinates, calculational information for *trans*-**9a** PM6

PM6 Trans Ha (PM6_Hatrans.spartan)

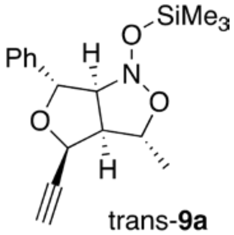

trans-**9a**

```
Geometry Optimization Method 2
  Initial Hessian option
   Hessian from M3         calculation used.
                        Max.      Max.        Neg.
 Cycle        Energy    Grad.     Dist.       Eigen
     1      -159.8956   0.09711   0.02734
   121      -201.2396   0.00021   0.00186
M0001
  Heat of Formation:     -201.240 kJ/mol
  Memory Used:          7.604 Mb
  Reason for exit: Successful completion
  Semi-Empirical Program CPU Time :        12.71
  Semi-Empirical Program Wall Time:        12.83
SPARTAN '18 Properties Program:  (Win/64b)                    Release  1.4.0
  Use of molecular symmetry disabled
                     Cartesian Coordinates (Angstroms)
       Atom           X             Y             Z
    ---------    ------------- ------------- -------------
```

	Atom		X	Y	Z
1	C	C8	-0.4523786	-0.0191479	0.4203925
2	N	N1	-1.0219001	0.1160592	-0.9788737
3	O	O1	-1.8876585	1.1810130	-0.9646178
4	O	O2	0.0480049	0.4587488	-1.8009829
5	C	C1	-1.2006345	-1.1657900	1.1503927
6	H	H5	-1.9784026	-1.6573357	0.5140034
7	O	O3	-1.9847377	-0.5550596	2.2049824
8	C	C2	-1.5032166	0.7951292	2.4783322
9	H	H4	-0.7595395	0.6946747	3.3103306
10	C	C3	-0.8059641	1.2853277	1.1862288
11	C	C4	-1.6800659	2.0825135	0.1981401
12	C	C5	-2.6503390	1.5619983	2.9114183
13	H	H3	-4.4308518	2.7155674	3.6272511
14	C	C6	-3.6086458	2.1818535	3.2963351
15	Si	Si1	1.1706118	0.5999860	-2.9214737
16	C	C7	2.4221629	-0.7555650	-2.6759156
17	H	H12	1.9551913	-1.7463474	-2.7720704
18	H	H14	2.9112560	-0.6988601	-1.6950912
19	H	H15	3.1962178	-0.6619769	-3.4554278
20	C	C9	1.9902164	2.2596373	-2.7093103
21	H	H11	2.4420309	2.3801008	-1.7168337
22	H	H16	1.2752031	3.0786944	-2.8698605
23	H	H17	2.7884596	2.3463294	-3.4655401
24	C	C10	0.4193632	0.4752078	-4.6125863
25	H	H13	-0.3402937	1.2567875	-4.7660257
26	H	H18	-0.0728900	-0.4991784	-4.7536552
27	H	H19	1.1996628	0.5887426	-5.3807776
28	C	C11	-0.2553505	-2.1939396	1.7276048
29	C	C12	1.4704802	-4.1410884	2.7584185
30	C	C13	-0.2615232	-2.4815035	3.0975618
31	C	C14	0.6086265	-2.8909807	0.8693481

```
 32 C  C15          1.4698641    -3.8606437     1.3873836
 33 C  C16          0.6030079    -3.4525465     3.6111986
 34 H  H2          -0.9544143    -1.9504556     3.7538951
 35 H  H9           0.6068842    -2.6810473    -0.2008350
 36 H  H10          2.1402300    -4.4015278     0.7211929
 37 H  H20          0.5957967    -3.6746599     4.6770233
 38 H  H21          2.1427263    -4.8976072     3.1587433
 39 H  H1          -2.7241334     2.2429067     0.5441338
 40 H  H8           0.6485788    -0.2005307     0.3743590
 41 H  H6           0.1071840     1.8609868     1.4508014
 42 C  C17         -1.0345223     3.3550477    -0.3088965
 43 H  H7          -0.0411317     3.1636359    -0.7429556
 44 H  H22         -0.9283472     4.1070279     0.4797216
 45 H  H23         -1.6347915     3.7978158    -1.1174633
```

Cartesian Coordinates, calculational information for *cis*-**9b** PM6

9b cis PM6 PM6_cisHb

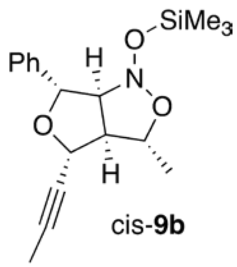

cis-**9b**

```
Geometry Optimization Method 1
  Initial Hessian option
   Hessian from M3         calculation used.
                        Max.       Max.         Neg.
 Cycle        Energy    Grad.      Dist.        Eigen
     1      -218.0643   0.08005   0.02654
    41      -259.0467   0.00053   0.00023
  Heat of Formation:      -259.047 kJ/mol
  Memory Used:          8.428 Mb
  Reason for exit: Successful completion
  Semi-Empirical Program CPU Time :          5.09
  Semi-Empirical Program Wall Time:          5.13
SPARTAN '18 Properties Program:  (Win/64b)                 Release  1.0.0
  Use of molecular symmetry disabled

                     Cartesian Coordinates (Angstroms)
      Atom              X             Y             Z
   ---------    ------------- ------------- -------------

  1 C  C8          -0.5403176     0.0957119    -0.1208582
  2 N  N1          -0.7036383     0.2235018    -1.6246587
  3 O  O1          -1.6807202     1.1705189    -1.8461923
  4 O  O2           0.5066005     0.7792882    -2.0713564
  5 C  C1          -1.2735983    -1.1857342     0.3512219
  6 H  H5          -1.6999230    -1.7816998    -0.4941393
  7 O  O3          -2.4600229    -0.7479186     1.0573459
  8 C  C2          -2.3633407     0.6508295     1.4400052
  9 H  H4          -3.4011453     1.0225977     1.2545067
 10 C  C3          -1.2966725     1.2920500     0.5195692
 11 C  C4          -1.8799787     2.0637300    -0.6794931
 12 C  C5          -2.0146874     0.7612607     2.8408545
 13 C  C6          -1.7377702     0.8338938     4.0132037
 14 Si Si1          1.6589567     0.5105866    -3.2426573
 15 C  C7           2.4478363    -1.1517761    -2.9810341
 16 H  H12          1.7046031    -1.9551022    -3.1016941
 17 H  H14          2.8951647    -1.2425504    -1.9819101
 18 H  H15          3.2427188    -1.3024635    -3.7294614
 19 C  C9           2.8760560     1.8863396    -2.9391093
 20 H  H11          3.3463284     1.8035100    -1.9491894
 21 H  H16          2.3885989     2.8696863    -3.0035632
 22 H  H17          3.6660375     1.8389844    -3.7059348
 23 C  C10          0.9273506     0.6111208    -4.9412546
```

			X	Y	Z
24	H	H13	0.0988049	-0.1098783	-5.0393675
25	H	H18	1.6806262	0.3839834	-5.7099314
26	H	H19	0.5183964	1.6138806	-5.1386083
27	C	C11	-0.4102636	-2.0611028	1.2291371
28	C	C12	1.1795922	-3.7396346	2.8072520
29	C	C13	-0.8101429	-2.3855105	2.5307616
30	C	C14	0.7849380	-2.5849052	0.7132532
31	C	C15	1.5774258	-3.4196893	1.5042544
32	C	C16	-0.0150191	-3.2235065	3.3177724
33	H	H2	-1.7478946	-1.9770871	2.9179872
34	H	H9	1.0951086	-2.3448539	-0.3040102
35	H	H10	2.5057013	-3.8236140	1.1049133
36	H	H20	-0.3302872	-3.4763184	4.3282666
37	H	H21	1.7980805	-4.3919269	3.4201257
38	H	H1	-2.9841068	2.1677616	-0.6562254
39	H	H8	0.5420617	0.0876067	0.1511568
40	H	H6	-0.6058984	1.9334044	1.1085105
41	C	C17	-1.1764138	3.3751898	-0.9665041
42	H	H7	-0.1054881	3.2108755	-1.1696238
43	H	H22	-1.2678711	4.0886518	-0.1419217
44	H	H23	-1.5839269	3.8469206	-1.8713890
45	C	C18	-1.4087669	0.9274085	5.4036388
46	H	H3	-0.7556401	0.0935680	5.7208843
47	H	H24	-2.3118929	0.8963395	6.0392423
48	H	H25	-0.8755574	1.8660717	5.6362241

Cartesian Coordinates, calculational information for *trans*-**9b** PM6

Trans 9b.PM6   PM6_Trans.Hb

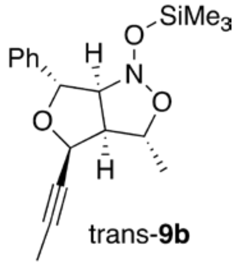

trans-**9b**

```
Geometry Optimization Method 1
  Initial Hessian option
   Hessian from MMFF94     calculation used.
                          Max.      Max.        Neg.
 Cycle        Energy      Grad.     Dist.       Eigen
     1      -233.5922    0.12564   0.09207
    11      -251.3321    0.00015   0.00009
  Heat of Formation:      -251.332 kJ/mol
  Memory Used:            8.428 Mb
     Reason for exit: Successful completion
  Semi-Empirical Program CPU Time :        0.844
  Semi-Empirical Program Wall Time:        0.843
SPARTAN'24 Properties Program:  (Win/64b)                    Release  1.2.0
  Use of molecular symmetry disabled
```

Cartesian Coordinates (Angstroms)

		Atom	X	Y	Z
1	C	C8	-0.1527939	-0.2182950	0.1538614
2	N	N1	-0.4704844	-0.1496460	-1.3272796
3	O	O1	-1.3529216	0.8852522	-1.5076311
4	O	O2	0.7190050	0.2016223	-1.9633014
5	C	C1	-0.9883375	-1.3631978	0.7867370
6	H	H5	-1.6275556	-1.9015627	0.0434586
7	O	O3	-1.9591477	-0.7441013	1.6631374
8	C	C2	-1.5681532	0.6315892	1.9666094
9	C	C3	-0.6698268	1.0985178	0.7941848
10	C	C4	-1.3833712	1.8290904	-0.3593291
11	Si	Si1	2.0207597	0.3492600	-2.8644872
12	C	C7	3.2620838	-0.9275888	-2.3226086
13	H	H12	2.8600045	-1.9438482	-2.4421082

```
 14 H  H14          3.5636783    -0.7974913    -1.2754585
 15 H  H15          4.1591750    -0.8343164    -2.9564958
 16 C  C9           2.7179146     2.0566951    -2.6000807
 17 H  H11          2.9652326     2.2490860    -1.5488721
 18 H  H16          2.0136544     2.8285394    -2.9407719
 19 H  H17          3.6419822     2.1459086    -3.1952616
 20 C  C10          1.6006519     0.1071596    -4.6550653
 21 H  H13          1.1926585    -0.8999928    -4.8299706
 22 H  H18          2.5018121     0.2335510    -5.2742693
 23 H  H19          0.8432500     0.8346148    -4.9842702
 24 C  C11         -0.1298965    -2.3400090     1.5574534
 25 C  C12          1.4441450    -4.1919029     2.9453578
 26 C  C13         -0.3573528    -2.5723637     2.9189912
 27 C  C14          0.8805783    -3.0449199     0.8859804
 28 C  C15          1.6654620    -3.9668344     1.5819842
 29 C  C16          0.4313480    -3.4957805     3.6113601
 30 H  H2          -1.1614973    -2.0352660     3.4269298
 31 H  H9           1.0523916    -2.8775054    -0.1779546
 32 H  H10          2.4498036    -4.5132909     1.0607812
 33 H  H20          0.2522231    -3.6746256     4.6701363
 34 H  H21          2.0574185    -4.9111196     3.4846392
 35 H  H1          -2.4765525     1.9565781    -0.2044378
 36 H  H8           0.9430716    -0.3655462     0.3084250
 37 H  H6           0.1662379     1.7147182     1.1886285
 38 C  C17         -0.7040146     3.1106605    -0.7952309
 39 H  H7           0.3534727     2.9435899    -1.0509664
 40 H  H22         -0.7548191     3.8863610    -0.0244158
 41 H  H23         -1.1744188     3.5083577    -1.7060395
 42 C  C18         -2.7872869     1.3750850     2.1668266
 43 C  C19         -3.8248926     1.9622974     2.3562182
 44 H  H24         -0.9749269     0.5784656     2.9148221
 45 C  C5          -5.0474196     2.6716215     2.5815404
 46 H  H5c         -4.8664755     3.7106203     2.9087701
 47 H  H5b         -5.6593701     2.1835812     3.3626407
 48 H  H5a         -5.6664992     2.7163822     1.6668316
```

Cartesian Coordinates, calculational information for *cis*-**10a** PM6

Cis 10a PM6 PRINTCOORDS   Cis10a.PM6

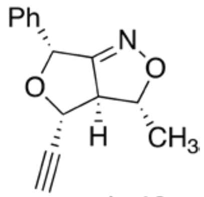

cis-**10a**

```
Geometry Optimization Method 3
  Initial Hessian option
   Hessian from M6         calculation used.
                             Max.       Max.         Neg.
 Cycle        Energy       Grad.      Dist.        Eigen
     1       209.5809    0.00026    0.00000
  Heat of Formation:       209.581 kJ/mol
  Memory Used:           3.437 Mb
  Reason for exit: Successful completion
  Semi-Empirical Program CPU Time :         0.219
  Semi-Empirical Program Wall Time:         0.428
SPARTAN'24 Properties Program:  (Win/64b)                      Release  1.2.0
  Use of molecular symmetry disabled

                     Cartesian Coordinates (Angstroms)
       Atom            X             Y             Z
    ---------    ------------- ------------- ------------

  1 C  C1           0.8492784     0.1523003     1.8287020
  2 H  H1           1.6128524    -0.1521593     2.5845441
  3 O  O1           1.6443304     0.8462700     0.8007141
```

			X	Y	Z
4	C	C2	1.5175683	0.2115334	-0.5138519
5	H	H2	2.5639611	0.2326667	-0.9013898
6	C	C3	1.0247819	-1.1541041	-0.1514565
7	C	C4	0.1718959	-1.0383115	1.1199359
8	H	H4c	-0.8857864	-0.7864312	0.8661071
9	C	C5	0.5768597	0.9805757	-1.4083778
10	C	C6	-1.1110766	2.4156690	-3.1125061
11	C	C7	0.0806164	2.2278117	-1.0116322
12	C	C8	0.2280212	0.4475251	-2.6577303
13	C	C9	-0.6155236	1.1677000	-3.5066786
14	C	C10	-0.7636938	2.9434736	-1.8652092
15	H	H7	0.3605351	2.6292077	-0.0339071
16	H	H8	0.6093808	-0.5264334	-2.9642011
17	H	H9	-0.8872307	0.7560504	-4.4774159
18	H	H10	-1.1485577	3.9142133	-1.5573680
19	H	H6	-1.7665485	2.9749313	-3.7775757
20	C	C11	-0.0949819	1.1034590	2.3754759
21	H	H12_1	-1.5290772	2.5973796	3.2262207
22	C	C12	-0.8664730	1.9081643	2.8312743
23	N	N1	1.3758333	-2.3735250	-0.4321751
24	O	O2	0.8976139	-3.2361151	0.5129727
25	C	C13	0.2841600	-2.4673905	1.6694019
26	H	H13_1	1.0238171	-2.5789617	2.4860912
27	C	C14	-1.0276175	-3.1539526	1.9618966
28	H	H14c	-0.8693583	-4.2109719	2.2231902
29	H	H14b	-1.6966088	-3.1512735	1.0889656
30	H	H14a	-1.5589721	-2.6793015	2.7959828

Cartesian Coordinates, calculational information for cis-***10b*** PM6

PM6 Cis Hb (PM6_CisHb.spartan)

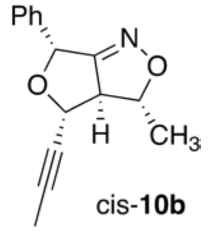

cis-**10b**

```
Geometry Optimization Method 1
  Initial Hessian option
   Hessian from M3          calculation used.
                          Max.      Max.         Neg.
 Cycle        Energy      Grad.     Dist.        Eigen
     1      -218.0643   0.08005   0.02654
    41      -259.0467   0.00053   0.00023
Heat of Formation:     -259.047 kJ/mol
  Memory Used:           8.428 Mb
  Reason for exit: Successful completion
  Semi-Empirical Program CPU Time :          5.09
  Semi-Empirical Program Wall Time:          5.13
SPARTAN '18 Properties Program:  (Win/64b)                    Release  1.0.0
  Use of molecular symmetry disabled
```

Cartesian Coordinates (Angstroms)

		Atom	X	Y	Z
1	C	C8	-0.5403176	0.0957119	-0.1208582
2	N	N1	-0.7036383	0.2235018	-1.6246587
3	O	O1	-1.6807202	1.1705189	-1.8461923
4	O	O2	0.5066005	0.7792882	-2.0713564
5	C	C1	-1.2735983	-1.1857342	0.3512219
6	H	H5	-1.6999230	-1.7816998	-0.4941393
7	O	O3	-2.4600229	-0.7479186	1.0573459
8	C	C2	-2.3633407	0.6508295	1.4400052
9	H	H4	-3.4011453	1.0225977	1.2545067
10	C	C3	-1.2966725	1.2920500	0.5195692
11	C	C4	-1.8799787	2.0637300	-0.6794931

```
 12 C  C5          -2.0146874     0.7612607     2.8408545
 13 C  C6          -1.7377702     0.8338938     4.0132037
 14 Si Si1          1.6589567     0.5105866    -3.2426573
 15 C  C7           2.4478363    -1.1517761    -2.9810341
 16 H  H12          1.7046031    -1.9551022    -3.1016941
 17 H  H14          2.8951647    -1.2425504    -1.9819101
 18 H  H15          3.2427188    -1.3024635    -3.7294614
 19 C  C9           2.8760560     1.8863396    -2.9391093
 20 H  H11          3.3463284     1.8035100    -1.9491894
 21 H  H16          2.3885989     2.8696863    -3.0035632
 22 H  H17          3.6660375     1.8389844    -3.7059348
 23 C  C10          0.9273506     0.6111208    -4.9412546
 24 H  H13          0.0988049    -0.1098783    -5.0393675
 25 H  H18          1.6806262     0.3839834    -5.7099314
 26 H  H19          0.5183964     1.6138806    -5.1386083
 27 C  C11         -0.4102636    -2.0611028     1.2291371
 28 C  C12          1.1795922    -3.7396346     2.8072520
 29 C  C13         -0.8101429    -2.3855105     2.5307616
 30 C  C14          0.7849380    -2.5849052     0.7132532
 31 C  C15          1.5774258    -3.4196893     1.5042544
 32 C  C16         -0.0150191    -3.2235065     3.3177724
 33 H  H2          -1.7478946    -1.9770871     2.9179872
 34 H  H9           1.0951086    -2.3448539    -0.3040102
 35 H  H10          2.5057013    -3.8236140     1.1049133
 36 H  H20         -0.3302872    -3.4763184     4.3282666
 37 H  H21          1.7980805    -4.3919269     3.4201257
 38 H  H1          -2.9841068     2.1677616    -0.6562254
 39 H  H8           0.5420617     0.0876067     0.1511568
 40 H  H6          -0.6058984     1.9334044     1.1085105
 41 C  C17         -1.1764138     3.3751898    -0.9665041
 42 H  H7          -0.1054881     3.2108755    -1.1696238
 43 H  H22         -1.2678711     4.0886518    -0.1419217
 44 H  H23         -1.5839269     3.8469206    -1.8713890
 45 C  C18         -1.4087669     0.9274085     5.4036388
 46 H  H3          -0.7556401     0.0935680     5.7208843
 47 H  H24         -2.3118929     0.8963395     6.0392423
 48 H  H25         -0.8755574     1.8660717     5.6362241
```

Cartesian Coordinates, calculational information for *cis*-**11a** PM6

Spartan 10 for 9a E (9a, cis)

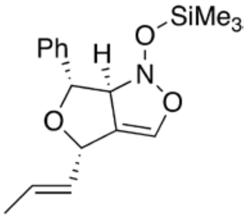

cis-**11a**

```
Geometry Optimization Method 2
  Initial Hessian option
   Hessian from MMFF94     calculation used.
                          Max.      Max.        Neg.
 Cycle        Energy      Grad.     Dist.       Eigen
     1      -189.7776   0.08407   0.02966
    88      -238.6153   0.00016   0.00015
  Heat of Formation:     -238.615 kJ/mol
  Memory Used:          7.604 Mb
  Reason for exit: Successful completion
  Semi-Empirical Program CPU Time :        16.97
  Semi-Empirical Program Wall Time:      1:27.47
SPARTAN '14 Properties Program:  (Win/64b)                  Release  1.1.4
  Use of molecular symmetry disabled

                     Cartesian Coordinates (Angstroms)
       Atom            X             Y             Z
    ---------    ------------- ------------- -------------
  1 C  C8           0.8566666     0.2076727    -0.1633328
```

```
  2 N  N1          1.4317304   -0.8551388    0.7588625
  3 O  O1          2.5679731   -0.2117837    1.4011925
  4 O  O2          0.4960954   -0.9752351    1.7706174
  5 C  C1          0.8064600   -0.1401766   -1.6704771
  6 H  H5          1.5076429   -0.9587668   -1.9508631
  7 O  O3          1.3369957    1.0412655   -2.3585461
  8 C  C2          1.7757941    2.0810597   -1.4095693
  9 C  C5          1.9018441    1.3163400   -0.1405634
 10 H  H3          3.7048275    1.4908373    1.1748800
 11 C  C6          2.8205394    1.0248107    0.7873352
 12 Si Si1         0.1040561   -2.3427896    2.7419431
 13 C  C7          1.5691786   -2.8771994    3.7380625
 14 H  H12         2.3976646   -3.1723945    3.0720170
 15 H  H14         1.3217693   -3.7339590    4.3829417
 16 H  H15         1.9355047   -2.0579677    4.3768487
 17 C  C9         -0.5007574   -3.7283526    1.6675046
 18 H  H11        -0.7168696   -4.6155385    2.2846598
 19 H  H16         0.2653932   -4.0077148    0.9262351
 20 H  H17        -1.4186148   -3.4596931    1.1251066
 21 C  C10        -1.2501843   -1.6214059    3.7872729
 22 H  H13        -1.6301482   -2.3857423    4.4838930
 23 H  H18        -2.0905759   -1.2646748    3.1724090
 24 H  H19        -0.8812516   -0.7676736    4.3774989
 25 C  C11        -0.5862617   -0.4164059   -2.1780459
 26 C  C12        -3.1512764   -0.9925984   -3.1368321
 27 C  C13        -1.1058633    0.2993553   -3.2637155
 28 C  C14        -1.3510423   -1.4246804   -1.5726951
 29 C  C15        -2.6310059   -1.7093895   -2.0535604
 30 C  C16        -2.3876982    0.0109760   -3.7404043
 31 H  H2         -0.4976855    1.0784364   -3.7302380
 32 H  H9         -0.9485645   -1.9830583   -0.7261003
 33 H  H10        -3.2246328   -2.4913844   -1.5838165
 34 H  H20        -2.7894189    0.5681923   -4.5845978
 35 H  H21        -4.1485430   -1.2172971   -3.5092891
 36 H  H8         -0.1445543    0.5528691    0.2078596
 37 C  C18         0.7488042    3.1752895   -1.3897989
 38 H  H24        -0.1096849    2.9840638   -2.0362937
 39 C  C19         0.8757946    4.2837488   -0.6568730
 40 H  H25         1.7400417    4.4494309   -0.0105392
 41 C  C20        -0.1329974    5.3807208   -0.6410088
 42 H  H4          0.3210975    6.3409049   -0.9239880
 43 H  H26        -0.5612939    5.5046339    0.3643845
 44 H  H27        -0.9714037    5.2037516   -1.3282151
 45 H  H28         2.7444548    2.4166616   -1.8421609
```

Cartesian Coordinates, calculational information for *trans*-**11a** PM6

PM6 for Trans-11a

PM6 Trans-Ea (PM6_TransEa.spartan)

Trans 9a

```
Geometry Optimization Method 2
  This system has  129 degrees of freedom
  Initial Hessian option
   Hessian from M3        calculation used.
                        Max.      Max.        Neg.
 Cycle        Energy    Grad.     Dist.       Eigen
     1      -189.3241   0.08644   0.03985
     102      -237.8685   0.00010   0.00006
Heat of Formation:     -237.869 kJ/mol
```

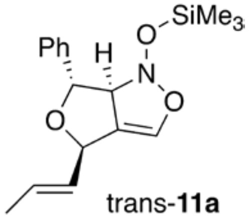

trans-**11a**

```
  Memory Used:          7.604 Mb
  Reason for exit: Successful completion
  Semi-Empirical Program CPU Time :        10.51
  Semi-Empirical Program Wall Time:        10.61
SPARTAN '18 Properties Program:  (Win/64b)                    Release  1.0.0
  Reason for exit: Successful completion
  Properties CPU Time :          .55
  Properties Wall Time:          .55
SPARTAN '18 Properties Program:  (Win/64b)                    Release  1.4.0
  Use of molecular symmetry disabled
                      Cartesian Coordinates (Angstroms)
       Atom            X             Y             Z
    ---------    ------------- ------------- -------------
  1 C  C8          0.1684473     0.4334420     0.0261291
  2 N  N1          0.7119874    -0.6715717     0.9173408
  3 O  O1          1.9096485    -0.1046143     1.5205526
  4 O  O2         -0.1889808    -0.7440168     1.9634803
  5 C  C1          0.0633808     0.1159261    -1.4855288
  6 H  H5          0.7946288    -0.6563172    -1.8133873
  7 O  O3          0.4716004     1.3560525    -2.1517708
  8 C  C2          1.1517374     2.2827568    -1.2209362
  9 H  H4          0.4550002     3.1464335    -1.1345461
 10 C  C3          2.4576386     2.6494214    -1.8607909
 11 C  C4          3.2051220     3.6779043    -1.4533058
 12 C  C5          1.2719702     1.4829294     0.0268768
 13 H  H3          3.1368841     1.5309048     1.2632115
 14 C  C6          2.2099416     1.1216563     0.9115797
 15 Si Si1        -0.6272114    -2.0845512     2.9512922
 16 C  C7          0.8351781    -2.6953611     3.9069417
 17 H  H12         1.2703725    -1.8925783     4.5229984
 18 H  H14         1.6227246    -3.0482557     3.2199094
 19 H  H15         0.5565492    -3.5278872     4.5706343
 20 C  C9         -1.3403511    -3.4390134     1.9041456
 21 H  H11        -1.6193716    -4.2966850     2.5375474
 22 H  H16        -0.6035659    -3.7881869     1.1631483
 23 H  H17        -2.2393031    -3.1078296     1.3638111
 24 C  C10        -1.9071956    -1.2853174     4.0329438
 25 H  H13        -2.2927620    -2.0192134     4.7585279
 26 H  H18        -2.7544554    -0.9043184     3.4423789
 27 H  H19        -1.4802613    -0.4375596     4.5916632
 28 C  C11        -1.3342432    -0.2187728    -1.9382907
 29 C  C12        -3.9108943    -0.9037144    -2.7863737
 30 C  C13        -1.9850633     0.5616653    -2.9015813
 31 C  C14        -1.9737185    -1.3449401    -1.3984918
 32 C  C15        -3.2600141    -1.6836431    -1.8237982
 33 C  C16        -3.2725532     0.2178605    -3.3236445
 34 H  H2         -1.4755032     1.4353144    -3.3159441
 35 H  H9         -1.4692743    -1.9509329    -0.6446137
 36 H  H10        -3.7568125    -2.5565610    -1.4048584
 37 H  H20        -3.7766524     0.8255323    -4.0728768
 38 H  H21        -4.9127068    -1.1708773    -3.1162233
 39 H  H1          2.9038727     4.3135772    -0.6180636
 40 H  H8         -0.8053078     0.8186336     0.4265079
 41 H  H6          2.7329566     2.0052124    -2.6987554
 42 C  C17         4.4998306     4.0557180    -2.0874133
 43 H  H7          4.4364894     5.0562939    -2.5403103
 44 H  H22         4.8077240     3.3623951    -2.8820852
 45 H  H23         5.3125222     4.0830892    -1.3480308
```

Cartesian Coordinates, calculational information for *cis*-**11b** PM6

11b cis PM6 PM6_cisEb

```
Geometry Optimization Method 1
  Initial Hessian option
   Hessian from M3        calculation used.
                          Max.      Max.        Neg.
 Cycle        Energy      Grad.     Dist.       Eigen
     1      -234.8386   0.08133   0.03067
   162      -285.0876   0.00015   0.00028
  Heat of Formation:      -285.088 kJ/mol
  Memory Used:            8.428 Mb
  Reason for exit: Successful completion
  Semi-Empirical Program CPU Time :        19.05
  Semi-Empirical Program Wall Time:        19.19
SPARTAN '18 Properties Program:  (Win/64b)                   Release  1.0.0
  Use of molecular symmetry disabled
```

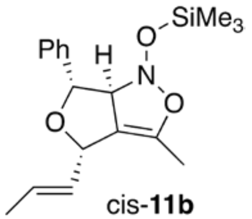

cis-**11b**

Cartesian Coordinates (Angstroms)

	Atom		X	Y	Z
1	C	C8	0.5700894	0.0952355	-0.2163257
2	N	N1	1.1572912	-0.9827056	0.6836211
3	O	O1	2.2683550	-0.3374085	1.3587589
4	O	O2	0.2101693	-1.1535255	1.6782690
5	C	C1	0.5402317	-0.2220814	-1.7319472
6	H	H5	1.2511481	-1.0289564	-2.0204921
7	O	O3	1.0658531	0.9763140	-2.3900759
8	C	C2	1.4799195	2.0022114	-1.4131437
9	C	C5	1.5913352	1.2189482	-0.1564570
10	C	C6	2.5109798	0.9291007	0.7771216
11	Si	Si1	-0.1815951	-2.5521393	2.5983001
12	C	C7	1.2754801	-3.1099776	3.5943416
13	H	H12	2.1110010	-3.3832307	2.9281086
14	H	H14	1.0238502	-3.9851789	4.2119830
15	H	H15	1.6319154	-2.3074406	4.2590352
16	C	C9	-0.7635821	-3.9072271	1.4731547
17	H	H11	-0.9754373	-4.8167935	2.0581949
18	H	H16	0.0111276	-4.1531796	0.7289716
19	H	H17	-1.6798819	-3.6285364	0.9329543
20	C	C10	-1.5537424	-1.8789117	3.6527943
21	H	H13	-1.1965614	-1.0493869	4.2831359
22	H	H18	-1.9418942	-2.6726817	4.3107661
23	H	H19	-2.3855208	-1.4988440	3.0401282
24	C	C11	-0.8452723	-0.5010641	-2.2583642
25	C	C12	-3.3970432	-1.0798342	-3.2509901
26	C	C13	-1.3730220	0.2498203	-3.3160234
27	C	C14	-1.5953568	-1.5459710	-1.6982488
28	C	C15	-2.8685325	-1.8321202	-2.1960565
29	C	C16	-2.6481235	-0.0398236	-3.8096185
30	H	H2	-0.7753480	1.0567740	-3.7478148
31	H	H9	-1.1866535	-2.1312147	-0.8732208
32	H	H10	-3.4502640	-2.6430454	-1.7618233
33	H	H20	-3.0559727	0.5448480	-4.6319699
34	H	H21	-4.3890485	-1.3052984	-3.6365100
35	H	H8	-0.4407473	0.4094987	0.1542769
36	C	C18	0.4352869	3.0804498	-1.3858122
37	H	H24	-0.5022973	2.8008998	-1.8690567
38	C	C19	0.6369033	4.2749374	-0.8249466
39	H	H25	1.5818761	4.5321655	-0.3436459

```
 40 C  C20          -0.3910836     5.3542080    -0.7930213
 41 H  H4           -0.0197473     6.2684261    -1.2773346
 42 H  H26          -0.6547595     5.6151118     0.2421063
 43 H  H27          -1.3231583     5.0735728    -1.3017765
 44 H  H28           2.4519754     2.3541053    -1.8218484
 45 C  C3            3.7007781     1.5924446     1.3269725
 46 H  H1            4.1063116     1.0464759     2.1967837
 47 H  H3            3.4754510     2.6186165     1.6585170
 48 H  H6            4.5080077     1.6524125     0.5782286
```

Cartesian Coordinates, calculational information for *trans*-**11b** PM6

Trans-Eb (PM6_TransEb.spartan)

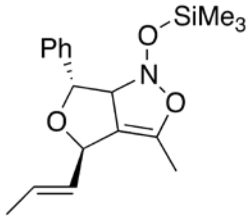


```
Geometry Optimization Method 1
  Initial Hessian option
   Hessian from M3        calculation used.
                                Max.      Max.         Neg.
 Cycle          Energy      Grad.     Dist.        Eigen
     1      -238.4992   0.08477   0.02597

   108      -284.3720   0.00062   0.00039
Heat of Formation:     -284.372 kJ/mol
  Memory Used:          8.428 Mb
  Reason for exit: Successful completion
  Semi-Empirical Program CPU Time :        12.57
  Semi-Empirical Program Wall Time:        12.88
SPARTAN '18 Properties Program:  (Win/64b)                     Release  1.4.0
  Use of molecular symmetry disabled
                     Cartesian Coordinates (Angstroms)
       Atom            X             Y             Z
    ---------    ------------- ------------- -------------

  1 C  C8           -0.0696339     0.3575567    -0.0446785
  2 N  N1            0.4739026    -0.7543604     0.8397541
  3 O  O1            1.6807466    -0.2025472     1.4297542
  4 O  O2           -0.4191744    -0.8198503     1.8935361
  5 C  C1           -0.1971690     0.0330918    -1.5541408
  6 H  H5            0.5413656    -0.7280623    -1.8895679
  7 O  O3            0.1780363     1.2798500    -2.2232893
  8 C  C2            0.9209295     2.1833448    -1.3150631
  9 H  H4            0.2594054     3.0731573    -1.2224933
 10 C  C3            2.2190538     2.4959503    -2.0002091
 11 C  C4            3.0518493     3.4581984    -1.5968373
 12 C  C5            1.0426062     1.3928099    -0.0634427
 13 C  C6            1.9953907     1.0280405     0.8112544
 14 Si Si1          -0.9031760    -2.1645915     2.8482419
 15 C  C7            0.4932043    -2.7147974     3.9326273
 16 H  H12           0.2098167    -3.5938792     4.5298894
 17 H  H14           0.8056814    -1.9137717     4.6207163
 18 H  H15           1.3673537    -2.9813874     3.3148918
 19 C  C9           -1.4755882    -3.5522655     1.7599914
 20 H  H11          -1.8741291    -4.3775045     2.3714745
 21 H  H16          -0.6362976    -3.9412007     1.1607131
 22 H  H17          -2.2671216    -3.2273473     1.0682265
 23 C  C10          -2.2975828    -1.3981843     3.8052493
 24 H  H13          -2.7384953    -2.1421688     4.4874303
 25 H  H18          -3.0875985    -1.0342035     3.1302830
 26 H  H19          -1.9492102    -0.5427058     4.4044427
 27 C  C11          -1.5975920    -0.3207317    -1.9810053
 28 C  C12          -4.1790719    -1.0395970    -2.7857382
```

29	C	C13	-2.3930231	0.5934957	-2.6826960
30	C	C14	-2.0962499	-1.5969876	-1.6786777
31	C	C15	-3.3855438	-1.9519483	-2.0814862
32	C	C16	-3.6813705	0.2317307	-3.0864166
33	H	H2	-1.9902991	1.5824014	-2.9146154
34	H	H9	-1.4807494	-2.3084312	-1.1272840
35	H	H10	-3.7706292	-2.9422433	-1.8483380
36	H	H20	-4.2949411	0.9413617	-3.6382468
37	H	H21	-5.1826629	-1.3191624	-3.0988934
38	H	H1	2.8359838	4.0793699	-0.7259156
39	H	H8	-1.0341818	0.7518081	0.3668233
40	H	H6	2.4133749	1.8687209	-2.8736596
41	C	C17	4.3297510	3.7781128	-2.2950226
42	H	H7	4.2296762	4.7077906	-2.8756740
43	H	H22	4.6376638	2.9942225	-3.0004697
44	H	H23	5.1552377	3.9227750	-1.5863639
45	C	C18	3.2758035	1.5869765	1.2643503
46	H	H3	3.7833933	0.9238984	1.9852253
47	H	H24	3.1389693	2.5643338	1.7534006
48	H	H25	3.9622954	1.7389315	0.4119495

Cartesian Coordinates, calculational information for *cis*-**19a** PM6
only one negative Eigen value
19a.Cis.IR.PM6 IR.PM6_CisGa-UFr

```
Transition State Optimization Method 1
  Initial Hessian option
  Hessian will be calculated numerically
  Estimating Force Constant matrix by central-differences
   Calculating Hessian
                            Max.       Max.         Neg.
 Cycle         Energy       Grad.      Dist.        Eigen
     1         78.6086      0.07780    0.09342        6
   405         53.3652      0.00157    0.00059        2
                            Max.       Max.         Neg.
 Cycle         Energy       Grad.      Dist.        Eigen
     1         53.3652      0.00157    0.00002        1
  Heat of Formation:        53.365 kJ/mol
  Estimating Force Constant matrix by central-differences
   Calculating Hessian
  Memory Used:          22.839 Mb
  Reason for exit: Successful completion
  Semi-Empirical Program CPU Time :        50.44
  Semi-Empirical Program Wall Time:        58.93
SPARTAN '18 Properties Program:  (Win/64b)                    Release  1.4.0
  Use of molecular symmetry disabled
```

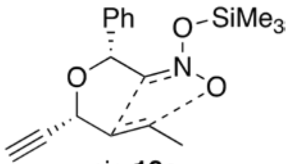

cis-**19a**

Cartesian Coordinates (Angstroms)

Atom			X	Y	Z
1	C	C8	-0.0638226	-0.1313173	-0.5380129
2	N	N1	1.1509412	-0.7014615	-0.3212034
3	O	O1	2.1149845	-0.5024942	-1.0995797
4	O	O2	1.4314046	-1.2624916	0.9046252
5	C	C1	-0.7278853	-0.3209009	-1.8922511
6	H	H5	-0.2428227	-1.1442364	-2.4837390
7	O	O3	-0.5358938	0.8035026	-2.7672468
8	C	C2	-0.1194690	2.0371307	-2.1222160
9	H	H4	0.3119273	2.5741636	-3.0104090

10	C	C3	0.8941074	1.7452442	-1.0588167
11	C	C4	2.1845357	1.3019330	-1.4094193
12	C	C5	-1.2757793	2.7568580	-1.6060864
13	C	C6	-2.2297123	3.3616444	-1.1892937
14	Si	Si1	1.7959437	-1.0803829	2.5353995
15	C	C7	2.1621350	-2.8263977	3.0495545
16	H	H12	1.2765604	-3.4728807	2.9501351
17	H	H14	2.4942221	-2.8524025	4.1004489
18	H	H15	2.9603508	-3.2583202	2.4232732
19	C	C9	0.3131786	-0.3985294	3.4225942
20	H	H11	0.5081725	-0.4237183	4.5100852
21	H	H16	-0.5911612	-0.9946435	3.2279258
22	H	H17	0.1007649	0.6433944	3.1408392
23	C	C10	3.2591991	0.0320800	2.7819985
24	H	H13	3.5985790	-0.0326236	3.8302101
25	H	H18	3.0224949	1.0848157	2.5652865
26	H	H19	4.0985373	-0.2716716	2.1348549
27	C	C11	-2.2087524	-0.6114077	-1.7312702
28	C	C12	-4.9300378	-1.2237373	-1.4870595
29	C	C13	-3.1645211	0.1585706	-2.4054013
30	C	C14	-2.6165808	-1.6963381	-0.9404553
31	C	C15	-3.9748996	-1.9980436	-0.8191838
32	C	C16	-4.5229317	-0.1467754	-2.2804093
33	H	H2	-2.8352236	0.9949459	-3.0276405
34	H	H9	-1.8782025	-2.3072376	-0.4204983
35	H	H10	-4.2902343	-2.8393703	-0.2045877
36	H	H20	-5.2629845	0.4548467	-2.8062739
37	H	H21	-5.9874344	-1.4617602	-1.3919120
38	H	H1	2.4155117	1.1001635	-2.4628119
39	H	H8	-0.7349841	0.0310119	0.3144379
40	H	H6	0.7280483	2.2089276	-0.0901292
41	C	C17	3.3645316	1.6319585	-0.5446940
42	H	H7	3.1089211	1.6727154	0.5231325
43	H	H22	3.7880097	2.6072570	-0.8207849
44	H	H23	4.1609573	0.8809645	-0.6502614
45	H	H26	-3.0507009	3.8770132	-0.8331529

Cartesian Coordinates, calculational information for *trans*-**19a** PM6
only one negative Eigen value

PM6 Trans Ga (IR_PM6_transGa)

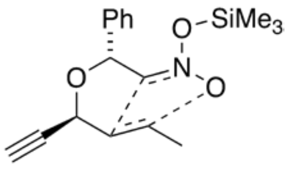

trans-**19a**

```
SPARTAN '18 Semi-Empirical Program:  (Win/64b)           Release  1.0.0
Transition State Optimization Method 1
  Estimating Force Constant matrix by central-differences
   Calculating Hessian
                         Max.      Max.         Neg.
 Cycle        Energy     Grad.     Dist.        Eigen
     1        86.3051   0.09956   0.08972        6
   405        60.0417   0.00066   0.00002
 Cycle        Energy     Grad.     Dist.        Eigen
     1        60.0417   0.00065   0.00000        1
Heat of Formation:       60.042 kJ/mol
  Estimating Force Constant matrix by central-differences
   Calculating Hessian
  Memory Used:         22.839 Mb
  Reason for exit: Successful completion
  Semi-Empirical Program CPU Time :        46.19
  Semi-Empirical Program Wall Time:        46.43
SPARTAN '18 Properties Program:  (Win/64b)                     Release  1.0.0
  Use of molecular symmetry disabled
```

		Cartesian Coordinates (Angstroms)		
	Atom	X	Y	Z
1	C C8	-0.1994185	-0.3338683	0.2389163
2	N N1	-0.4373251	-0.1562847	-1.0875738
3	O O1	-1.5729425	0.1142018	-1.5328748
4	O O2	0.6395163	0.0896473	-1.9145485
5	C C1	-1.0183823	-1.3600279	1.0076903
6	H H5	-1.4604199	-2.1368057	0.3272754
7	O O3	-2.1993007	-0.7993947	1.5935285
8	C C2	-2.0740565	0.6309050	1.9012233
9	C C3	-1.5027954	1.3409257	0.7103613
10	C C4	-2.2426909	1.4611722	-0.4817296
11	Si Si1	1.8328261	0.7588861	-2.8010520
12	C C7	1.8896778	-0.1810259	-4.4013964
13	H H12	0.9297355	-0.1066762	-4.9370528
14	H H14	2.0973197	-1.2491497	-4.2361826
15	H H15	2.6814184	0.2313504	-5.0472792
16	C C9	3.4191503	0.5433144	-1.8547225
17	H H11	3.6067469	-0.5123248	-1.6106284
18	H H16	3.4204736	1.1168474	-0.9175204
19	H H17	4.2539574	0.9034318	-2.4793482
20	C C10	1.5041059	2.5606093	-3.1025963
21	H H13	0.5409431	2.7080907	-3.6152700
22	H H18	2.3000855	2.9688961	-3.7480645
23	H H19	1.4912628	3.1403238	-2.1688037
24	C C11	-0.1700133	-2.0325059	2.0738340
25	C C12	1.3701568	-3.3337230	4.0174880
26	C C13	-0.6097229	-2.0804916	3.4020850
27	C C14	1.0362054	-2.6525891	1.7154950
28	C C15	1.8035722	-3.2987685	2.6876555
29	C C16	0.1618565	-2.7267423	4.3719994
30	H H2	-1.5655613	-1.6218392	3.6678486
31	H H9	1.3763633	-2.6382414	0.6810054
32	H H10	2.7400166	-3.7787461	2.4083360
33	H H20	-0.1831180	-2.7600473	5.4042408
34	H H21	1.9704748	-3.8369132	4.7730459
35	H H1	-3.2347120	0.9946997	-0.5436967
36	H H8	0.8092844	-0.1311731	0.6197851
37	H H6	-0.6891061	2.0298093	0.9140617
38	C C17	-2.0029315	2.6127070	-1.4111565
39	H H7	-0.9415085	2.8861383	-1.4804314
40	H H22	-2.5526998	3.5029169	-1.0760340
41	H H23	-2.3362609	2.3808701	-2.4327547
42	C C18	-3.4280831	1.0286677	2.2439923
43	H H4	-5.5069691	1.6489705	2.7908383
44	C C19	-4.5474929	1.3620024	2.5375877
45	H H24	-1.4007170	0.7119543	2.7924224

Cartesian Coordinates, calculational information for *cis*-**19b** PM6
only one negative Eigen value
PM6 Cis Gb (IR_PM6_Gb.spartan) 4.151 kJ/mol

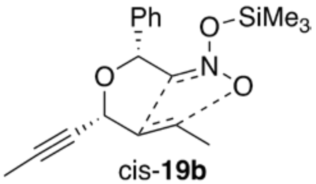

cis-**19b**

```
Transition State Optimization Method 2
  Initial Hessian option
  Hessian will be calculated numerically
  Estimating Force Constant matrix by central-differences
   Calculating Hessian
                          Max.      Max.        Neg.
```

```
 Cycle        Energy    Grad.     Dist.       Eigen
     1        31.9523   0.07022   0.08893       8
   432         4.8466   0.00503   0.00033       2
                        Max.      Max.        Neg.
 Cycle        Energy    Grad.     Dist.       Eigen
     1         4.8466   0.00503   0.00012       1
   284         4.1516   0.00050   0.00000       1
Heat of Formation:        4.152 kJ/mol
  Estimating Force Constant matrix by central-differences
   Calculating Hessian
  Memory Used:         25.311 Mb
  Reason for exit: Successful completion
  Semi-Empirical Program CPU Time :      1:27.24
  Semi-Empirical Program Wall Time:      1:27.69
SPARTAN '18 Properties Program:  (Win/64b)                    Release  1.0.0
  Use of molecular symmetry disabled

                   Cartesian Coordinates (Angstroms)
     Atom            X             Y             Z
   ---------    ------------- ------------- -------------
  1 C  C8         -0.5131991    -0.3690918    -0.2661018
  2 N  N1         -0.7488696    -0.2076507    -1.5942061
  3 O  O1         -1.8846794     0.1249580    -2.0161349
  4 O  O2          0.3089881    -0.1310631    -2.4782899
  5 C  C1         -1.4326981    -1.2732493     0.5392280
  6 H  H5         -2.0831637    -1.9045685    -0.1235118
  7 O  O3         -2.4139294    -0.5391129     1.2879676
  8 C  C2         -2.1457835     0.8823945     1.4473317
  9 H  H4         -3.1819405     1.2367010     1.6965834
 10 C  C3         -1.6218908     1.4469358     0.1611976
 11 C  C4         -2.4518235     1.5236230    -0.9730940
 12 C  C5         -1.2463078     1.1093089     2.5649741
 13 C  C6         -0.5068743     1.2896751     3.5011068
 14 Si Si1         1.5636985     0.9417540    -2.9254004
 15 C  C7          2.5486969    -0.1046351    -4.0968777
 16 H  H12         1.9139080    -0.4798071    -4.9167327
 17 H  H14         2.9878115    -0.9785312    -3.5917848
 18 H  H15         3.3656358     0.4889857    -4.5376080
 19 C  C9          2.5324748     1.4195069    -1.4153360
 20 H  H11         2.9473363     0.5402403    -0.8998731
 21 H  H16         1.9205827     1.9820283    -0.6939133
 22 H  H17         3.3775722     2.0629357    -1.7144065
 23 C  C10         0.8555609     2.4482479    -3.7403203
 24 H  H13         0.3896543     2.1881759    -4.7047297
 25 H  H18         1.6373265     3.2001415    -3.9266285
 26 H  H19         0.0716112     2.9115411    -3.1083562
 27 C  C11        -0.6263350    -2.1642834     1.4666774
 28 C  C12         0.8250367    -3.8751020     3.1420178
 29 C  C13        -0.8743625    -2.1740978     2.8440493
 30 C  C14         0.3464537    -3.0187956     0.9259658
 31 C  C15         1.0703715    -3.8695483     1.7645048
 32 C  C16        -0.1480875    -3.0275884     3.6795359
 33 H  H2         -1.6411391    -1.5090522     3.2520882
 34 H  H9          0.5378651    -3.0237397    -0.1461154
 35 H  H10         1.8249664    -4.5314510     1.3441021
 36 H  H20        -0.3454224    -3.0335412     4.7497407
 37 H  H21         1.3884803    -4.5408523     3.7922768
 38 H  H1         -3.4533318     1.0763794    -0.9300183
 39 H  H8          0.5103571    -0.2604394     0.1114711
```

```
 40 H  H6          -0.7496615     2.0897545     0.2354139
 41 C  C17         -2.2874708     2.6270704    -1.9745213
 42 H  H7          -1.2280102     2.8437692    -2.2001061
 43 H  H22         -2.7442753     3.5558988    -1.6080775
 44 H  H23         -2.7644365     2.3725586    -2.9317770
 45 C  C18          0.3696541     1.5018819     4.6142186
 46 H  H3           1.0536618     2.3503305     4.4388937
 47 H  H24          0.9941323     0.6112278     4.8089809
 48 H  H25         -0.1922499     1.7174776     5.5399369
```

Cartesian Coordinates, calculational information for *trans*-**19b** PM6
only one negative Eigen value

```
Transition State Optimization Method 2
```

Trans 19b Trans19b.IR2.PM6.spartan DuffyGroup – 11.2024 – GoodPM6

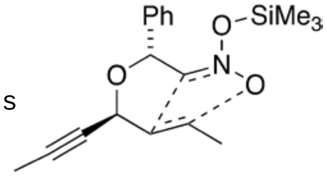

trans-**19b**

```
SPARTAN'24 Semi-Empirical Program:  (Win/64b)            Release  1.2.0
   Calculating Hessian
                            Max.      Max.        Neg.
 Cycle        Energy    Grad.     Dist.       Eigen
     1        32.5644   0.12631   0.06391       1
    25        14.8402   0.00020   0.00040       1
M0001
  Heat of Formation:       14.840 kJ/mol
  Estimating Force Constant matrix by central-differences
   Calculating Hessian
  Memory Used:         25.311 Mb
  Reason for exit: Successful completion
  Semi-Empirical Program CPU Time :        21.78
  Semi-Empirical Program Wall Time:        21.81
SPARTAN'24 Properties Program:  (Win/64b)                      Release  1.2.0
  Use of molecular symmetry disabled

                     Cartesian Coordinates (Angstroms)
       Atom            X             Y             Z
    ---------    ------------- ------------- -------------

  1 C  C8           0.1502384    -0.6100413    -0.1245845
  2 N  N1          -0.0808860    -0.4072692    -1.4473531
  3 O  O1          -1.2078318    -0.0490806    -1.8681888
  4 O  O2           0.9850960    -0.3165554    -2.3224204
  5 C  C1          -0.7638103    -1.5418468     0.6578210
  6 H  H1_1        -1.3423968    -2.2244564    -0.0201772
  7 O  O3          -1.8188874    -0.8523165     1.3371071
  8 C  C2          -1.5186651     0.5611678     1.6055009
  9 C  C3          -0.9670713     1.1881291     0.3590174
 10 C  C4          -1.7676783     1.3229567    -0.7916376
 11 Si Si1          2.2563633     0.7403583    -2.7468395
 12 C  C7           3.2031668    -0.2809070    -3.9698992
 13 H  H7a          2.5501849    -0.6116189    -4.7945563
 14 H  H7c          3.6294651    -1.1824378    -3.5036356
 15 H  H7f          4.0272987     0.3117576    -4.3986741
 16 C  C9           3.2576520     1.1377919    -1.2348811
 17 H  H9a          3.6419511     0.2304100    -0.7463071
 18 H  H9c          2.6783479     1.7073201    -0.4937731
 19 H  H9f          4.1252735     1.7529188    -1.5294805
 20 C  C10          1.5762074     2.2937506    -3.4927110
 21 H  H10b         1.0751324     2.0802285    -4.4507342
 22 H  H10c         2.3780046     3.0244585    -3.6787876
 23 H  H10f         0.8268418     2.7616316    -2.8242838
```

```
 24 C  C11          0.0442869    -2.3618720     1.6500819
 25 C  C12          1.5072914    -3.9260487     3.4540605
 26 C  C13         -0.2912494    -2.3650166     3.0090921
 27 C  C14          1.1066616    -3.1550591     1.1915759
 28 C  C15          1.8359250    -3.9328444     2.0940472
 29 C  C16          0.4419324    -3.1436229     3.9090708
 30 H  H13         -1.1378235    -1.7647562     3.3525744
 31 H  H14          1.3628973    -3.1710819     0.1331419
 32 H  H15          2.6600217    -4.5478119     1.7369798
 33 H  H16          0.1783981    -3.1424851     4.9653288
 34 H  H12          2.0772515    -4.5324320     4.1549800
 35 H  H1          -2.7783202     0.8914106    -0.7816855
 36 H  H8           1.1767843    -0.5156538     0.2494403
 37 H  H6          -0.0989594     1.8238738     0.4925039
 38 C  C17         -1.5616833     2.4601775    -1.7462656
 39 H  H17b        -0.4948320     2.6743095    -1.9329951
 40 H  H17c        -2.0205531     3.3787735    -1.3567611
 41 H  H17f        -2.0145803     2.2478878    -2.7250055
 42 C  C19         -2.7884724     1.1073672     2.0399047
 43 H  H2_2        -0.7732903     0.5772083     2.4401101
 44 C  C5          -3.8490363     1.5527938     2.4042908
 45 C  C6          -5.1022641     2.0901374     2.8431835
 46 H  H6c         -4.9651318     2.8711826     3.6112957
 47 H  H6b         -5.7438154     1.3048698     3.2829130
 48 H  H6a         -5.6654353     2.5423427     2.0076160

  Point Group = C1  Order =  1 Nsymop =  1
  Temperature Corrections for   298.15 K
  Reason for exit: Successful completion
  Properties CPU Time :        0.516
  Properties Wall Time:        0.493
```

Cartesian Coordinates, calculational information for *cis*-**20a** PM6
only one negative Eigen value
Cis 20a Good run PM6 IR one negative peak IR.PM6_Da

```
Transition State Optimization Method 2
   Calculating Hessian
                     Max.      Max.        Neg.
 Cycle        Energy   Grad.     Dist.       Eigen
     1        72.9376   0.00035   0.00000       1
M0001
  Heat of Formation:       72.938 kJ/mol
  Estimating Force Constant matrix by central-difference
   Calculating Hessian
  Memory Used:         22.839 Mb
  Reason for exit: Successful completion
  Semi-Empirical Program CPU Time :        19.63
  Semi-Empirical Program Wall Time:        19.81
SPARTAN'24 Properties Program:  (Win/64b)                    Release  1.2.0
  Use of molecular symmetry disabled
```

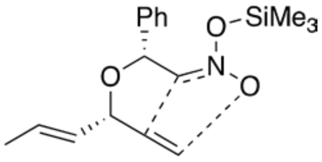

cis-**20a**

```
                     Cartesian Coordinates (Angstroms)
       Atom            X             Y             Z
    ---------    ------------- ------------- -------------
  1 C  C8           0.6600641    -0.0914130    -0.5282175
  2 N  N1           1.3505344    -0.9730710     0.2553653
  3 O  O1           2.6024797    -0.9069139     0.3609305
  4 O  O2           0.6861042    -1.3793840     1.4031659
```

```
  5 C  C1          0.8983761    0.0450094   -2.0174743
  6 H  H5          1.8693509   -0.4008957   -2.3440690
  7 O  O3          0.9858529    1.4632620   -2.3176326
  8 C  C2          1.5208789    2.2821966   -1.2210413
  9 C  C5          2.2380634    1.4099581   -0.3127834
 10 H  H3          4.0018289    0.5941523    0.8356445
 11 C  C6          3.1194799    0.7761834    0.3003063
 12 Si Si1        -0.0035297   -1.8069131    2.7960184
 13 C  C7          0.9059796   -3.2672350    3.4924466
 14 H  H12         0.5045984   -3.5145287    4.4881960
 15 H  H14         1.9806938   -3.0515574    3.5946026
 16 H  H15         0.8013315   -4.1512630    2.8458215
 17 C  C9         -1.7670657   -2.2582672    2.4156469
 18 H  H11        -2.2340403   -2.6654965    3.3279215
 19 H  H16        -1.8268124   -3.0234477    1.6279648
 20 H  H17        -2.3502200   -1.3863414    2.0874948
 21 C  C10         0.0563125   -0.3779242    3.9807345
 22 H  H13        -0.4902175   -0.6477588    4.8990694
 23 H  H18        -0.4061062    0.5239507    3.5563946
 24 H  H19         1.0937617   -0.1324681    4.2533049
 25 C  C11        -0.2567481   -0.5074313   -2.8233442
 26 C  C12        -2.3600996   -1.5818713   -4.3220990
 27 C  C13        -1.0946372    0.3383914   -3.5593725
 28 C  C14        -0.4700371   -1.8942862   -2.8374678
 29 C  C15        -1.5221039   -2.4269854   -3.5856835
 30 C  C16        -2.1447757   -0.2008508   -4.3084988
 31 H  H2         -0.9131784    1.4162532   -3.5467365
 32 H  H9          0.1792738   -2.5562938   -2.2663079
 33 H  H10        -1.6892474   -3.5027805   -3.5962821
 34 H  H20        -2.7936670    0.4580194   -4.8832208
 35 H  H21        -3.1777196   -2.0007631   -4.9058196
 36 H  H8         -0.3336458    0.2415048   -0.1908238
 37 C  C18         0.3718506    3.0355641   -0.6053441
 38 H  H24        -0.5497065    2.9699371   -1.1855947
 39 C  C19         0.4724239    3.7351364    0.5271005
 40 H  H25         1.4065632    3.7788550    1.0927528
 41 C  C20        -0.6548397    4.5063507    1.1232364
 42 H  H4         -0.4133738    5.5779852    1.1783371
 43 H  H26        -0.8649412    4.1669824    2.1479904
 44 H  H27        -1.5894653    4.4142317    0.5542136
 45 H  H28         2.2006426    2.9722169   -1.7868468
```

Cartesian Coordinates, calculational information for *trans*-**20a** PM6 only one negative Eigen value

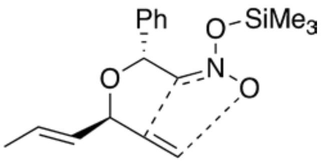

trans-**20a**

PM6 for Trans Da (PM6_TransDa_IR2.spartan)

```
Transition State Optimization Method 2
  Initial Hessian option
  Hessian will be calculated numerically
  Estimating Force Constant matrix by central-differences
   Calculating Hessian
                          Max.      Max.        Neg.
 Cycle        Energy     Grad.     Dist.       Eigen
     1      120.0309   0.09233   0.05058        7
   405       83.4046   0.00496   0.00043        2
                          Max.      Max.        Neg.
 Cycle        Energy     Grad.     Dist.       Eigen
     1       83.4046   0.00496   0.00018        1
   282       84.3429   0.00062   0.00000        1
Heat of Formation:       84.343 kJ/mol
```

```
  Estimating Force Constant matrix by central-differences
   Calculating Hessian
  Memory Used:         22.839 Mb
  Reason for exit: Successful completion
  Semi-Empirical Program CPU Time :      1:14.55
  Semi-Empirical Program Wall Time:      1:14.93
SPARTAN '18 Properties Program:  (Win/64b)                    Release  1.0.0
  Use of molecular symmetry disabled

                    Cartesian Coordinates (Angstroms)
       Atom            X             Y             Z
    ---------    ------------- ------------- -------------
   1 C  C8         -0.4624016    -0.3122781    -0.6687901
   2 N  N1         -1.6126741    -0.7075324    -0.0539094
   3 O  O1         -2.0312293    -1.9059584    -0.1924500
   4 O  O2         -2.0951641     0.0119301     1.0147578
   5 C  C1         -0.2075903    -0.4472128    -2.1579994
   6 H  H5         -0.9125852    -1.1550280    -2.6569239
   7 O  O3          1.1219304    -1.0124110    -2.3211776
   8 C  C2          1.5217178    -1.9131979    -1.2263879
   9 C  C5          0.3149479    -2.2992027    -0.5270049
  10 H  H3         -1.2046189    -3.7980016     0.1904906
  11 C  C6         -0.6952179    -2.9450404    -0.1503036
  12 Si Si1        -1.7205398     0.0824662     2.7068508
  13 C  C7          0.0884002     0.4381166     2.9107866
  14 H  H12         0.3684502     1.4043756     2.4642941
  15 H  H14         0.7136767    -0.3416733     2.4457625
  16 H  H15         0.3383094     0.4765369     3.9841560
  17 C  C9         -2.1682140    -1.5220580     3.5147571
  18 H  H11        -1.9726286    -1.4847170     4.5977605
  19 H  H16        -1.5920184    -2.3599662     3.0905374
  20 H  H17        -3.2374708    -1.7494203     3.3666087
  21 C  C10        -2.8018444     1.4884774     3.2445510
  22 H  H13        -3.8356765     1.3529605     2.8834515
  23 H  H18        -2.4376522     2.4505782     2.8503196
  24 H  H19        -2.8275415     1.5502425     4.3446279
  25 C  C11        -0.2028588     0.8988154    -2.8482224
  26 C  C12        -0.2545222     3.3762542    -4.1470536
  27 C  C13         0.9592077     1.3995789    -3.4470341
  28 C  C14        -1.3941679     1.6386566    -2.8998003
  29 C  C15        -1.4159729     2.8753653    -3.5481287
  30 C  C16         0.9311192     2.6374770    -4.0960210
  31 H  H2          1.8796450     0.8114452    -3.4050148
  32 H  H9         -2.3002768     1.2531328    -2.4333336
  33 H  H10        -2.3397687     3.4500399    -3.5876019
  34 H  H20         1.8350194     3.0239561    -4.5633565
  35 H  H21        -0.2756657     4.3393118    -4.6530902
  36 H  H8          0.1421700     0.4690305    -0.1826082
  37 C  C18         2.5471029    -1.1912053    -0.3929612
  38 H  H24         2.9377810    -0.2925253    -0.8730090
  39 C  C19         2.9549172    -1.6136443     0.8057961
  40 H  H25         2.5418940    -2.5138015     1.2668875
  41 C  C20         3.9920934    -0.9150476     1.6172749
  42 H  H4          4.8503521    -1.5749181     1.8115325
  43 H  H26         3.5886353    -0.6080679     2.5929651
  44 H  H27         4.3835345    -0.0118728     1.1297282
  45 H  H28         1.9873884    -2.7541496    -1.8017149
```

Cartesian Coordinates, calculational information for *cis*-**20b** PM6
only one negative Eigen value
PM6_CisDb
Transition State Optimization Method 1
PM6_TransDb_UFr.spartan (SpartanRes->DuffySum2019->Calc->NewCD)
Initial Hessian option
Hessian will be calculated numerically
Estimating Force Constant matrix by central-differences
Calculating Hessian

Cycle	Energy	Max. Grad.	Max. Dist.	Neg. Eigen
1	90.1968	0.15418	0.05904	9
432	17.2677	0.00164	0.02948	2
1	17.2677	0.00162	0.00002	1

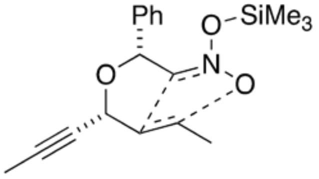

cis-**20b**

Heat of Formation: 17.268 kJ/mol
Estimating Force Constant matrix by central-differences
Calculating Hessian
Memory Used: 25.311 Mb
Reason for exit: Successful completion
Semi-Empirical Program CPU Time : 56.49
Semi-Empirical Program Wall Time: 1:00.00

SPARTAN '18 Properties Program: (Win/64b) Release 1.4.0
Use of molecular symmetry disabled

Cartesian Coordinates (Angstroms)

	Atom		X	Y	Z
1	C	C8	-1.1454084	-0.2032395	0.2388899
2	N	N1	-0.5257824	0.5217354	1.1306514
3	O	O1	0.9919888	1.1127362	0.7001710
4	O	O2	-1.3628708	1.0843850	2.0473203
5	C	C1	-0.3018131	-0.8864851	-0.8235344
6	H	H5	0.6525655	-1.2708965	-0.3856331
7	O	O3	-0.0019061	0.1004222	-1.8489438
8	C	C21	1.3604645	0.5934141	-1.8822057
9	C	C5	1.7371109	1.1950270	-0.5976057
10	C	C6	2.3354363	1.8501729	0.3388657
11	Si	Si1	-1.2547285	2.1693294	3.3413081
12	C	C7	-0.8328198	3.8614653	2.7078639
13	H	H12	-0.6890221	4.5739912	3.5338260
14	H	H14	-1.6228872	4.2491708	2.0453814
15	H	H15	0.0986537	3.8092983	2.1182172
16	C	C9	0.0051193	1.5969407	4.5757365
17	H	H11	0.1148599	2.3252216	5.3932847
18	H	H16	0.9816854	1.4791297	4.0757157
19	H	H17	-0.2662849	0.6232615	5.0118154
20	C	C10	-2.9852121	2.0856596	4.0157470
21	H	H13	-3.7295837	2.3597959	3.2516807
22	H	H18	-3.0778484	2.7876481	4.8599568
23	H	H19	-3.2286558	1.0739513	4.3749743
24	C	C11	-1.0818646	-1.9895155	-1.5059529
25	C	C12	-2.5106791	-4.0460075	-2.7537121
26	C	C13	-1.0289870	-3.2914907	-0.9901051
27	C	C14	-1.8502983	-1.7173901	-2.6462469
28	C	C15	-2.5620332	-2.7463757	-3.2685329
29	C	C16	-1.7447505	-4.3163529	-1.6150156
30	H	H2	-0.4358274	-3.5067910	-0.1024509

```
 31 H  H9          -1.8777122    -0.6992502    -3.0418438
 32 H  H10         -3.1579419    -2.5344406    -4.1544955
 33 H  H20         -1.7051982    -5.3265719    -1.2143009
 34 H  H21         -3.0667398    -4.8463918    -3.2379326
 35 H  H8          -2.2292167    -0.3537762     0.1934339
 36 C  C18          2.3255089    -0.4854791    -2.3095507
 37 H  H24          3.2840114    -0.4893812    -1.7948177
 38 C  C19          2.0127744    -1.3697450    -3.2624049
 39 H  H25          1.0321470    -1.3477470    -3.7532068
 40 C  C20          2.9299032    -2.4470790    -3.7293174
 41 H  H4           3.2784875    -2.2466007    -4.7540137
 42 H  H26          2.4201574    -3.4216081    -3.7454732
 43 H  H27          3.8227223    -2.5591171    -3.0985648
 44 H  H23          1.2552294     1.3832225    -2.6771233
 45 C  C2           3.2265428     2.6499501     1.1347310
 46 H  H1           3.7790383     2.0275997     1.8627897
 47 H  H3           2.6658126     3.4013430     1.7222031
 48 H  H6           3.9658601     3.1868613     0.5184206
```

Cartesian Coordinates, calculational information for *trans*-**20b** PM6
only one negative Eigen value
Spartan 18 PM6 Trans-Db (PM6_TransDb_IR.spartan)

```
SPARTAN '18 Semi-Empirical Program:  (Win/64b)           Release  1.0.0
Transition State Optimization Method 2
  Initial Hessian option
  Hessian will be calculated numerically
  Estimating Force Constant matrix by central-differences
   Calculating Hessian
                     Max.      Max.        Neg.
 Cycle        Energy    Grad.     Dist.       Eigen
     1        79.9960   0.07809   0.05259       7
   432        47.5086   0.00098   0.00015       2
                      Max.      Max.        Neg.
 Cycle        Energy    Grad.     Dist.       Eigen
     1        47.5086   0.00098   0.00001       1
  Heat of Formation:       47.509 kJ/mol
  Estimating Force Constant matrix by central-differences
   Calculating Hessian
  Memory Used:         25.311 Mb
  Reason for exit: Successful completion
  Semi-Empirical Program CPU Time :        54.40
  Semi-Empirical Program Wall Time:        54.63
SPARTAN '18 Properties Program:  (Win/64b)                   Release  1.0.0
  Temperature Corrections for   298.15 K
  Reason for exit: Successful completion
  Properties CPU Time :          .70
  Properties Wall Time:          .72
SPARTAN '18 Properties Program:  (Win/64b)                   Release  1.0.0
  Use of molecular symmetry disabled
```

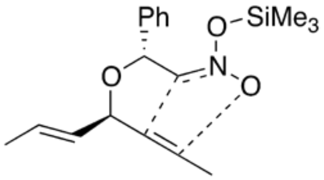


```
                     Cartesian Coordinates (Angstroms)
       Atom            X             Y             Z
    ---------    ------------- ------------- -------------
  1 C  C8          -0.4903433    -0.3361995    -0.2537647
  2 N  N1           0.1493478    -1.3846340     0.3347881
  3 O  O1           1.4175298    -1.4785889     0.2599828
  4 O  O2          -0.4843137    -2.0988544     1.3294646
```

5	C	C1	-0.3367628	0.0451728	-1.7190084
6	H	H5	0.6006829	-0.3518532	-2.1758487
7	O	O3	-0.2631864	1.4926616	-1.7289471
8	C	C2	0.6033976	2.0290569	-0.6563086
9	C	C5	1.1789642	0.9163505	0.0687704
10	C	C6	2.0615336	0.1210386	0.4884343
11	Si	Si1	-0.5582041	-1.9352784	3.0564499
12	C	C7	1.1656542	-1.8531069	3.7363748
13	H	H12	1.1879043	-1.3502638	4.7143686
14	H	H14	1.8388210	-1.3026081	3.0507160
15	H	H15	1.5830706	-2.8656792	3.8568022
16	C	C9	-1.4600969	-3.4862097	3.5215970
17	H	H11	-1.5119142	-3.5888963	4.6173825
18	H	H16	-0.9440711	-4.3729341	3.1152094
19	H	H17	-2.4877700	-3.4896621	3.1251831
20	C	C10	-1.5149853	-0.4042191	3.4831140
21	H	H13	-2.4998342	-0.3912676	2.9912054
22	H	H18	-0.9791576	0.5128670	3.1923830
23	H	H19	-1.6828428	-0.3660517	4.5732585
24	C	C11	-1.5543809	-0.3350098	-2.5299503
25	C	C12	-3.7782709	-1.0881137	-4.0507245
26	C	C13	-2.4183549	0.6426629	-3.0383234
27	C	C14	-1.8028061	-1.6928134	-2.7829638
28	C	C15	-2.9145078	-2.0646803	-3.5422178
29	C	C16	-3.5286237	0.2640292	-3.7987266
30	H	H2	-2.2110408	1.6971675	-2.8389558
31	H	H9	-1.1341734	-2.4563628	-2.3865133
32	H	H10	-3.1088244	-3.1182936	-3.7374848
33	H	H20	-4.1977392	1.0254085	-4.1953740
34	H	H21	-4.6426894	-1.3815258	-4.6429459
35	H	H8	-1.4340077	0.0097844	0.1956997
36	C	C3	1.6307309	2.8714990	-1.3689839
37	H	H6	1.7610039	2.6120363	-2.4202826
38	C	C4	2.3076165	3.8522968	-0.7677308
39	H	H7	2.1556213	4.0951307	0.2855065
40	C	C17	3.3192676	4.6991771	-1.4625010
41	H	H1	4.2704340	4.7139585	-0.9133576
42	H	H22	2.9696428	5.7390619	-1.5416749
43	H	H23	3.5381217	4.3542492	-2.4823315
44	H	H28	-0.1013363	2.6408862	-0.0442733
45	C	C18	3.3182512	-0.2095143	1.1347476
46	H	H3	3.9568261	0.6792141	1.2567991
47	H	H4	3.8869700	-0.9577482	0.5545647
48	H	H24	3.1386221	-0.6533417	2.1363922

Cartesian Coordinates, calculational information for **I** c*is*-**9a**.RRRRS.PM6

Calculated already on page 40PM6 cis Ha (PM6_cisHa.spartan)

3R,3aR,4R,6R, 6aS

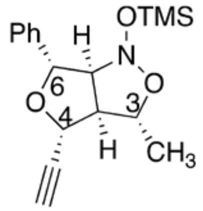

Geometry Optimization Method 2

Hessian from M3 calculation used.

Cycle	Energy	Max. Grad.	Max. Dist.	Neg. Eigen
1	-168.7895	0.10159	0.02710	
99	-210.2551	0.00045	0.00010	

Heat of Formation: -210.255 kJ/mol

Cartesian Coordinates, calculational information for **II** *cis*-**9a**.SSRRR.PM6
cisII.SSRRR.PM6 (SpartanResearch->10.2024)

3S,3aS,4R,6R,6aR

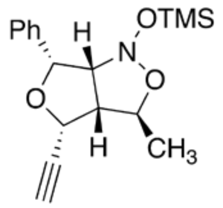


```
Geometry Optimization Method 2
  Initial Hessian option
   Hessian from M3        calculation used.
                         Max.      Max.        Neg.
 Cycle        Energy     Grad.     Dist.       Eigen
     1      -147.1728   0.09723   0.03536
   139      -189.1106   0.00058   0.00028
  Heat of Formation:     -189.111 kJ/mol
  Memory Used:           7.604 Mb
  Reason for exit: Successful completion
  Semi-Empirical Program CPU Time :          6.09
  Semi-Empirical Program Wall Time:          6.02
SPARTAN'24 Properties Program:  (Win/64b)                     Release  1.2.0
  Use of molecular symmetry disabled
```

Cartesian Coordinates (Angstroms)

	Atom		X	Y	Z
1	O	O1	-0.5540777	1.3179087	-2.9334436
2	C	C3	0.4405691	0.7940124	-2.0117748
3	C	C4	-0.1167289	1.1042997	-0.5967879
4	C	C5	-1.5904938	1.5683384	-0.7959130
5	C	C6	-1.8632792	1.3958390	-2.3120797
6	H	H6	1.3251422	1.4392491	-2.2394643
7	H	H8	0.5033717	1.8999378	-0.1134540
8	H	H10	-1.7023338	2.6418805	-0.5299062
9	H	H12	-2.2917229	2.3214099	-2.7742911
10	N	N1	-0.1668718	-0.1193673	0.3007972
11	O	O2	-1.4863463	-0.4338938	0.5049623
12	O	O3	0.3509397	0.3071642	1.5284539
13	Si	Si1	1.2584189	0.3353046	2.8515974
14	C	C1	2.9890503	0.8430439	2.3891590
15	H	H1	3.0580444	1.9170616	2.1718126
16	H	H7	3.6583686	0.6190555	3.2361984
17	H	H13	3.3520701	0.2886708	1.5125030
18	C	C2	0.5109574	1.6054607	3.9910490
19	H	H3	0.5346960	2.6149808	3.5624441
20	H	H14	1.0979229	1.6114056	4.9248587
21	H	H15	-0.5305107	1.3583578	4.2398084
22	C	C7	1.2791502	-1.3299794	3.6671506
23	H	H4	0.2717584	-1.6121451	4.0100426
24	H	H16	1.9556602	-1.3239906	4.5349093
25	H	H17	1.6188477	-2.1049133	2.9614583
26	C	C8	-2.4028246	0.6811476	0.1621409
27	H	H19	-3.2508822	0.1435188	-0.3119933
28	C	C9	0.7762325	-0.6423380	-2.3319852
29	C	C10	1.4844427	-3.2746024	-2.9759983
30	C	C11	1.7236362	-1.3263042	-1.5545296
31	C	C12	0.2007844	-1.2736072	-3.4416680
32	C	C13	0.5540957	-2.5876682	-3.7611522
33	C	C14	2.0689332	-2.6415761	-1.8737434
34	H	H2	1.7528691	-4.3001966	-3.2197613
35	H	H5	2.1753835	-0.8447117	-0.6866354
36	H	H9	-0.5233531	-0.7290712	-4.0520066
37	H	H20	0.1028168	-3.0746782	-4.6230796
38	H	H21	2.7903224	-3.1754504	-1.2579185
39	C	C15	-2.6542224	0.2366232	-2.6769554

```
 40 C  C16          -3.3028315    -0.7207434    -3.0141313
 41 H  H25          -3.8560276    -1.5476691    -3.2974153
 42 C  C17          -2.8093397     1.3771047     1.4459175
 43 H  H11          -3.4483782     2.2466052     1.2657076
 44 H  H18          -3.3560709     0.6880629     2.1053865
 45 H  H22          -1.9282024     1.7064632     2.0197308
```

Cartesian Coordinates, calculational information for **III** *cis*-**9a**.SSRRS.PM6

cisIII 9aSRRRS PM6 (DuffyGroup->2024)

3S,3aS,4R,6R,6aS

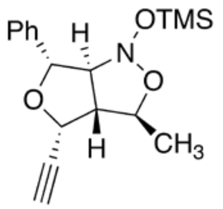

```
Geometry Optimization Method 2
  Initial Hessian option
   Hessian from MMFF94     calculation used.
                          Max.      Max.        Neg.
 Cycle        Energy      Grad.     Dist.       Eigen
     1       -58.5026    0.10744   0.03636
    84      -132.6320    0.00030   0.00010
  Heat of Formation:      -132.632 kJ/mol
  Memory Used:            7.604 Mb
  Reason for exit: Successful completion
  Semi-Empirical Program CPU Time :          3.64
  Semi-Empirical Program Wall Time:          3.61
SPARTAN'24 Properties Program:  (Win/64b)                     Release  1.2.0
  Use of molecular symmetry disabled

                     Cartesian Coordinates (Angstroms)
       Atom           X             Y             Z
    ---------    ------------- ------------- -------------
  1 H  H1           -0.3188162    -1.4285903    -0.3366804
  2 C  C1           -0.9817061    -0.5914171    -0.6520183
  3 O  O1           -0.9975212    -0.6610958    -2.1297017
  4 C  C4           -0.2917630     0.4799841    -2.7386841
  5 H  H4            0.2545741     0.0074312    -3.5886857
  6 C  C5           -2.3845610    -0.7637079    -0.1360168
  7 C  C7           -4.9657368    -1.1125739     0.8791736
  8 C  C8           -2.5913450    -0.8012148     1.2516181
  9 C  C9           -3.4670874    -0.9003758    -1.0124656
 10 C  C10          -4.7569836    -1.0752201    -0.5023898
 11 C  C11          -3.8822328    -0.9747302     1.7547007
 12 H  H8           -1.7468119    -0.6901806     1.9338542
 13 H  H9           -3.2902094    -0.8715864    -2.0906295
 14 H  H10          -5.5986947    -1.1829901    -1.1843038
 15 H  H11          -4.0452762    -1.0024740     2.8307556
 16 H  H7           -5.9703184    -1.2490410     1.2752151
 17 C  C12          -1.2463949     1.4654817    -3.2020159
 18 H  H13_1        -2.7377916     2.9609789    -3.9440987
 19 C  C13          -2.0491567     2.2698828    -3.6003133
 20 C  C2           -0.3912533     0.8002728    -0.3924790
 21 H  H2           -1.2310321     1.5534583    -0.4526299
 22 C  C3            0.6057717     0.9412144    -1.5875488
 23 H  H3            1.4256960     0.1737126    -1.4907099
 24 N  N1            0.4211345     1.2720447     0.7833973
 25 O  O2            1.1685278     2.3181606     0.2339457
 26 C  C6            1.2109958     2.3047568    -1.2797118
 27 H  H6_1          0.5749459     3.1613229    -1.5775170
 28 C  C14           2.6622056     2.4849443    -1.6550846
 29 H  H14c          3.0486601     3.4448720    -1.2823352
 30 H  H14b          2.8074339     2.4616849    -2.7412494
 31 H  H14a          3.2991544     1.7072420    -1.2068398
 32 O  O3            1.3087865     0.2791541     1.1442328
```

```
 33 Si  Si1          2.2476225    -0.8309022     1.7998355
 34 C   C15          3.4387892    -0.0377474     2.9799446
 35 H   H15c         4.0970793    -0.8014464     3.4221299
 36 H   H15b         2.8993222     0.4720703     3.7936435
 37 H   H15a         4.0626298     0.7139950     2.4745503
 38 C   C16          3.1745637    -1.6731730     0.4203517
 39 H   H16c         3.8336311    -2.4358590     0.8688588
 40 H   H16b         3.8009965    -0.9643429    -0.1371642
 41 H   H16a         2.5032894    -2.1758593    -0.2877055
 42 C   C17          1.1825507    -2.0656764     2.6929876
 43 H   H17c         1.8354742    -2.8197590     3.1631771
 44 H   H17b         0.4858112    -2.5848816     2.0215559
 45 H   H17a         0.5950467    -1.5778193     3.4850506
```

Cartesian Coordinates, calculational information for **IV** *cis*-**9a**.RRRRR.PM6
cisIV.9aRRRRR.PM6 (Duffygroup->2024)

3R,3aR,4R,6R,6aR

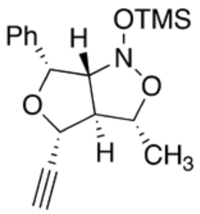


```
PM6 Geometry Optimization Method 2
  Initial Hessian option
   Hessian from MMFF94     calculation used.
                            Max.      Max.        Neg.
 Cycle        Energy      Grad.     Dist.       Eigen
     1       -33.2413    0.13492   0.03342
    87      -117.2204    0.00012   0.00006
  Heat of Formation:      -117.220 kJ/mol
  Memory Used:            7.604 Mb
  Reason for exit: Successful completion
  Semi-Empirical Program CPU Time :          3.97
  Semi-Empirical Program Wall Time:          4.08
SPARTAN'24 Properties Program:  (Win/64b)                      Release  1.2.0
  Use of molecular symmetry disabled

                     Cartesian Coordinates (Angstroms)
       Atom            X             Y             Z
    ---------    ------------- ------------- -------------
  1 H   H1          -0.5910295    -2.1951621    -1.3020788
  2 C   C1          -0.7485892    -1.0951665    -1.3543875
  3 O   O1          -0.6827360    -0.8319054    -2.8036031
  4 C   C4           0.0042375     0.4498420    -3.0948186
  5 H   H4           0.9266959     0.1497609    -3.6441623
  6 C   C5          -2.0924215    -0.7127045    -0.7913377
  7 C   C7          -4.6088715    -0.0816966     0.2569979
  8 C   C8          -2.3067962    -0.7629780     0.5932312
  9 C   C9          -3.1384923    -0.3478009    -1.6489374
 10 C   C10         -4.3942232    -0.0324973    -1.1238677
 11 C   C11         -3.5643372    -0.4460611     1.1132780
 12 H   H8          -1.4914513    -1.0353972     1.2659658
 13 H   H9          -2.9590044    -0.3149954    -2.7267815
 14 H   H10         -5.2055062     0.2501444    -1.7919107
 15 H   H11         -3.7291106    -0.4808484     2.1886750
 16 H   H7          -5.5871774     0.1633039     0.6656236
 17 C   C12         -0.8765437     1.2452305    -3.9212622
 18 H   H13_1       -2.2415903     2.4544315    -5.2193904
 19 C   C13         -1.6122513     1.8950916    -4.6190751
 20 C   C2           0.4578613    -0.2711815    -0.8785337
 21 H   H2           1.3805761    -0.8366463    -1.2071709
 22 C   C3           0.2553623     1.0342345    -1.7025582
 23 H   H3          -0.6936138     1.5500358    -1.3760728
 24 N   N1           0.7119676     0.2579343     0.5164585
 25 O   O2           1.3103464     1.5199519     0.2950534
```

26	C	C6	1.4220960	1.8597677	-1.1665350
27	H	H6_1	2.4169543	1.5151644	-1.5068539
28	C	C14	1.2635209	3.3601802	-1.2221498
29	H	H14c	2.1323632	3.8697014	-0.7828639
30	H	H14b	0.3861589	3.6952317	-0.6480472
31	H	H14a	1.1454217	3.7143260	-2.2532977
32	O	O3	1.7449850	-0.5542154	0.9937845
33	Si	Si1	2.1894647	-1.2003926	2.4841446
34	C	C15	2.7651443	0.1323307	3.6349568
35	H	H15c	3.6347134	0.6651884	3.2202193
36	H	H15b	3.0459192	-0.2851070	4.6134352
37	H	H15a	1.9643276	0.8746800	3.7884086
38	C	C16	3.5770471	-2.3410189	2.0015908
39	H	H16c	4.3786766	-1.7937560	1.4833206
40	H	H16b	3.2298500	-3.1446406	1.3364308
41	H	H16a	4.0010615	-2.7995152	2.9094996
42	C	C17	0.7669831	-2.1294890	3.2321670
43	H	H17c	1.0851885	-2.6036551	4.1743706
44	H	H17b	0.3920154	-2.9156818	2.5621010
45	H	H17a	-0.0651940	-1.4440191	3.4559833
